# Eradication of pre-clinical prostate tumors by a lentiviral vector-based immunotherapy

**DOI:** 10.1016/j.omton.2026.201299

**Published:** 2026-07-18

**Authors:** Benjamin Vesin, Ingrid Fert, Sylvain Ciret, Laëtitia Douguet, Fanny Moncoq, Amandine Noirat, Pierre Authié, Fabien Le Chevalier, Fabien Nevo, Catherine Blanc, Kirill Nemirov, Laleh Majlessi, Pierre Charneau

**Affiliations:** 1 Pasteur-TheraVectys Joint Lab, Institut Pasteur, Université de Paris, Virology Department, 28 Rue Du Dr. Roux, 75015 Paris, France

**Keywords:** immuno-oncotherapy, lentiviral vectors, prostate cancers, prostate-associated antigen, t cell vaccines, prostatic acid phosphatase, tumor microenvironment, inflammation, immune memory, tumor relapse

## Abstract

Prostate cancer kills ≈350,000 yearly. While localized cases have 99% five-year survival, metastatic prostate cancer is hard to treat, with few options for tumors resistant to androgen deprivation. To leverage the immune system to fight prostate cancer, we developed a non-integrative lentiviral vector, namely “Lenti-PROST-02”, which encodes clusters of T cell immunodominant regions of human prostatic acid phosphatase and prostate-specific antigen. Immunotherapy with Lenti-PROST-02 in a preclinical virulent prostate tumor model resulted in complete tumor eradication in vast majority of the treated animals. This antitumor effect was concomitant with induction of poly-functional CD8^+^ T splenocyte effectors against numerous T cell epitopes of the antigens encoded by Lenti-PROST-02, increased proportions of tumor-infiltrating CD8^+^ T cells with activated/differentiated/effector phenotype and a “cold-to-hot” inflammatory switch of the tumor microenvironment. Immunity induced by Lenti-PROST-02 was long-lasting and prevented tumor relapse. It was characterized by the persistence of CD44^+^ CD62L^−^ CD127^+^ KLRG1^−^ CD8^+^ memory T cells in secondary lymphoid organs, as well as an antigen-diversified memory response. Therefore, Lenti-PROST-02 therapeutic vaccine is a promising approach for prostate immuno-oncotherapy.

## Graphical abstract

**Figure:**
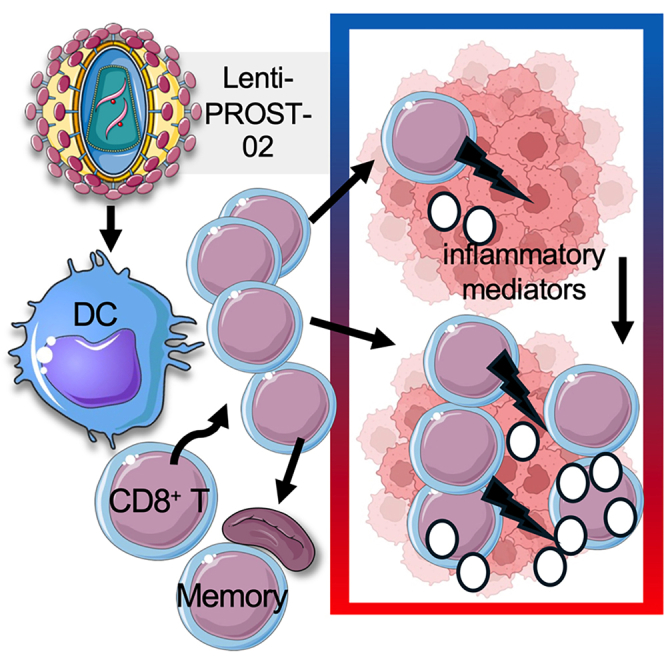

## Teaser

Majlessi, Charneau, and colleagues developed an immunotherapy named «Lenti-PROST-02» using a lentiviral vector platform to target prostate tumors. In a preclinical model, this approach triggered a robust cellular immune response against key prostate antigens. The expansion of this immunity within tumors was linked to microenvironment remodeling and tumor clearance.

## Introduction

Prostate cancer is the second most common cancer in men after lung cancer, representing 15% of new diagnoses in high-income countries and causing over 350,000 annual deaths.^1^ At diagnosis, ∼80% of cases remain prostate confined, 15% show locoregional spread, and 5% present distant metastases.^2^ Localized disease has a favorable prognosis, with five-year survival rates nearing 99% post-treatment.^3^ However, standard therapies—surgery, radiotherapy, or hormone deprivation—often impair quality of life. Metastatic progression, typically to lymph nodes, bone, or viscera, severely limits curative options, reducing treatment to palliative care. Advanced disease initially responds to androgen-deprivation therapy, defining metastatic castration-sensitive prostate cancer (mCSPC), where survival drops to ∼30% at five years.^4^ Inevitably, resistance to androgen-deprivation therapy develops, marking the transition to metastatic castration-resistant prostate cancer (mCRPC),^5^ a stage with few therapeutic options and poor outcomes.^6^

Immunotherapies targeting cytotoxic T-lymphocyte antigen 4 (CTLA-4), and/or programmed death-1 (PD1) or PD1 ligand (PDL1) have revolutionized immuno-oncotherapy but show limited efficacy in prostate cancer due to its “cold,” lymphocyte-sparse and immunosuppressive microenvironment.^7^ The FDA/EMA-approved “Sipuleucel-T”, an autologous dendritic cell (DC)-based vaccine,^8^^,^^9^^,^^10^ provided more encouraging results than immune checkpoint inhibitors, but still induced only partial clinical benefits, motivating development of more efficient T cell-based therapies.^11^

Non-integrative lentiviral vectors are effective in inducing long-lasting, high-quality T cell responses,^12^^,^^13^^,^^14^ with proven safety in clinical trials (2011-006260-52).^15^^,^^16^ Recent preclinical studies demonstrated their efficacy in human papilloma virus (HPV)-driven cancers, achieving 100% tumor eradication and long-term immunity.^17^^,^^18^ A cross-sectional comparative review of the most relevant immuno-oncotherapy strategies, evaluated in the same HPV-induced murine cancer model, established that certain adjuvanted proteins and viral vectors showed the most robust antitumor effects. Among them, the lentiviral vectors were the only strategy resulting in full tumor eradication in 100% of animals with the longest-lasting immune memory able to prevent tumor relapse.^19^ This success led to a phase 1/2a clinical trial for HPV-related cancers (NCT06319963) and prompted evaluation of lentiviral vector platform efficacy in prostate cancer, a non-viral malignancy.

Based on their overexpression and enrichment in prostate tumors, the most pertinent targeted antigens in these cancers include (1) prostatic acid phosphatase (PAP), (2) prostate-specific antigen (PSA), (3) prostate-specific membrane antigen (PSMA), and (4) prostate stem cell antigen (PSCA).^11^ Unlike PSMA and PSCA, which exhibit off-target expression in non-prostatic tissues (e.g., PSMA in brain and PSCA in the bladder, kidney, stomach, and pancreas), PAP and PSA demonstrate far greater prostate specificity, minimizing the risk of off-target immune responses.^20^^,^^21^^,^^22^ We thus selected PAP and PSA to include in a lentiviral vector-based immunotherapy. The inclusion of two prostate antigens mitigates the probability of tumor escape linked to a possible loss of expression of a single targeted antigen. PAP glycoprotein is expressed on >95% of prostate cancer cells, and is included in Sipuleucel-T. PSA is a neutral serine protease, a serum marker followed in men to screen the risk of developing prostate cancer or to monitor the disease progression or recurrence after initial diagnosis.^20^ We generated a non-integrative lentiviral vector, namely “Lenti-PROST-02”, which encodes clusters of predicted or previously identified T cell epitopes of human PAP and PSA, under the transcriptional control of the human β2 microglobulin promoter (β2m).^12^ We have previously demonstrated that following intramuscular (i.m.) administration of lentiviral vectors encoding a reporter antigen under β2m promoter, multiple immune cell populations in draining lymph nodes become transduced within 5 days. The highest transduction efficiency is observed *in vivo,* at 2%–5% antigen-positive cells in macrophages and DC subsets (including conventional CD8^+^ and CD8^−^ DCs and plasmacytoid DCs), while T and B cells exhibit minimal (<1%) transduction. Upon immunization with a lentiviral vector encoding a model antigen under β2m promoter, all DC subsets efficiently prime antigen-specific CD8^+^ T cells, but macrophages, B cells, and T cells do not. These results highlight DCs as the primary antigen-presenting cells responsible for initiating CD8^+^ T cell immunity following i.m. delivery of lentiviral vector using the β2m promoter.^12^

In a preclinical virulent prostate cancer model set up here, Lenti-PROST-02 elicited robust CD8^+^ T cell responses, inducing complete tumor regression in the great majority of treated mice. Critically, it established long-term immune memory, preventing relapse upon tumor re-challenge. Mechanistically, Lenti-PROST-02 therapy reprogrammed the tumor microenvironment by increasing CD8^+^ tumor-infiltrating lymphocytes (TILs) with effector phenotypes, shifting “cold-to-hot” inflammation, and sustaining memory precursor T cells in the spleen. These data validate lentiviral vector-based immunotherapy as a promising strategy for prostate cancer, warranting clinical translation.

## Results

### PROST-02 antigen design

**Figure 1:**
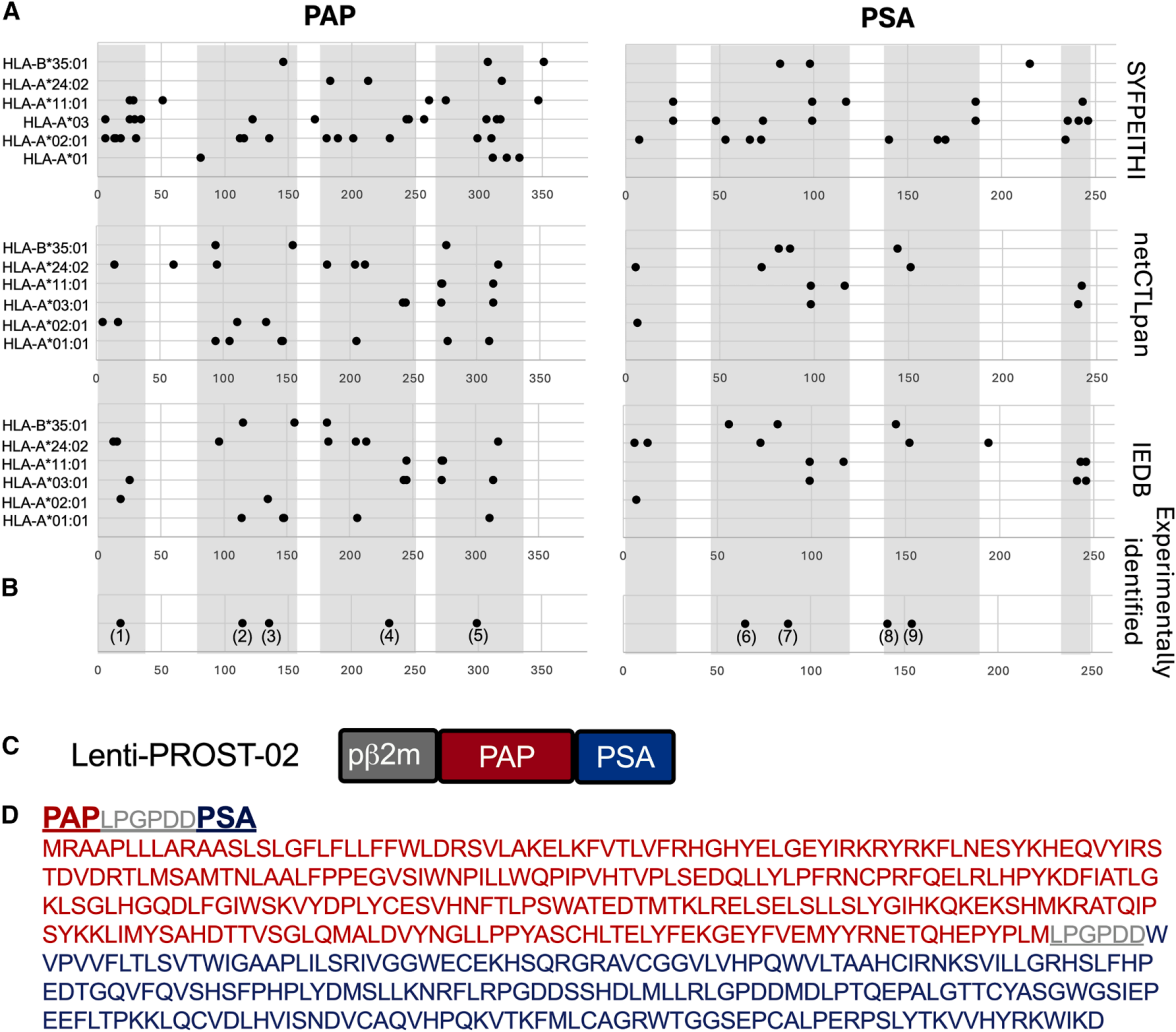
Selection of T cell epitope-containing regions of human PAP and PSA (A) Distribution of predicted human MHC-I PAP- or PSA-specific epitopes by use of SYFPEITHI with a score >21, netCTLpan with a score <1, and IEDB with a score <1, applied to HLA-B∗35:01, HLA-A∗24:02, HLA-A∗11:01, HLA-A∗03, HLA-A∗02:01, and HLA-A∗01 alleles. (B) Experimentally identified human T cell epitopes as referenced in IEDB database for (1) HLA-A2, (2) HLA-DRB1∗01:01, (3) HLA-A∗02:01, (4) HLA-DR1, (5) HLA-A2, (6) HLA-DRB1∗04:01, (7) H-2D^b^, (8) HLA-A2, and (9) HLA-A2. Each dot corresponds to the first aa of an epitope and its position along the sequence of PAP or PSA (*x* axis). The regions highlighted in gray were selected to be included in the “PROST-02” antigen. (C) PROST-02 antigen composed of clusters of T cell epitopes derived from PAP (red) and PSA (blue), under the human β2m promoter, as encoded by Lenti-PROST-02 non-integrative vector. (D) Protein sequence of PROST-02 antigen containing 505 aa. The underlined gray characters indicate the linker between PAP and PSA segments.

Expression of the human PSA and PAP are highly specific to prostate glandular epithelial cells (Figures S1A and S1B) and increases with prostate cancer progression,^23^^,^^24^ making them relevant antigens for cancer immunotherapy, without entailing risks of off-tumor (multi)organ autoimmunity. To select the T cell immunogenic regions of PAP (Acc# P15309) and PSA (Acc# P07288) (Figure 1A), presentable by molecules of human leukocyte antigen of class-I (HLA-I), we used SYFPEITHI (http://www.syfpeithi.de), IEDB (https://www.iedb.org), and netCTLpan (https://services.healthtech.dtu.dk/services/NetCTLpan-1.1/) epitope prediction tools. The advantage of NetCTLpan is to provide a combined prediction score by integrating (1) strength of peptide-MHC-I binding, (2) efficacy of proteasomal C-terminal cleavage, and (3) efficacy of peptide transport by TAP (transporter associated with antigen processing). Initially, 9 amino acid (aa)-long peptides that could be presented by 6 common HLA alleles, i.e., HLA-A∗01:01, HLA-A∗02:01, HLA-A∗03:01, HLA-A∗11:01, HLA-A∗24:02, and HLA-B∗35:01, were searched by combining the results generated by these prediction tools (Figure 1B). To narrow down the list of potential epitopes, only those with the prediction score >21 in SYFPEITHI, <1 in IEDB and netCTLpan were selected. Both predicted (Figure 1B) and already identified (Figure 1C) epitopes were mapped to the reference sequences of PSA and PAP.

To further increase the potential immunogenicity of the polyantigen in the global human population, IEDB prediction was extended to 21 additional HLA-I alleles (Table S1). The total of 27 HLA-I alleles considered are shared by 97% of the human population. The vast majority of the resulted predicted epitopes was mapped to the same immunogenic regions identified previously, reinforcing the choice of potentially immunogenic regions. The selected immunogenic regions, which were prolonged with 3 aa residues on either side of each region, were then joined together. This resulted in 6 aa-long linkers. The resulting PAP-PSA fusion sequence was called “PROST-02”. Except for PSA and PAP, no significant homology was found for the PROST-02 clustered antigenic sequences versus complete human protein database (https://www.uniprot.org), as scanned by BLAST (https://blast.ncbi.nlm.nih.gov/Blast.cgi). This is reassuring regarding the risk of non-specific autoimmune responses in case of the use of these sequences in prospective immuno-oncotherapy. A small number of putative junctional epitopes have been generated, none of which showed particular homology with the human protein database.

A non-integrative lentiviral vector, named “Lenti-PROST-02”, was designed to encode the PROST-02 fusion antigen, under the transcriptional control of human β2m promoter (Figures 1D and S2).^12^ This promoter contains conserved *cis*-regulatory elements: (1) interferon (IFN)-stimulated response elements (ISREs) that are the binding site for the IFN regulatory factors, and (2) SXY modules which interact with a multiprotein complex. ISREs and SXY transactivate pro-inflammatory cytokines, which are upregulated in maturing DCs.^25^^,^^26^

### T cell immunogenicity of Lenti-PROST-02

T cell immunogenicity of Lenti-PROST-02 was evaluated by immunizing male C57BL/6 (H-2^b^) mice (*n* = 5/group) via i.m. route at day 0 with 1 × 10^9^ transduction units (TU) of Lenti-PROST-02 or an empty vector (Ctrl Lenti). On day 14, splenocytes from individual mice were used in IFNγ ELISPOT after *in vitro* stimulation with pools of 15-mer peptides spanning the complete sequences of the PROST-02 antigen.

**Figure 2:**
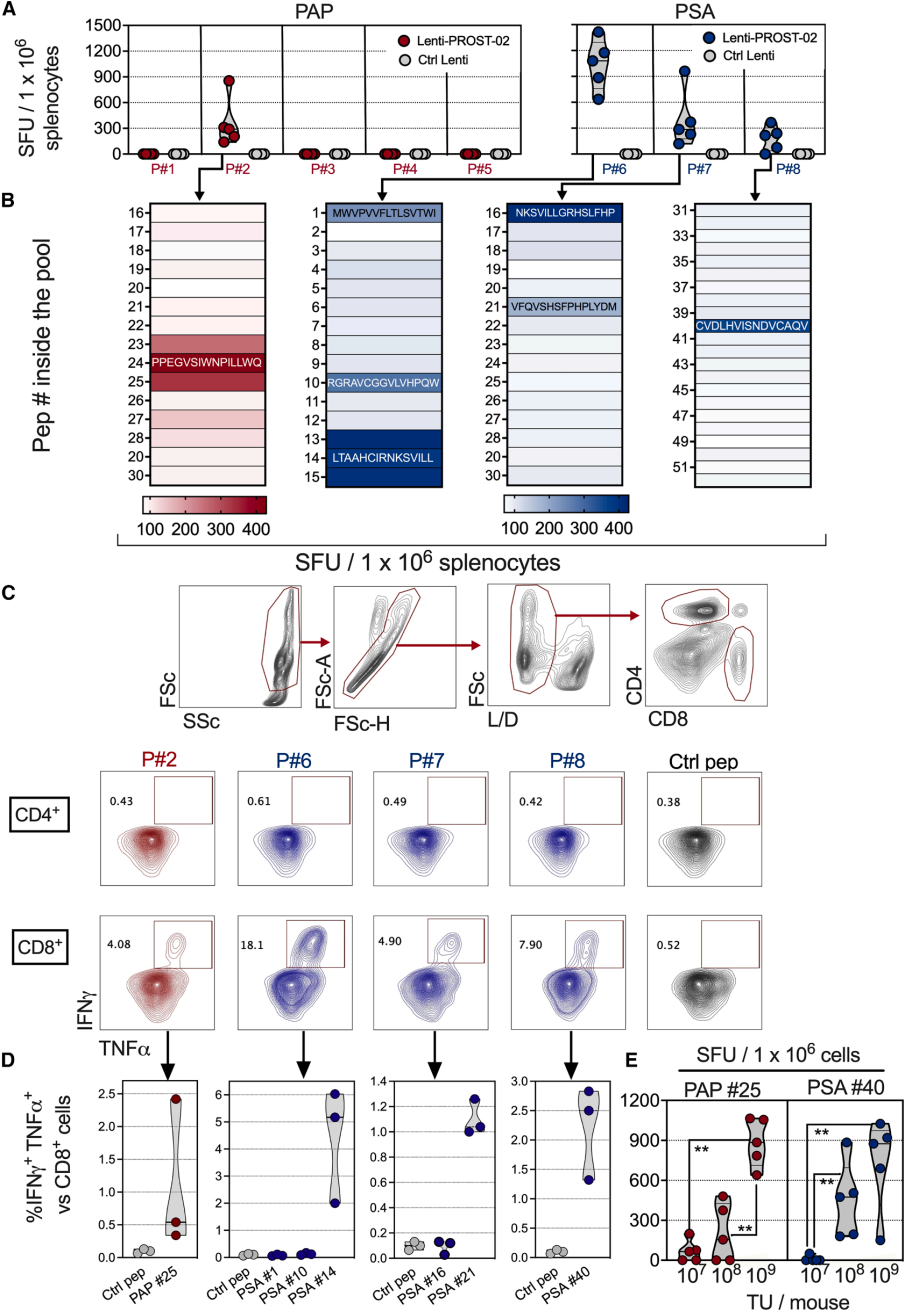
T cell immunogenicity of Lenti-PROST-02 (A) T cell responses of C57BL/6 mice, injected i.m. with 1 × 10^9^ TU of Lenti-PROST-02 or Ctrl Lenti (*n* = 5/group). Frequencies of specific T splenocytes, determined by IFNγ ELISPOT at day 14 post-injection, in individual mice after *in vitro* stimulation with 5 or 3 distinct pools of 15-mer peptides, spanning respectively the PAP or PSA PROST-02 segments, and overlapping each other by 10 aa. SFU, spot-forming units. (B) Incubation of splenocytes from Lenti-PROST-02-immunized mice with each individual peptide of the positive peptide pools and studied by IFNγ ELISPOT. Heatmaps show SFU/1 × 10^6^ splenocytes. The sequences of the immunogenic 15-mers are indicated inside the corresponding heatmap cells. (C) Cytometric gating strategy (top) and CD4^+^ or CD8^+^ T-splenocyte cytokine responses in immunized mice, as studied by ICS, after stimulation with derived from PAP or PSA (bottom). One representative mouse of 6/group is showed for each peptide. “L/D” = Live-Dead. (D) The same gating strategy as in (C) was applied. ICS-based study of IFNγ^+^ TNFα^+^ CD8^+^ T cell response of 3 individual Lenti-PROST-02-immunized mice to individual positive peptides from positive pools. (E) Dose-response of mice (*n* = 5/group), immunized i.m. with Lenti-PROST-02 escalating doses. T-splenocyte responses were studied by IFNγ ELISPOT at day 11 post-immunization. The results are representative of two independent experiments.

Splenocytes from the control mice did not respond to the peptide stimulation, while those from the Lenti-PROST-02-immunized mice were responsive to one PAP-derived and three PSA-derived peptide pools (Figure 2A). Subsequent incubation of splenocytes with each individual peptide of the positive pools allowed precise epitope mapping of the PROST-02 antigen and identification of at least 6 immunogenic regions in H-2^b^ mice (Figure 2B). Intracellular cytokine staining (ICS) of splenocytes from Lenti-PROST-02-immunized mice showed that the responding splenocytes were CD8^+^ T cells, containing a majority of IFNγ^+^ TNFα^+^ cells (Figure 2C). By use of ICS, we identified the strongest individual peptides from each positive pool (Figure 2D) for further immunological studies. The weak PSA-derived epitopes (PSA #1 and #10) detected by ELISPOT were not detected by ICS, which is less sensitive (Figure 2D). The good immunogenicity of Lenti-PROST-02 in male mice against PAP #25 and PSA #21 peptides, which have strong homology between the murine and human sequences (Table S2; Figure S3), supports a vaccine-induced break in the expected natural tolerance against these “self” epitopes.

Immunization of C57BL/6 mice with escalating doses of Lenti-PROST-02 showed that the induced T cell immunity was dose dependent and that the dose of 1 × 10^9^ TU generated the strongest responses (Figure 2E), in accordance with previous findings using other antigens.^17^^,^^27^

### Lenti-PROST-02-based immunotherapy against a preclinical prostate cancer model

**Figure 3:**
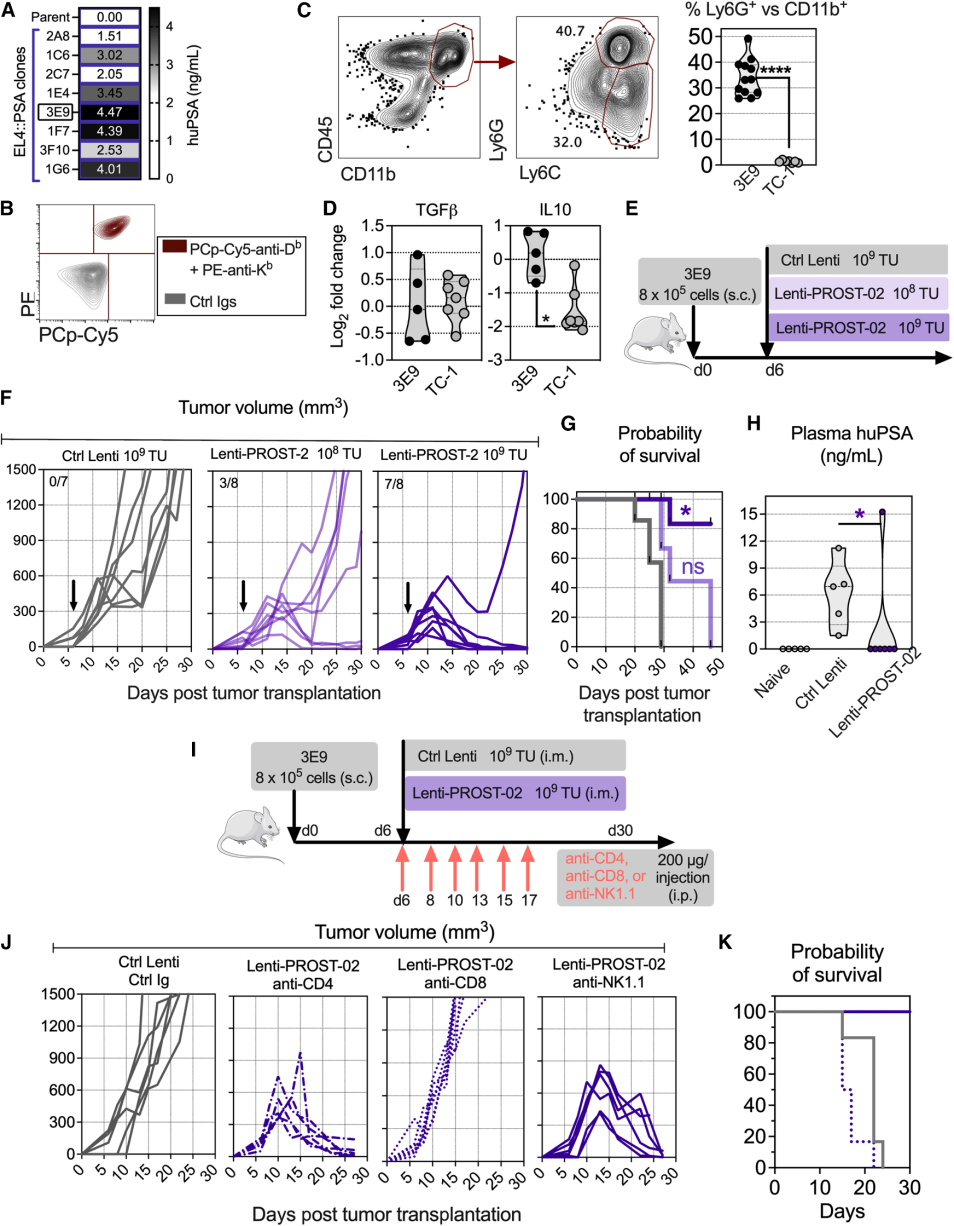
Lenti-PROST-02-based immunotherapy of tumors expressing human PSA (A) Generation of a preclinical prostate tumor model. EL4 cells were transduced by an integrative lentiviral vector encoding the full-length human PSA, cloned and screened for the secretion of PSA. The clone “3E9” was selected for further experiments. (B) Cytometric profile of surface H-2D^b^ and H-2K^b^ expression of 3E9. (C) Cytometric gating strategy and proportions of intratumor Ly6G^+^ Ly6C^−^ cells within the CD11b^+^ subset, determined on day 13 after 3E9 or TC-1 transplantation (s.c.) in C57BL/6 mice. (D) Log_2_ fold change in TGFβ and IL10 mRNA expression in the 3E9 or TC-1 tumors. (E) Timeline of tumor engraftment and immunotherapy. C57BL/6 male mice (*n* = 7/group) were engrafted s.c. on the right flank with 8 × 10^5^ 3E9 cells. At day 6 post-tumor engraftment, mice were randomized for their tumor size and immunized i.m. with 1 × 10^8^ or 1 × 10^9^ TU of Lenti-PROST-02. Control mice received 1 × 10^9^ TU of Ctrl Lenti. (F) Tumor size and (G) survival over the time. Concentration of hPSA in the plasma of naive controls or mice described in (E), determined on day 21. Note that 2 of 7 Ctrl Lenti-treated mice were already sacrificed at the time of blood sampling for this analysis due to tumor size exceeding 1,500 mm^3^. Statistical significance for (C, D, and H) was determined by two-tailed Mann-Whitney test (∗*p* < 0.05, ∗∗∗∗*p* < 0.0001). The results shown in (C and D) are representative of two independent experiments. Those shown in (F and G) are representative of at least ten experiments. (I) Experiment timeline for *in vivo* phenotyping of anti-tumor cell effectors. C57BL/6 male mice (*n* = 6/group) were engrafted s.c. on the flank with 8 × 10^5^ 3E9 tumor cells. On day 6 post-tumor engraftment, mice were injected i.m. with Lenti-PROST-02 or Ctrl Lenti. Starting on day 6, mice received intraperitoneal injections of 200 μg/mouse/injection of anti-CD4 (clone GK1.5), anti-CD8 (clone H35.17.2), anti-NK.1 (PK136) mAb or Ctrl Ig every 2 or 3 days. (J) Tumor volume versus time. (K) Survival over the time. Statistical significance for (G and K) was determined by Log rank Mantel-Cox test (∗*p* < 0.05). Mice were sacrificed when tumors reached 1,500 mm^3^.

To evaluate the antitumor effect of Lenti-PROST-02, we started with the murine prostate tumor model TRAMP-C1,^28^ an epithelial cell line isolated from the prostate of a transgenic mouse. However, these cells transduced to express human PSA or human PAP failed to develop tumors in male C57BL/6 recipients at any level of transgene expression, in contrast to the corresponding parental line. We also considered the RM-1 tumor model,^29^ a prostate cell line derived from prostate cancer induced by Ras and Myc gene activation. However, RM-1 model presents a significant limitation as it is entirely MHC-I-deficient at the basal level, a phenotype far more extreme than that observed in human prostate tumors, which typically exhibit partial rather than complete MHC-I downregulation.^30^^,^^31^ This led us to use the C57BL/6 syngeneic EL4 tumor line, which is commonly used in preclinical cancer models, even if it does not fully recapitulate all features of human prostate cancer. We transduced EL4 cells to stably produce the full-length human PSA. The “3E9” clone was selected for its highest capacity in PSA secretion and its MHC-I surface expression (Figures 3A and 3B). At least some sub-populations of neutrophils/granulocytes support tumor progression by favoring angiogenesis, stimulating tumor cell migration and promoting an immunosuppressive tumor microenvironment.^32^ In this respect, subcutaneous (s.c.) 3E9 tumors, compared to the standard and well characterized model of HPV-induced TC-1 tumors, were particularly rich in CD11b^+^ Ly6C^int^ Ly6G^+^ neutrophils/granulocytes (Figure 3C). In addition, 3E9 tumors had higher contents of IL10 (but not TGFβ) mRNA compared to the TC-1 tumors (Figure 3D).

Therefore, 3E9 model can, at minimum, recapitulate certain features of the cold and immunosuppressive tumor microenvironment observed in human prostate cancers.

The antitumor efficacy of Lenti-PROST-02 was assessed in male mice engrafted (s.c.) with 3E9 tumor cells (*n* = 7–8/group). Six days after tumor inoculation, when the tumors were well established, mice were treated by a single i.m. injection of 1 × 10^8^ or 1 × 10^9^ TU of Lenti-PROST-02 (Figure 3E). Tumor-bearing control mice received 1 × 10^9^ TU of Ctrl Lenti. In all control mice, tumor volume reached 1,500 mm^3^, defined as humane endpoint, before day 30. In opposite, an antitumor effect was detected in Lenti-PROST-02-treated mice in a dose-dependent manner (Figure 3F). The high dose of 1 × 10^9^ TU led to a complete tumor eradication in 7/8 mice. Full tumor eradication was also observed in 3/8 mice with the suboptimal 1 × 10^8^ TU dose (Figure 3F). The survival of the mice immunized with 1 × 10^9^ TU of Lenti-PROST-02 was significantly prolonged (Figure 3G). The active secretion of human PSA by 3E9 tumors resulted *in vivo* in the presence of this antigen in the bloodstream, as measured on day 21 post-tumor transplantation (Figure 3H). Human PSA was not measurable in the blood of Lenti-PROST-02-treated mice, except for one mouse in which the tumor had not been eradicated. Therefore, as with physiological prostate cancers, in this preclinical model, PSA blood levels are an appropriate biomarker, reflecting the prostate tumor status.

The critical role of CD8^+^ T cells in Lenti-PROST-02-induced tumor clearance was established using antibody depletion experiments. In 3E9 tumor-bearing mice, efficient depletion of CD8^+^ T cells, but not CD4^+^ T cells or NK cells, abolished vaccine-mediated tumor eradication (Figures 3J, 3K, and S4).

Among our numerous attempts in several syngeneic tumor lines to generate cell lines expressing human PAP and capable of forming solid tumors, only a clone of the MC38 line (colorectal tumor model)^33^ transduced to express this protein (“MC38-PAP”) could be selected. The anti-tumor efficacy of the lentiviral vector-based vaccine against MC38-PAP was demonstrated, especially when this treatment was combined with an anti-PD1 antagonist mAb (Figure S5). This does not necessarily mean that this therapy should be combined with an anti-PD1 mAb, but strengthens the preclinical proof-of-concept that the anti-huPAP T cells induced by the vaccine have an anti-tumor effector potential, even if the designed PAP segment only has one immunogenic region for C57BL/6 (H-2^b^) mice.

These results demonstrated the antitumor potential of Lenti-PROST-02 immunotherapy in virulent prostate tumor models.

### Impact of Lenti-PROST-02-treatment on intra-tumoral immune cells

**Figure 4:**
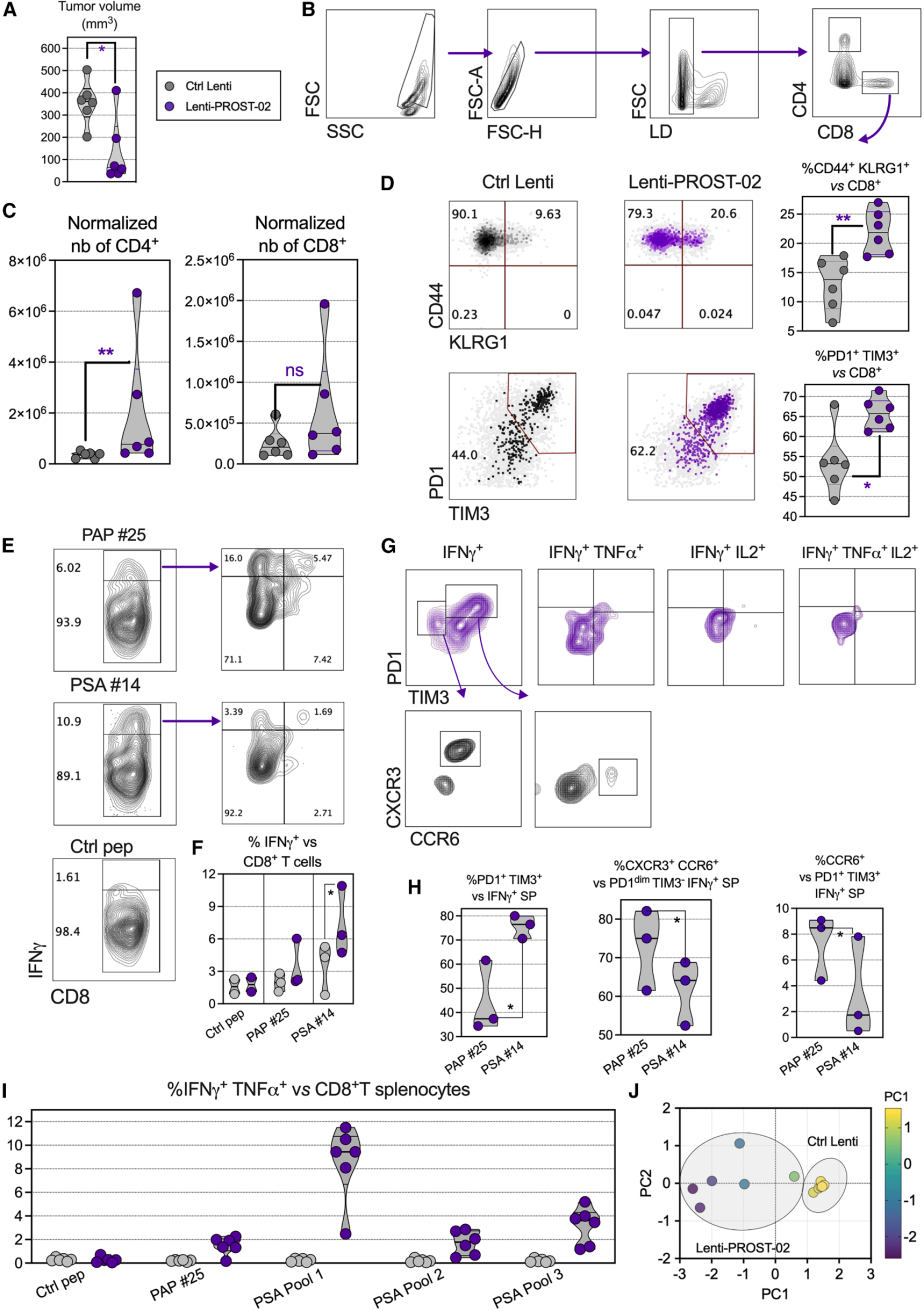
Status of TILs and T splenocyte responses induced by Lenti-PROST-02 C57BL/6 mice were engrafted s.c. with 3E9 cells on day 0 and treated with Lenti Ctrl or Lenti-PROST-02 on day 6 (*n* = 6/group). TILs were studied by cytometry on day 15, i.e., 9 days post-vaccination. (A) Tumor volume on day 9 post-vaccination. (B) The cytometric gating strategy. Note that 3E9 tumor cells express CD45 themselves and thus it was not possible to draw a gate on the CD45^+^ hematopoietic cells and calculate the % of CD4^+^ or CD8^+^ TILs versus the CD45^+^ population. (C) Absolute numbers of CD4^+^ or CD8^+^ TILs normalized to tumor volume. (D) Representative dot plots of CD8^+^ TILs and their differentiation status in both groups and recapitulative percentages of various TIL subsets studied comparatively in both groups. (E) TILs were co-cultured with syngeneic bone-marrow-derived DC loaded with 10 μg of PAP #25 (top), PSA #14 (middle), or an irrelevant (bottom) peptide. Cells were stained at the surface and intracellular effector cytokines. To reach sufficient number of TILs per analysis, samples were pooled 2 by 2. Representative dot plots to detect various cytokine-expressing CD8^+^ in Lenti-PROST-02 mice after *in vitro* restimulation with PAP#25, PSA#14, or an irrelevant peptide. (F) Percentages of total antigen-specific IFNγ^+^ cells versus CD8^+^ TILs. (G) PD1 versus TIM3 expression in each functional subsets (top) and CXCR3 versus CCR6 expression in various subsets of IFNγ^+^ SP CD8^+^ TILs after *in vitro* stimulation. (H) Recapitulative %PD1^+^ TIM3^+^ versus IFNγ^+^ SP, %CXCR3^+^ CCR6^+^ versus PD1^dim^ TIM3^-^ IFNγ^+^ SP, and %CCR6^+^ versus PD1^+^ TIM3^+^ IFNγ^+^ SP CD8^+^ TILs subset in PAP- or PSA-specific CD8^+^ cells. (I) PAP- and PSA-specific IFNγ^+^ TNFα^+^ T splenocyte responses. (J) PCA projection of all parameters studied in (A–I). Proportion of variance for PC1 = 87.62%. Results are representative of two independent experiments. Statistical significance was evaluated using Mann-Whitney tests (∗*p* < 0.05).

To get more insights on the Lenti-PROST-02 mode of action, we studied the tumor cell microenvironment after the treatment. As early as day 9 after a single i.m. administration of 1 × 10^9^ TU of Lenti-PROST-02, when the tumor shrinkage began (Figure 4A), the absolute numbers of CD4^+^ or CD8^+^ TILs normalized to tumor volume were significantly higher or showed an increasing trend respectively, compared to those in the growing tumors of control mice (Figures 4B and 4C). In the shrinking tumors of Lenti-PROST-02-treated mice, the CD8^+^ T subset contained significantly higher proportions of CD44^+^ KLRG1 (killer-cell lectin like receptor)^+^ cells and PD1^+^ TIM3 (T cell immunoglobulin domain and mucin domain)^+^ cells, compared to the CD8^+^ T subset in the control mice (Figure 4D). This predominance of activated/terminally differentiated CD44^+^ KLRG1^+^ and PD1^+^ TIM3^+^ CD8^+^ T cells in the shrinking tumors likely resulted from their continuous antitumor activity.

In day 9 after Lenti-PROST-02 treatment, vaccine-induced PAP- or vaccine/tumor-induced PSA-specific CD8^+^ TILs were detected after a 6 hour-coculture of TILs with syngeneic DCs pulsed with relevant PAP- (Figure 4E, top) or PSA- (Figure 4E, middle) derived peptides. Low levels of PSA-specific CD8^+^ tumor-borne TILs were also detectable in control mice (Figure 4F). Lenti-PROST-02-treated mice had significantly higher levels of PSA-specific CD8^+^ TILs, which were thus both tumor-borne and vaccine-induced (Figure 4F).

Among antigen-specific functional CD8^+^ TIL subsets in Lenti-PROST-02-treated mice IFNγ^+^ single-positive (SP) cells were largely predominant in both PAP- or PSA-specific CD8^+^ TILs (Figure 4E). They displayed mainly a PD1^+^ TIM3^+^ activated/differentiated/exhausted phenotype (Figure 4G, top). Notably, the proportions of PD1^+^ TIM3^+^ cells inside IFNγ^+^ SP subset were significantly higher in the PSA-specific T cells than in PAP-specific T cells (Figure 4H, left). This trait may result from a continuous triggering of PSA-specific CD8^+^ TILs and sustained exercise of their effector functions against the PSA- (but not PAP-) expressing 3E9 tumors.

Antigen-specific IFNγ^+^ SP population with PD1^dim^ TIM3^−^ phenotype contained a CXCR3^+^ CCR6^+^ subset (Figure 4G, bottom left), with presumable central/effector memory features. In parallel, antigen-specific IFNγ^+^ SP subset with PD1^+^ TIM3^+^ phenotype contained a CCR6^+^ subset (Figure 4G, bottom right), with possible memory properties. These two PD1^dim^ TIM3^−^ CXCR3^+^ CCR6^+^ (Figure 4H, middle) and PD1^+^ TIM3^+^ CCR6^+^ (Figure 4H, right) subsets were less represented in the PSA-specific subset than in the PAP-specific subset, probably because PSA-specific T cells had more effector than memory features.

On day 9 after Lenti-PROST-02 treatment, IFNγ^+^ TNFα^+^ CD8^+^ T splenocytes specific for immunogenic regions of the PROST-02 fusion antigen were readily detectable (Figure 4I). These splenocyte responses were vaccine- but not tumor induced, as tumor-bearing control mice did not show such responses in their spleen (Figure 4I). Of note, despite the capacity of 3E9 tumor cells to secrete PSA *in vitro* (Figure 3A) and *in vivo* (Figure 3H), engraftment of these tumors per se did not induce PSA-specific CD4^+^ T splenocyte responses either (Figure S6A). Principal component analysis (PCA) applied to the T cell responses to Lenti-PROST-02, the tumor size and the proportions of activated TILs showed that the group of mice with shrinking tumors clustered away from that of mice bearing growing tumors (Figure 4J).

The frequencies of granulocytes/neutrophils, which were widely represented in the 3E9 tumors (Figure 3C), and those of NK (CD11b^int^ NKP46^+^) cells, were statistically similar in tumors from both experimental groups (Figures S6B and S6C). The intratumor CD11b^+^ F4/80^hi^ CD11c^+^ MHC-II^+^ population containing a CD24^+^ cell subtype, which may correspond to a previously described monocyte-derived macrophage/cDC2 subset,^34^ was well detected in the 3E9 tumors. Their frequencies were not statistically distinct in the two groups (Figure S6D).

In sum, a single Lenti-PROST-02 i.m. administration induced antigen-specific CD8^+^ TILs with strong effector/differentiated phenotypes in 3E9 tumor-bearing mice.

### Inflammatory status of tumors in Lenti-PROST-02-treated mice

**Figure 5:**
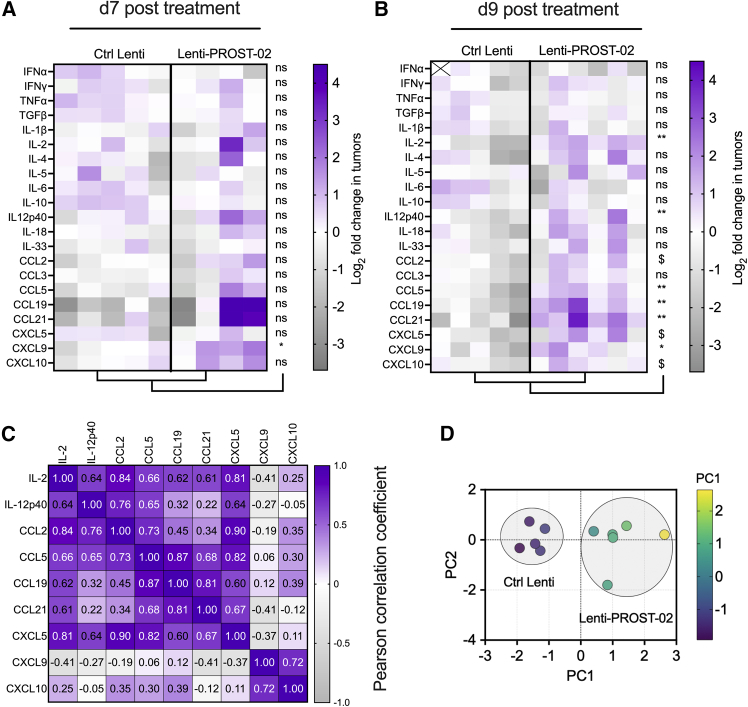
Inflammatory status of the 3E9 tumors in control or Lenti-PROST-02-treated mice (A and B) Log_2_ fold change in mRNA expression in the tumors of mice (*n* = 5 or 4–6/group), treated with Ctrl Lenti or Lenti-PROST-02 on day 6, and analyzed by RT-PqCR on day 7 (A) or 9 (B) post-vaccination. For the control and Lenti-PROST-02 groups, Mean ± SD of tumor volumes were respectively 218 ± 145 and 244 ± 258 (statistically not different) at day 7 and 357 ± 98 and 135 ± 148 (statistically different, ∗*p* < 0.05) at day 9 post-vaccination. For each sample, the mRNA abundance (*C*_T_ value) of the target genes was normalized to that of the succinate dehydrogenase complex, subunit A (SDHA) gene and compared to a calibrator value corresponding to the mean of the *C*_T_ values determined in the tumors from the Ctrl Lenti mice. The fold change in gene expression was then calculated using 2^−ΔΔ*C*^_T_. Statistical significance was evaluated using Mann-Whitney tests (ns: not significant, ^$^*p* < 0.1, ∗*p* < 0.05, ∗∗*p* < 0.01). One outlier sample was discarded from the analyses in the group of Ctrl Lenti for IFNα analyte. (C) Pearson correlation coefficient of differentially expressed analytes in the tumors of Lenti-PROST-02-treated mice on day 9 post-vaccination. (D) PCA projection of all analytes studied in (B). Proportion of variance for PC1 = 62.01%. Results shown are representative of two independent experiments.

The inflammatory status of the 3E9 tumors in the Ctrl Lenti- or Lenti-PROST-02-treated mice was analyzed by quantitative reverse-transcription PCR (RT-qPCR) on total tumor RNA. On day 7, no significant difference in the expression levels of inflammation-related analytes was observed between the two groups, with the exception for CXCL9, which exhibited elevated levels in the Lenti-PROST-02 group (Figure 5A). On day 9, in Lenti-PROST-02-treated mice, the tumors displayed globally a more inflammatory profile than in Ctrl Lenti-treated mice (Figure 5B). The expression of IL2 and IL12p40 was higher in the tumors of Lenti-PROST-02-treated mice (Figure 5B), and in a correlative manner (Figure 5C). In these mice, the higher intratumor expression of CCL2 and CCL5 (Figure 5B), in a correlative manner (Figure 5C), can contribute to tumor immune infiltration.^35^ The increased expression of CCL19 and CCL21 can promote IFNγ-dependent antitumor immunity by enhancing the homing of CCR7^+^ DCs and T cells.^36^ The increased expression of CXCL5 may chemo-attract T cells and neutrophils and contribute to angiogenesis.^37^ The upregulation of IFNγ-induced CXCL9 and CXCL10 is well known to cause recruitment of activated CD8^+^ T cells.^38^ The PCA projection of all the analytes studied on day 9 post-treatment divided the control and Lenti-PROST-02 individuals into two distinct groups (Figure 5D).

Therefore, a single i.m. Lenti-PROST-02 injection triggered a “cold-to-hot” tumor microenvironment shift by day 9, aligning with the kinetics of lentiviral vector-induced CD8^+^ T cells, detectable ∼8–9 days post-vaccination.^12^ This switch correlated with expanded activated/differentiated CD8^+^ TILs and tumor regression, likely driven by vaccine-elicited CD8^+^ T cell activity. This state is widely considered to be indicative of a favorable prognosis regarding to immune control of tumor growth.^39^

### Long-term protective immune memory induced by Lenti-PROST-02 therapy

**Figure 6:**
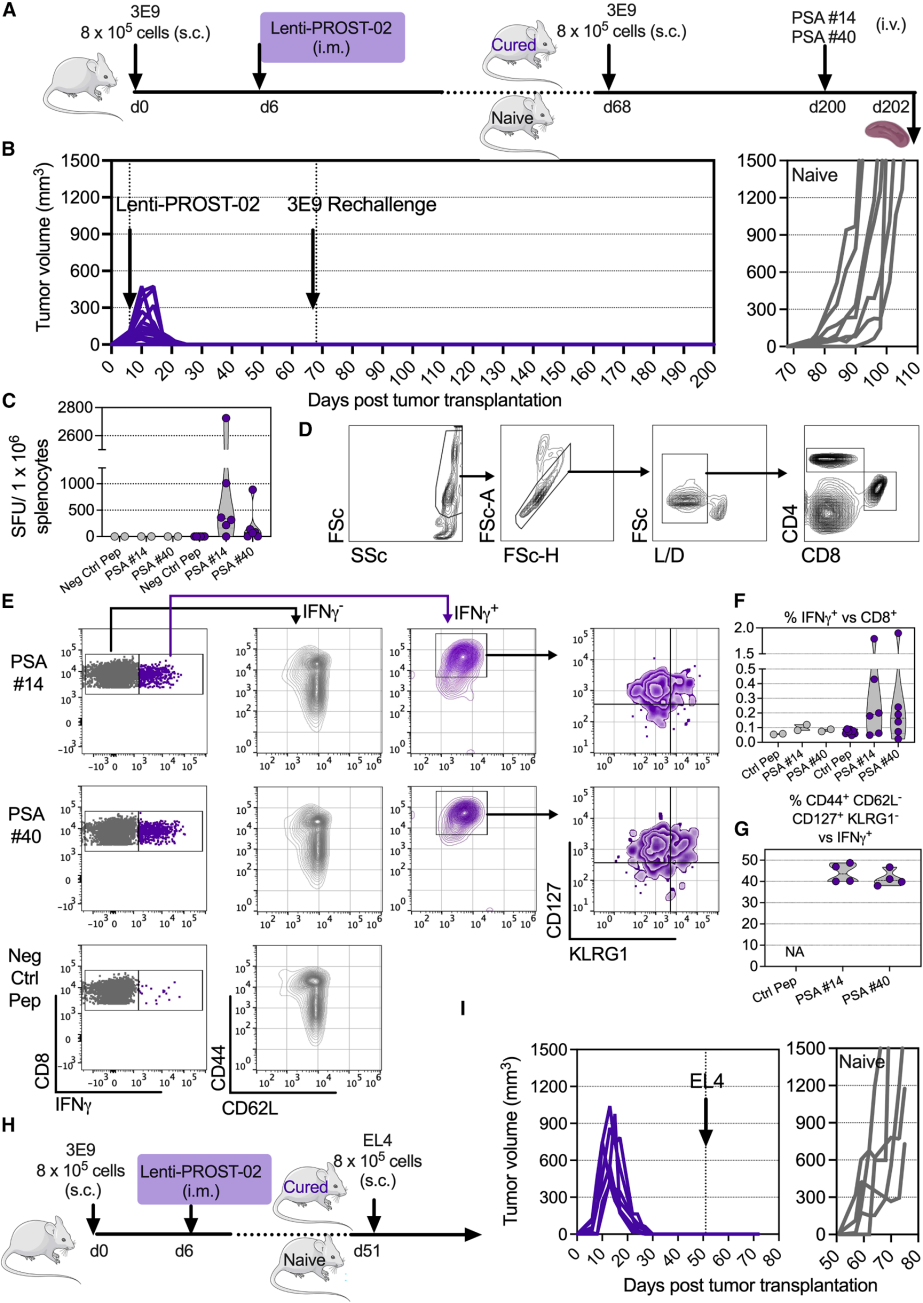
Lenti-PROST-02 therapy induces a long-term antitumor immune memory which prevents tumor relapse (A) Timeline of the tumor engraftment and immunotherapy. C57BL/6 mice (*n* = 13) were engrafted s.c. on the right flank with 8 × 10^5^ 3E9 cells. At day 6 post-engraftment, mice were randomized and treated i.m. with 3 × 10^8^ TU of Lenti-PROST-02. (B) Tumor growth. On day 68, the cured mice were rechallenged with 8 × 10^5^ of 3E9 cells in the opposite flank and left untreated to mimic a prostate tumor relapse. Naive mice (*n* = 7), injected s.c. with the same 3E9 cell suspension showed the growth potential of the engrafted tumor cells used in the relapse experiment (B, right). The cured mice in which the tumor relapse was unable to occur received on day 200 an i.v. infusion of a mixture of 25 μg of PSA #14 and 25 μg of PSA #40 peptides. Two days later, splenocytes were recovered and stimulated *in vitro* with homologous peptides or an irrelevant peptide. (C) Frequencies of PSA#14- or PSA#40-specific T splenocytes, determined by IFNγ ELISPOT. (D and E) Cytometric gating to detect IFNγ-producing PSA-specific CD8^+^ T cells. (F) Percentages of PSA-specific IFNγ^+^ cells compared to total CD8^+^ T splenocytes. (G) Percentages of CD44^+^ CD62L^−^ CD127^+^ KLRG1^−^ memory precursor cells versus IFNγ^+^ CD8^+^ T cells in the 4 mice with antigen-specific response detectable by ICS. NA = not applicable. Results shown in (B) are representative of three independent experiments. (H) Experiment timeline. Cured mice (*n* = 7) were rechallenged on day 51 with 8 × 10^5^ of PSA-negative parental EL4 cells in the opposite flank and left untreated. Naive mice (*n* = 6), injected s.c. with the same EL4 cell suspension showed the growth capacity of the engrafted cells. (I) EL4 tumor growth in cured (left) or naive control (right) mice.

We determined whether the tumor-cured mice developed a protective immune memory. After an initial 3E9 tumor engraftment (s.c.) (*n* = 13), mice received a single i.m. injection of Lenti-PROST-02 which led to full tumor eradication (Figures 6A and 6B). To mimic tumor relapse, cured mice were re-challenged s.c. with 3E9 cells in the opposite flank on day 68 after the first tumor engraftment, and maintained without any additional treatment (Figures 6A and 6B). A group of naive mice received the same 3E9 tumor cell suspension, as a positive control of tumor growth (*n* = 7). All naive control mice developed tumors, while all initially cured mice were protected from the tumor re-challenge and were still alive 200 days after the initial tumor engraftment (Figure 6B).

To awaken antigen-specific memory T cells without triggering primary T cells,^17^^,^^40^^,^^41^ on day 200 after the initial tumor transplantation, Lenti-PROST-02-treated cured mice (*n* = 6) or naive mice (*n* = 2) received an i.v. instillation of an adjuvant-free mixture of the immunogenic PSA #14 and PSA #40 peptides (Figure 6A). Two days later, splenocytes were stimulated *in vitro* with individual homologous peptides or an irrelevant peptide and studied by IFNγ ELISPOT (Figure 6C) and ICS and surface marker labeling (Figures 6D and 6E). PSA#14- or PSA#40-specific IFNγ-producing T splenocytes were detected in the cured mice after stimulation with homologous—but not an irrelevant control—peptides (Figures 6D–6F). The main subset of the responding CD8^+^ T cells in the cured mice had a CD44^+^ CD62L^−^ CD127^+^ KLRG1^−^ phenotype (Figures 6E–6G), a hallmark of memory precursor T cells, detectable at this advanced time point of 200 days after the beginning of the experiment.^12^

To assess whether T cells distinct from vaccine-elicited PSA-specific T cells contribute to the protective immunity observed upon tumor rechallenge, we subjected Lenti-PROST-02-treated mice, previously cured of 3E9 tumors, to a rechallenge with the parental EL4 cell line, which lacks PSA expression (Figure 6H). Unlike naive controls, which exhibited robust tumor outgrowth, cured mice resisted EL4 rechallenge (Figure 6I). This observation indicates that the initial PSA-targeted T cell response induced by Lenti-PROST-02 also favors triggering of a durable, antigen-diversified memory response, likely via epitope spreading.^42^ This process, wherein the initial immune response against a defined antigen (e.g., PSA) facilitates the priming of T cells against additional tumor-associated antigens, possibly released during therapy-induced tumor cell death, represents a critical hallmark of effective anti-tumor immunity and is strongly correlated with improved therapeutic outcomes.

In summary, a single i.m. injection of Lenti-PROST-02 elicits potent antitumor immunity, driven by prostate-specific antigen-targeted T cell responses. This intervention not only achieves complete tumor eradication but also fosters durable and broad immunological memory, which indicates its promise as a sustained and targeted immunotherapeutic strategy.

## Discussion

Lentiviral vectors efficiently transduce immature and non-dividing DCs.^13^^,^^14^^,^^43^ The genetic message delivered by non-integrative lentiviral vectors remains in the nuclei of the transduced DCs, without interaction with the host-cell chromosome. These vectors are scarcely inflammatory.^43^^,^^44^ Their transgene expression driven by β2m promoter enhances antigen presentation upon DC maturation and mobilization,^13^^,^^14^ making them ideal for immuno-oncotherapy. Here, Lenti-PROST-02 elicited high-quality, prostate-specific T cell responses, overcoming immunosuppressive tumor microenvironment in a virulent murine tumor model. Treatment induced polyfunctional CD8^+^ T cells targeting PAP/PSA epitopes and expanded CD44^+^ PD1^+^ TIM3^+^ KLRG1^+^ effector CD8^+^ TILs, reflecting sustained antitumor activity. In Lenti-PROST-02-treated mice, shrinking tumors exhibited a “cold-to-hot” inflammatory shift, indicating immune microenvironment remodeling. The primary antitumor mechanism involves vaccine-induced CD8^+^ T cells, while the immunostimulatory microenvironment transition, known to enhance immunotherapy efficacy,^39^ likely arises secondarily from effector T cell activity. We have also observed a “cold-to-hot” inflammatory switch in the TC1 model, which is the standard preclinical model of HPV-induced cancers.^17^^,^^18^ A single i.m. injection of the «Lenti-HPV-07» vector, which encodes detoxified HPV16/18 E6/E7 oncoproteins, triggers even more intense tumor microenvironment remodeling than the Lenti-PROST-02 vector does in 3E9 tumors. Similarly, the “Lenti-KRAS” vector, encoding the mutated KRAS^G12D^ antigen, induces significant intratumoral CD8^+^ T cell recruitment and an inflammatory shift in the MC38-KRAS^G12D^ model,^45^ though it is less pronounced than in the TC1 and 3E9 models. The intensity and breath of this “cold-to-hot” inflammatory shift appear to depend to the T cell immunogenicity of the antigen encoded by the lentiviral vector in the recipients. This includes the MHC alleles they harbor, as well as the tumor’s intrinsic immunological and microenvironmental properties.

We also observed that the initial, well-directed Lenti-PROST-02-induced T cell response lays the groundwork for a sustained, broad-spectrum memory immune response. This sets the stage for the subsequent recognition and response to a wider array of tumor-associated antigens, which are seemingly released following the efficient lysis of tumor cells. This reduces the likelihood of tumor escape and relapse. The establishment of this adaptive, antigen-diversified T cell immunity is a critical advancement in our understanding of how targeted immunotherapies based on robust immunization platforms can achieve long-lasting anti-tumor outcomes. The broadened immune response and secondary tumor antigens contributing to epitope spreading will be identified in future studies via combined transcriptomic/proteomic/MHC-I immunopeptidomic profiling of EL4 and 3E9 tumors, alongside TCR sequencing of CD8^+^ memory T cells. This will allow tracking of expanded T cell clones and defining the molecular mechanisms of epitope spreading in memory responses.

Our previous experimental data and comparative literature analyses consistently demonstrate the superior antitumor efficacy of lentiviral vectors over alternative vaccine platforms. When targeting the same antigen in identical preclinical tumor models, lentiviral vectors outperformed adjuvanted proteins, adenoviral, arenaviral, and poxviral vectors, as well as DNA- or LNP-mRNA-based vaccines.^12^^,^^13^^,^^19^^,^^46^ Additionally, lentiviral vectors offer distinct safety advantages due to their low inflammatory profile.^43^^,^^44^ They also achieve maximal effectiveness at relatively low doses (10^8^–10^9^ TU), whereas adenoviral vectors require much higher particle numbers (10^11^1012 infectious genome units; IGU) to reach comparable efficacy.^46^ The need for higher doses not only significantly increases the risk of adverse effects but also results in high costs and limitations in the scalability of GMP production.

Lenti-PROST-02 was engineered using HLA-I-restricted epitopes derived from PSA and PAP for human immunotherapy. Despite preclinical validation conducted exclusively in mice, the vector was designed to bypass the need for reformulation, enabling direct clinical translation. This was achieved by leveraging the cross-species immunogenicity of the PROST-02 polyantigen, which, though optimized for HLA-I, retained immunogenic regions functional in H-2^b^ mice (Table S2). Thus, the same construct validated preclinically remains clinically applicable without modification, streamlining development for human immuno-oncotherapy trials.

Human prostate tumors exhibit low immunogenicity and an immunosuppressive microenvironment,^7^ lacking high mutational burden, microsatellite instability, or mismatch repair defects to generate targetable neoantigens.^47^^,^^48^ Consequently, CTLA-4/PD-1/PD-L1 blockade shows limited efficacy in mCRPC.^11^ Therefore, promoting immune responses against antigens, actively produced by prostate tumor, with an expression profile severely restricted to this organ, can be promising. Advanced immunotherapeutic targets in clinical trials include (1) PAP-targeted DNA vaccines + anti-PD1 mAbs, (2) viral vectors encoding PSA/carcinoembryonic antigen/mucin-1 ± co-stimulatory molecules, (3) adenoviral Ad5 vectors encoding PSA/mucin-1/Brachyury antigens, (4) recombinant New York esophageal squamous cell carcinoma-1 (NY-ESO-1) + CpG ODNs, and (5) telomerase-derived peptides + granulocyte-macrophage colony stimulating factor (GM-CSF).^11^^,^^49^ PSMA-based immunotherapy carries autoimmune risk due to nervous system expression.

Sipuleucel-T, the only FDA-/EMA-approved antigen-based immunotherapy for asymptomatic or minimally symptomatic mCRPC, involves *ex vivo* incubation of autologous CD54^+^ antigen-presenting cells with a PAP-GM-CSF fusion protein, followed by i.v. reinfusion.^9^ GM-CSF directs uptake via DC receptors, and treatment requires three leukaphereses every two weeks. Clinically, it extended median survival by 4.1 months and reduced mortality risk by 22.5% versus placebo,^8^^,^^10^ validating antigen-specific immunotherapy in mCRPC. However, recombinant protein loading of DCs typically favors MHC-II over MHC-I presentation, raising questions about PAP cross-presentation efficiency and CD8^+^ T cell induction.^50^ Nevertheless, the relative success of Sipuleucel-T promoted development of simpler, targeted immunotherapies, avoiding *ex vivo* manipulation, with lower cost and risk.^11^^,^^49^ Lentiviral vectors demonstrate superior MHC-I antigen presentation and durable immunity compared to adjuvanted proteins, peptides, or alternative viral vectors, including adenoviral, arenaviral, poxviral, including modified vaccinia Ankara (MVA), and DNA- or mRNA-based platforms.^19^ Preclinical comparisons in HPV-induced TC1 cancer model revealed that only lentiviral vectors achieved complete tumor eradication with prolonged immune memory, suggesting broad applicability in immuno-oncotherapy. Their natural DC tropism enables *in vivo* transduction, eliminating the need for *ex vivo* manipulation (e.g., leukapheresis) while promoting endogenous MHC-I-mediated antigen presentation, enhancing therapeutic potential.

Immunotherapeutic strategies targeting CD8^+^ T cell responses in prostate cancer must address the critical challenge of HLA-I downregulation, that undermines T cell-mediated cytotoxicity. HLA-I downregulation occurs in ≈70% of primary prostate tumors, with complete loss detected in ≈34% of primary tumors and ≈80% of metastatic lesions.^30^^,^^31^ The loss of HLA-I is frequently linked to reversible epigenetic modifications, particularly DNA methylation and histone H3 tri-methylation, which reduce chromatin accessibility and transcriptional activity.^51^ However, these alterations can be counteracted through epigenetic modulators including DNA methyltransferase inhibitors and histone deacetylase inhibitors which effectively restore HLA-I surface expression. Histone deacetylase inhibitors upregulate HLA-I in prostate tumors by increasing β2m expression and have been proposed to be advantageously combined to T cell-based immunotherapies of prostate cancers.^31^ Most of the components of the antigen presentation machineries and surface HLA-I can also upregulate under the action of IFNγ.^52^ Therefore, immunotherapeutic approaches that enhance IFNγ signaling, such as tumor-localized IL12 immunotherapy,^53^ or IFNγ-inducing adjuvants like CpG oligodeoxynucleotides,^11^ can reverse HLA-I downregulation and promote an immune-permissive tumor microenvironment. These strategies can be advantageously combined with therapeutic vaccines, including Lenti-PROST-02. In summary, overcoming HLA-I downregulation is essential for the success of CD8^+^ T cell immunotherapies in prostate cancer. Epigenetic modulation, IFNγ-inducing adjuvants, and IL12-based immunotherapy represent promising approaches to restore MHC-I presentation, thereby potentiating the efficacy of T-cell-targeted treatments and improving patient responses in an otherwise immunosuppressive tumor context. Unlike some human prostate tumors, the 3E9 tumor does not exhibit MHC-I downregulation *in vivo.* Consequently, we could not evaluate the effects of combining Lenti-PROST-02 with MHC-I restoration therapies in this model. Future studies will explore combining Lenti-PROST-02 with therapeutic strategies to counteract MHC-I downregulation when an adapted preclinical tumor model is available.

It is important to note that preclinical murine models underestimate the efficacy of lentiviral vectors in primates due to species-specific transduction differences.^13^^,^^54^ This is supported by non-human primate studies and clinical trials,^55^ (NCT06319963) where doses of 10^8^–10^9^ TU/individual, similar to murine doses, demonstrated strong immunogenicity. This dose parity reflects the weak transduction efficiency of lentiviral vectors in murine cells relative to primate cells. A prospective Lenti-PROST-02 clinical trial will incorporate a dose-escalation phase to rigorously evaluate the safety, immunogenicity, and efficacy across multiple dose levels in humans.

A phase 1/2a trial (NCT06319963) using a lentiviral vector encoding HPV16/18 oncoproteins was initiated for HPV-associated oropharyngeal or cervical cancer, supported by preclinical murine efficacy.^17^^,^^18^ Prior clinical trials confirmed safety, tolerability, immunogenicity, and non-genotoxicity of lentiviral vectors (2011-006260-52).^15^^,^^16^ With growing clinical adoption, this approach is poised for broader human immunotherapy applications. We are currently extending the lentiviral vector-based immunotherapy to non-viral cancers, starting with prostate cancer.

## Materials and methods

### Lenti-PROST-02

Codon-optimized sequence of the designed PROST-02 antigen was synthesized by Genecust (Boynes, France) and inserted into the pFlap lentiviral plasmid, between the BamHI and XhoI sites, located between the human β2-microglobulin promoter and the mutated *atg* starting codon of the woodchuck post-transcriptional regulatory element and production of non-integrative Lenti-PROST-02 vector was performed as described.^12^^,^^17^ The integrase resulting from the packaging plasmid carries a missense D64V mutation in its catalytic triad which prevents the integration of viral DNA into the host chromosome.^13^

### Mice and immuno-oncotherapy

Six- to 12-week-old male C57BL/6JRj mice (Janvier, Le Genest Saint Isle, France) were housed in ventilated cages under specific pathogen-free conditions at the Institut Pasteur animal facilities. All methods were carried out in accordance with ARRIVE regulations (https://arriveguidelines.org) and in accordance with the European and French guidelines (Directive 86/609/CEE and Decree 87–848 of 19 October 1987) after approval of the protocol by the Institut Pasteur Safety, Animal Care and Use Committee delivered by the local ethics committee (CETEA #DAP180049, CETEA #DAP190130 and Ministry of High Education and Research APAFIS#16381–2018080217194542 v1, APAFIS# 20981–20190606164112731.

The EL4 cells (ATCC, TIB-39) were transduced by an integrative lentiviral vector encoding the full-length hPSA under the ubiquitin promoter.^56^^,^^57^ The transduced EL4 cells were cloned and screened for their potential to secrete hPSA, determined by hPSA DuoSet ELISA kit (Biotechne). The EL4::PSA “3E9” clone was selected for its capacity to secrete high amounts of hPSA. 3E9and EL4 cells were cultured in DMEM supplemented with 10% FBS, penicillin (100 U/mL), and streptomycin (100 μg/mL).

Mice were engrafted s.c. with 8 × 10^5^ 3E9 cells and were immunized i.m. with Lenti-PROST-02 in 50 μL. The tumors were measured 2 to 3 times a week using a digital caliper, in a blind manner. The tumor volume was calculated using the formula V = L × W^2^/2, where V is the volume, L the longest diameter, and W the shortest diameter. Mice were killed when tumors reached 1,500 mm^3^. Depletion using specific antibodies was conducted as described previously.^17^^,^^58^

### T cell assays

Fifteen-mer peptides spanning the PAP or PSA segments included in PROST-02 were synthetized by Genscript and were used at final concentration of 1 μg/mL for ELISPOT or 2–5 μg/mL for ICS. T cell assays were performed in RPMI1640 supplemented with 10% FBS, penicillin, streptomycin, 1 × 10^4^ M non-essential aa, 1% HEPES, 1 × 10^3^ M sodium pyruvate and 5 × 10^5^ M of β-mercapto-ethanol. ELISPOT and ICS on splenocytes were performed as described.^43^^,^^58^

### Cytometric analysis of tumor immune infiltrates

Tumors were digested by use of Collagenase and DNAseI and were homogenized in a GentleMacs as described.^58^ Tumor cell suspensions were filtered through 70 μm-pore filters, washed in PBS and stained as follows.

To study T cell activation status, cells were stained with a mixture of (1) eF450-anti-CD4 (RM4-5), APC-anti-CD8 (53–6.7), FITC-anti-CD44 (IM7), and PerCP-eF710-anti-KLRG1 (2F1) or (2) PerCp-Cy5.5-anti-CD4 (RM4-5), BV711-anti-CD8 (53–6.7), FITC-anti-PD1 (J43), and PE-Cy7-anti-TIM3 (RMT3-23). To study MHC-I expression on tumors, cells were stained with PE-anti-H-2K^b^ (REA1198) and PerCP-Cy5.5-anti-H-2D^b^ (KH95). All mixtures contained FcγII/III receptor blocking mAb (2.4G2) and near IR Live/Dead.

Tumor homogenates were enriched in lymphocytes on Ficoll and co-cultured in 24-well plates with syngeneic bone-marrow-derived DC pulsed with 10 μg/mL of various peptides for 6 h in the presence of anti-CD28 and anti-CD49d mAbs. During the last 3 h of incubation monensin and brefeldin A, each at 1 μg/mL, were added. Cells were then surface stained with a mixture of eF450-anti-CD4 (RM4-5), BV711-anti-CD8 (53–6.7), BV510-anti-PD1 (29F.1A12), PE-Dazzle-594-anti-KLRG1 (2F1), and PE-Cy7-anti-TIM3 (RMT3-23).

All mixtures contained FcγII/III receptor blocking mAb and near IR Live/Dead. Cells were permeabilized using the Cytofix/Cytoperm kit for ICS by use of APC-anti-IFNγ (XMG1.2), FITC-anti-TNFα (MP6-XT22), and PE-anti-IL2 (JES6-5H4) mAbs.

Cells were fixed with Cytofix before acquisition in an Attune NxT cytometer. The data were analyzed using FlowJo.

### Cytometric analysis of antigen-specific TILs

To determine activation/differentiation status of antigen-specific TILs, tumor homogenates, prepared as mentioned previously, were enriched in lymphocytes on Ficoll gradient and were co-cultured in 24-well plates with syngeneic bone-marrow-derived DC pulsed with 10 μg/mL of PAP #25, PSA #14 or a negative irrelevant peptide for 6 h in the presence of anti-CD28 and anti-CD49d mAbs. During the last 3 h of incubation monensin and brefeldin A, each at 1 μg/mL, were added to block cytokine secretion. Cells were then washed and surface stained with a mixture of eF450-anti-CD4 (RM4-5), BV711-anti-CD8 (53–6.7), BV510-anti-PD1 (29F.1A12), PE-Dazzle-594-anti-KLRG1 (2F1/KLRG1), PE-Cy7-anti-TIM3 (RMT3-23), PerCP-Cy5.5-anti-CXCR3 (S18001A), and BV605-anti-CCR6 (29-2L17). All mixtures contained FcγII/III receptor blocking anti-CD16/CD32 and Near IR Live/Dead. Cells were then permeabilized using the Cytofix/Cytoperm kit for ICS as detailed previously.

### Cytometric detection of memory CD8^+^ T splenocytes

Splenocytes from cured mice were cultured for 6 h in the presence of 2 μg/mL of various peptides and anti-CD28 and anti-CD49d mAbs. Monensin and brefeldin A were added as detailed above. Cells were surface stained with eF450-anti-CD4 (RM4-5), BV711-anti-CD8 (53–6.7), FITC-anti-CD44 (IM7), AF700-anti-CD62L (MEL-14), PE-Cy5-anti-CD127 (A7R34) and PE-Dazzle-594-anti-KLRG1 (2F1), FcγII/III receptor blocking mAb, and near IR Live/Dead. Cells were then permeabilized for ICS with APC-anti-IFNγ mAb.

### RT-qPCR

The tumor inflammatory mediators from treated mice were studied using the primer pairs listed in Table S3, and the comparative ΔΔ*C*_T_ method, as published.^17^^,^^58^

### Statistical analyses

Statistical analyses were performed using Prism GraphPad v.9.

## Data and code availability

The published article includes all datasets generated and analyzed during this study. All plasmids and lentiviral vectors generated in this study will be available under an MTA for research use, given a pending patent directed to Lenti-PROST-02 vaccination vector. Further information and requests for resources and reagents should be directed to and will be fulfilled by the corresponding author (laleh.majlessi@pasteur.fr).

## Acknowledgments

This work was supported by 10.13039/501100003762Institut Pasteur and TheraVectys. The authors are grateful to Dr. Vincenzo Di Bartolo (Institut Pasteur) for providing the EL4 tumor cell line.

## Author contributions

B.V., I.F., and S.C. performed most of the *in vivo* and *in vitro* experiments and formal analyses. F.M., A.N., and C.B. carried out the various stages of plasmid synthesis and lentiviral vector production and concentration. L.D. performed *in vivo* antitumor experiments and quantified human PSA in the circulation. P.A. and F.L.C. contributed to the antitumor *in vivo* experiments. F.N. initiated the tumor and immuno-oncotherapy model. K.N. contributed to the antigen design and epitope prediction. L.M. conceptualized the studies, supervised the project and wrote the manuscript, P.C. initiated and conceptualized the studies and funded the project.

## Declaration of interests

P.C. is the founder and CSO of TheraVectys. B.V., I.F., F.M., A.N., L.D., P.A., F.L.C., F.N., and K.N. are or have been employees of TheraVectys. L.M. had a consultancy activity for TheraVectys. B.V., I.F., F.M., L.D., P.A., F.L.C., L.M. and P.C. are inventors of a pending patent directed to the potential of Lenti-PROST-02 vaccination against prostate cancers.

## Supplemental information

Document S1. Figures S1–S6 and Tables S1–S3

Document S2. Article plus supplemental information
