# Supplementary material for: Eradication of pre-clinical prostate tumors by a lentiviral vector-based immunotherapy
Source: Mol Ther Oncol. 2026 Jul 18;34(3):201299. doi: 10.1016/j.omton.2026.201299 (PMC13471139; doi:10.1016/j.omton.2026.201299)
Supplement: Supplementary file 1 [file mmc1.pdf]

OMTON, Volume *34*

Supplemental information

Eradication of pre-clinical prostate tumors by a lentiviral vector-based immunotherapy

Benjamin Vesin, Ingrid Fert, Sylvain Ciret, Laëtitia Douguet, Fanny Moncoq, Amandine Noirat, Pierre Authié, Fabien Le Chevalier, Fabien Nevo, Catherine Blanc, Kirill Nemirov, Laleh Majlessi, and Pierre Charneau

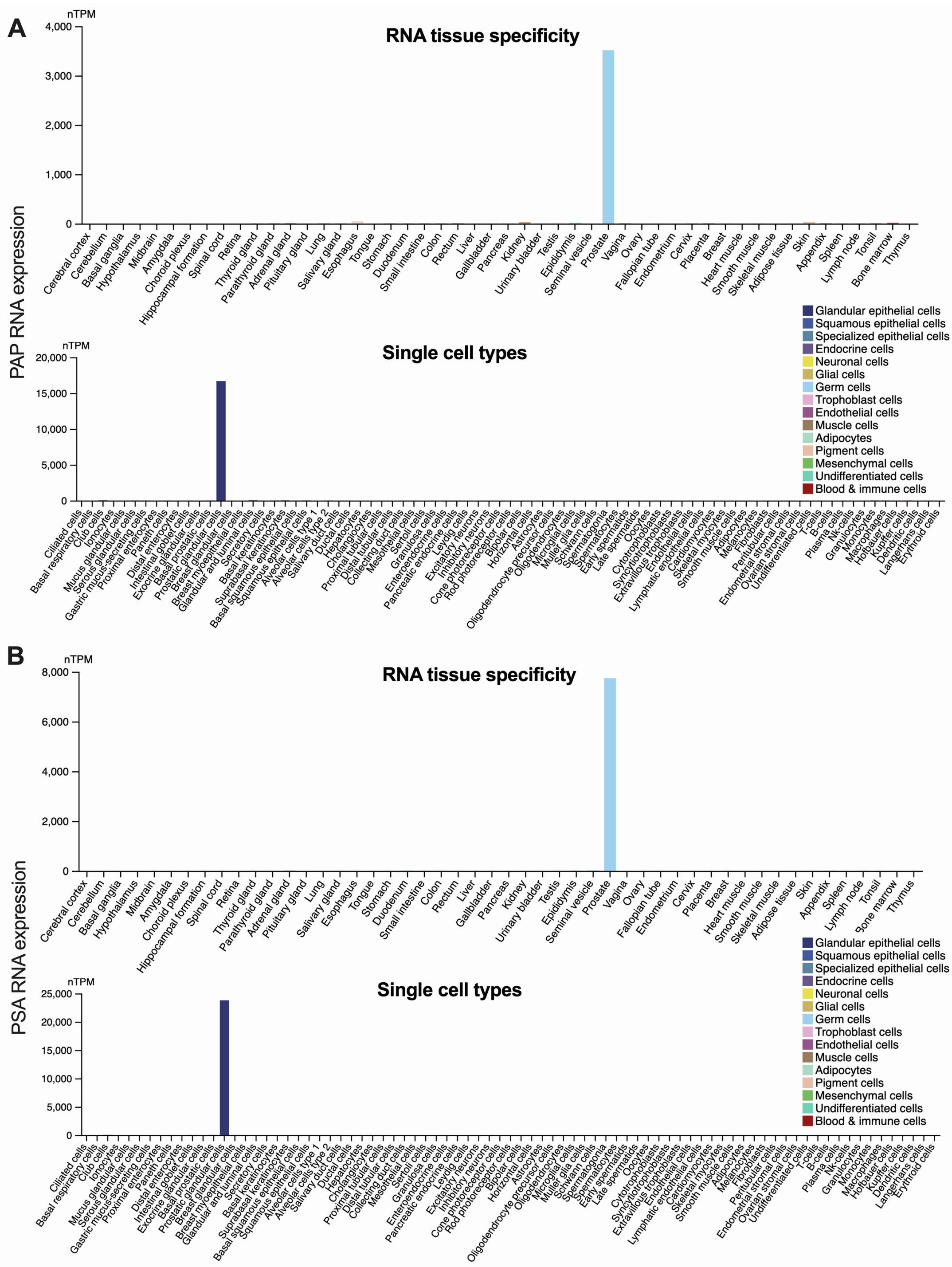

Figure S1. PAP and PSA mRNA expression in human organs. (A-B, top) Consensus specificity of PAP (https://www.proteinatlas.org/ENSG00000014257-ACP3/tissue#rna_expression) and PSA (https://www.proteinatlas.org/ENSG00000142515-KLK3/tissue) mRNA expression in human organs. **(A-B, bottom)** Specificity of PAP (https://www.proteinatlas.org/ENSG00000014257-ACP3/single+cell+type) and PSA (https://www.proteinatlas.org/ENSG00000142515-KLK3/single+cell+type) mRNA expression in cell types.[1,2]

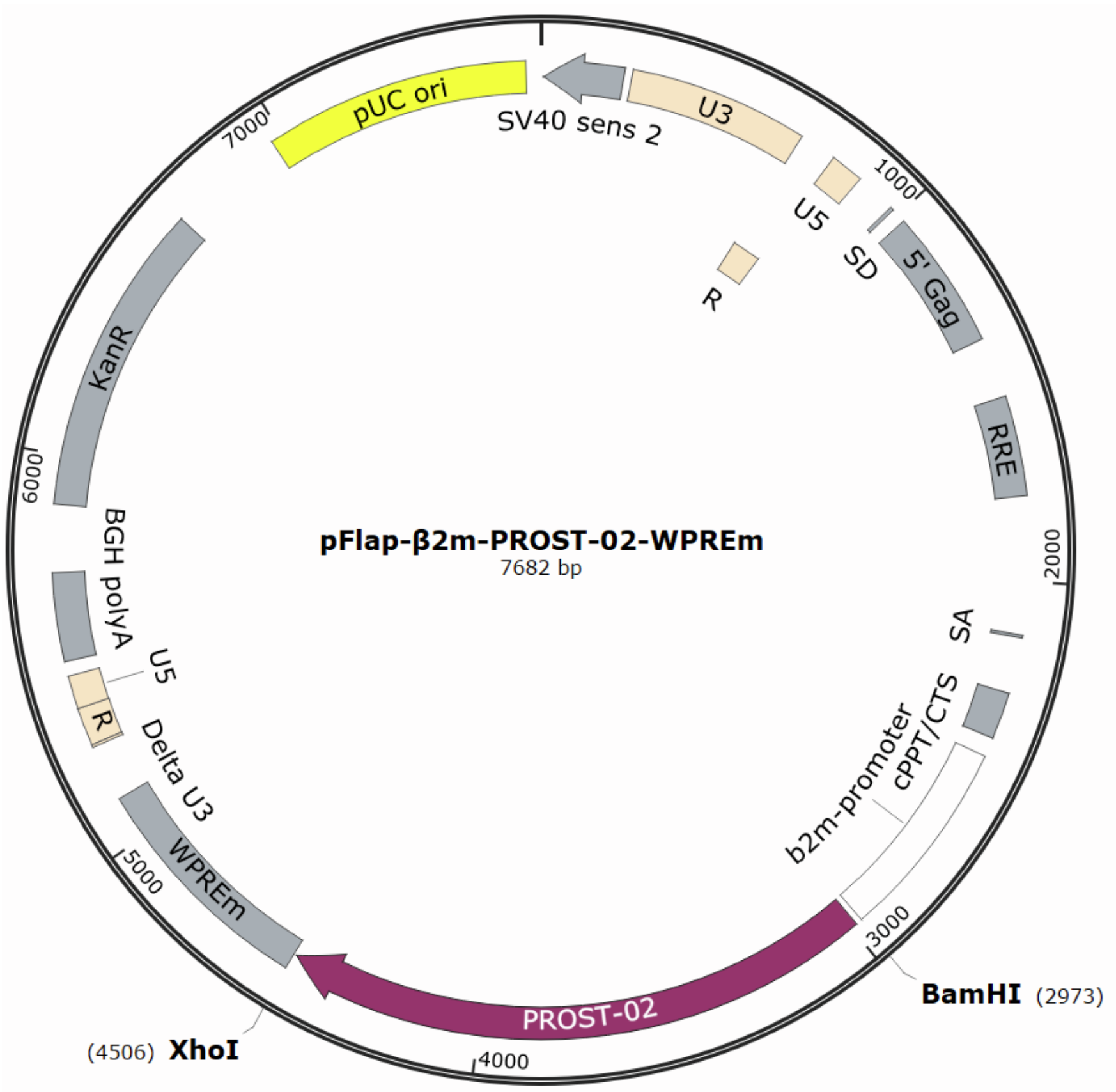

PROST-02 nucleotide sequence (1521 bp)

ATGAGGGCCGCCCCTTTGTTGTTGGCTAGGGCCGCCTCCCTGTCCCTGGGCTTCCTGTTCCTGCTGTTCTTCTGGCTGGA
CCGGTCCGTGTTGGCCAAGGAGCTGAAGTTCGTGACCCTGGTGTTTCGGCATGGCCACTACGAGTTGGGAGAATATATCC
GCAAGAGGTACCGCAAGTTCCTGAACGAGTCTTACAAGCACGAGCAGGTTTACATCAGGTCCACCGACGTGGACAGGACT
CTCATGTCCGCTATGACCAACCTGGCCGCACTGTTCCCCCCCGAAGGCGTGTCCATCTGGAACCCCATCCTCCTGTGGCA
GCCCATCCCCGTGCACACCGTGCCCCTGAGCGAGGACCAGCTGCTGTACCTGCCCTTCCGCAATTGCCCCCGTTTCCAGG
AGCTGAGACTCCACCCCTACAAGGACTTCATTGCCACCCTCGGCAAGTTGTCCGGCCTGCACGGCCAGGATTTGTTCGGC
ATTTGGTCCAAAGTCTATGACCCCCTCTACTGCGAATCCGTGCACAACTTCACCCTCCCCTCCTGGGCCACCGAAGACAC
CATGACCAAGCTGAGGGAGCTGTCCGAGCTGAGTTTGCTGTCCCTGTACGGCATCCACAAACAGAAGGAGAAATCCCATA
TGAAAAGGGCCACCCAGATCCCCTCCTACAAGAAACTGATCATGTACTCCGCCCACGACACCACAGTCTCCGGCCTCCAG
ATGGCCCTCGACGTCTACAACGGACTGCTGCCCCCCTACGCCTCCTGTCACCTCACCGAACTCTACTTCGAAAAGGGCGA
ATACTTCGTGGAAATGTACTACAGGAACGAAACCCAGCACGAACCCTATCCCCTCATGCTCCCCGGCCCCGACGACTGGG
TGCCTGTTGTCTTCCTGACTTTGTCCGTGACCTGGATCGGCGCTGCCCCTCTGATCTTGTCCCGGATTGTGGCGGCTGG
GAGTGCGAAAAACACTCCCAGAGGGGCCGGGCTGTGTGTGGCGGTGTGCTGGTGCATCCTCAGTGGGTGCTGACTGCTGC
TCATTGCATTAGGAACAAGAGTGTGATCTTGCTGGGGAGGCACTCCTTGTTCCACCCCGAGGATACTGGTCAGGTGTTTC
AGGTGTCCCATTCCTTCCCCCACCCCCTCTACGACATGTCCCTGCTGAAGAACAGATTCTTGCGCCCCGGCGACGACTCC
TCCCACGACTTGATGCTGCTGCGGCTCGGCCCCGACGATATGGACCTCCCCACCCAGGAGCCCGCCCTGGGAACAACCTG
CTACGCCTCCGGCTGGGGCTCCATCGAGCCCGAAGAGTTCCTCACCCCCAAGAAACTGCAGTGCGTTGACCTGCACGTGA
TTTCCAACGACGTGTGCGCCCAGGTGCACCCCCAGAAGGTGACCAAGTTCATGCTGTGCGCCGGCAGGTGGACAGGCGGC
TCTGAACCCTGCGCCCTCCCCGAAAGACCCTCCCTGTACACCAAAGTGGTGCACTATAGGAAGTGGATCAAGGACTGATA
A

Figure S2. Map of the plasmid encoding PROST-02 antigenic fusion protein. The codon-optimized cDNA sequence, encoding PROST-02 (PAP-PSA), indicated in Fig. 1 was inserted under the β2m promoter in a lentiviral pFLAP backbone plasmid. The PROST-02 nucleic acid sequence is indicated, bp = base pair.

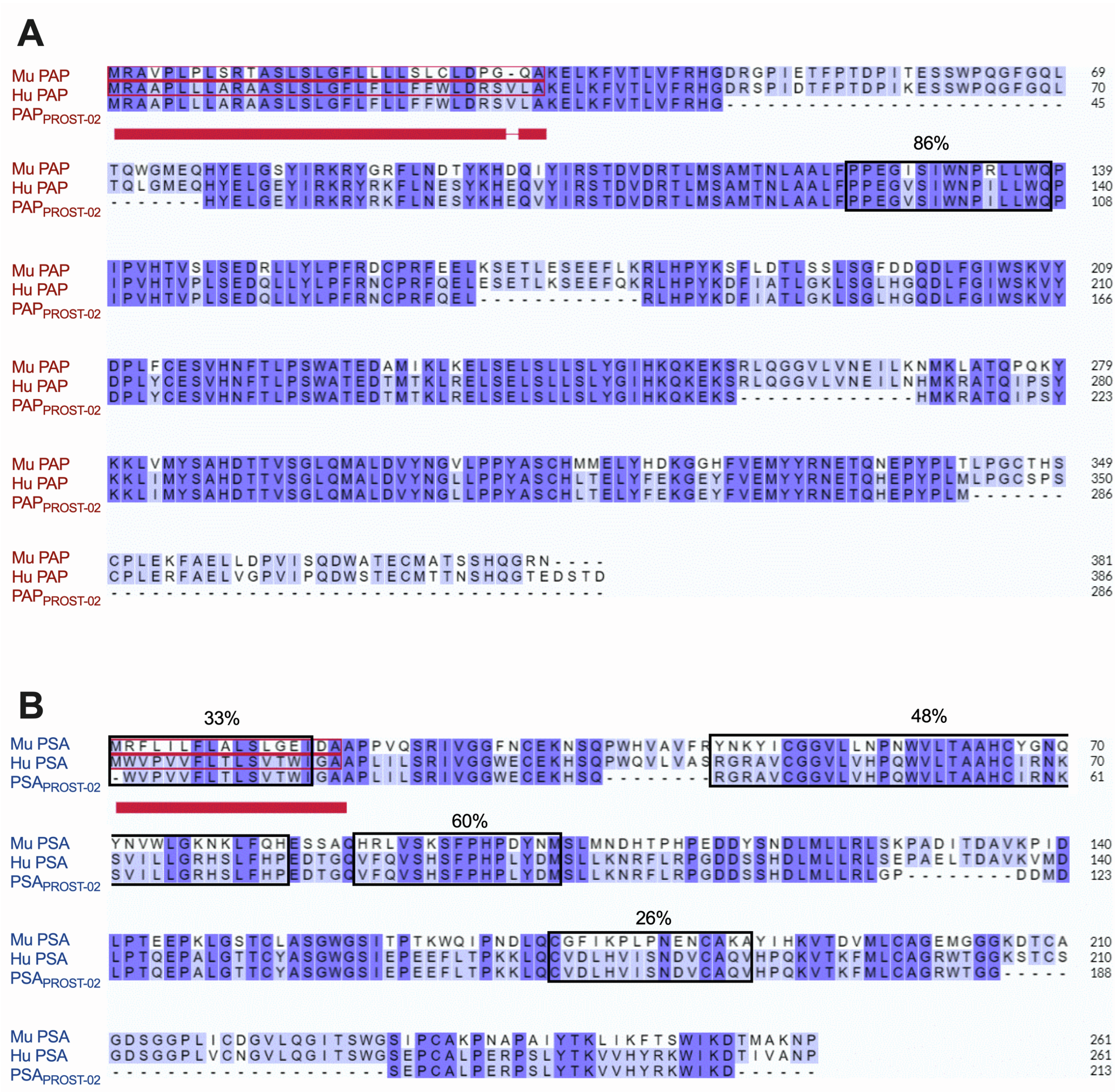

Figure S3. Sequence comparison of murine, human and Lenti-PROST-02-encoded PAP and PSA antigens. (A) Aa sequence alignment of PAP, murine (first line, "Mu") (Accession no Q8CE08), human (second line, "Hu") (Accession no P15309), and encoded by Lenti-PROST-02 (third line). **(B)** Aa sequence alignment of PSA, murine (first line, "Mu") (Accession no Q61759), human (second line, "Hu") (Accession no P07288), and encoded by Lenti-PROST-02 (third line). Immunogenic regions identified by epitope mapping in C57BL/6 ($H\text{-}2^b$) mice (Fig. 2B) are outlined in black. Aa highlighted in dark or pale purple are identical for the three or two sequences, respectively. For each immunogenic region, the percentages correspond to the sequence identity between the murine ortholog and the sequence encoded by Lenti-PROST-02. The N-terminal red lines indicate the secretory signal sequence. Analysis was performed using Uniprot Blast (https://www.uniprot.org/blast).

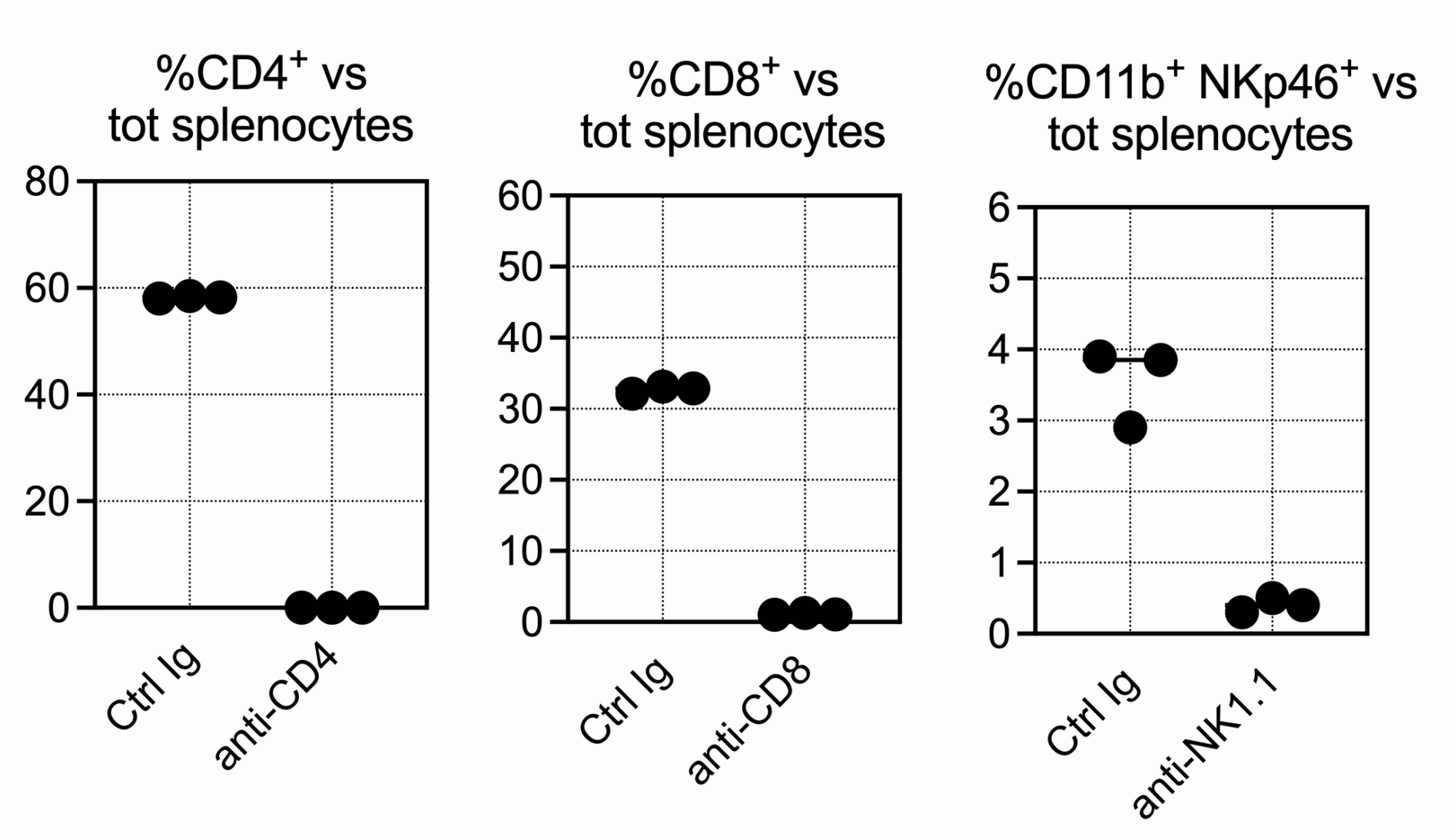

Figure S4. Efficacy of anti-CD4, anti-CD8 or anti-NK1.1 depletion in vivo. Tumor-free C57BL/6 mice (*n* = 3/group) received repeated i.p. injections of 200 µg anti-CD4 (clone GK1.5), anti-CD8 (clone H35.17.2), anti-NK1.1 (PK136) or a control Ig isotype. On day 3 after the last injection, the spleens from individual mice were assessed by anti-CD3, anti-CD4, and anti-CD8 or anti-CD11b and anti-NKp46 mAb staining and cytometry analysis to evaluate the efficacy of cell subset depletion. The results from 3 individual mice/group are shown.

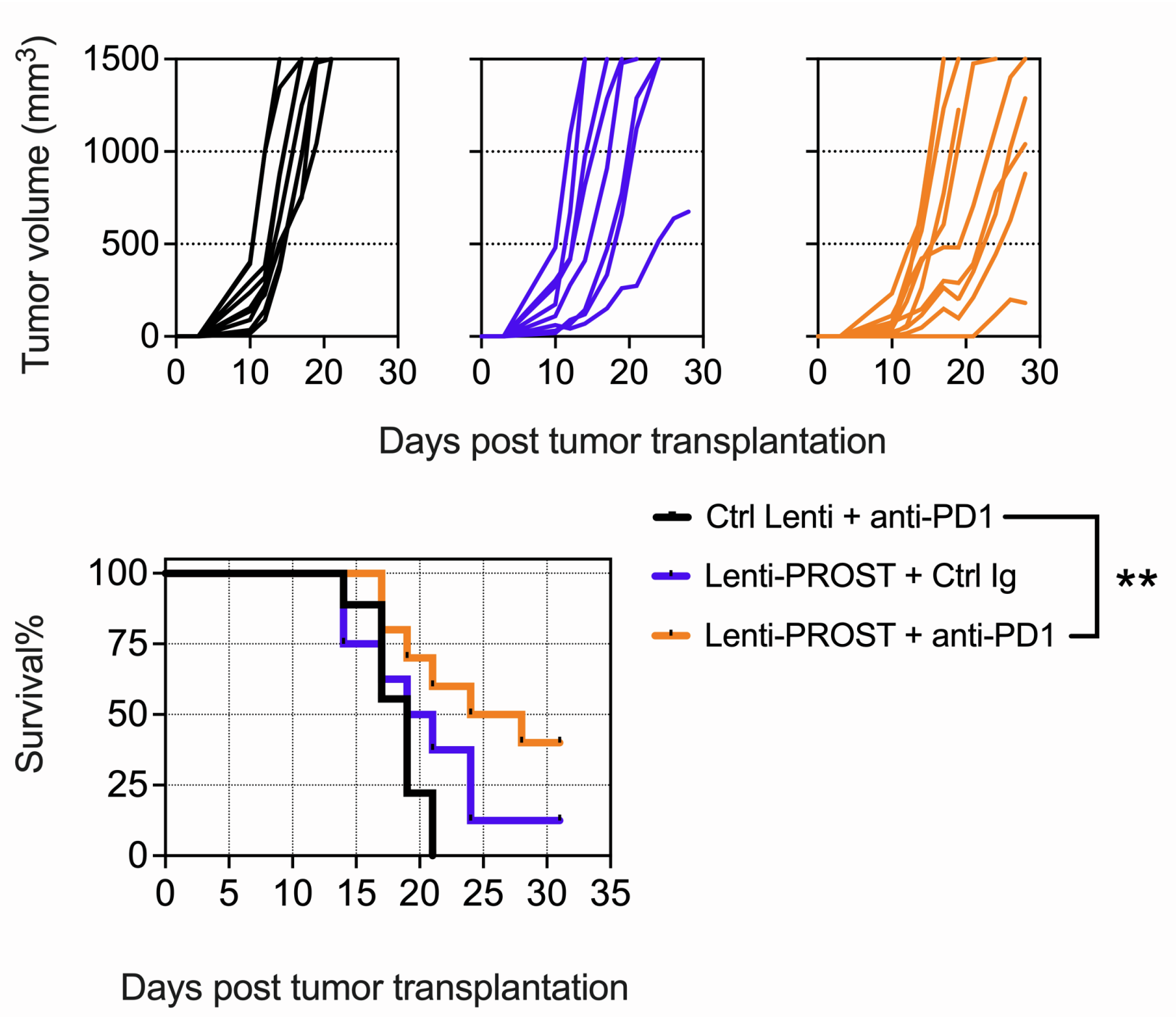

Figure S5. Lentiviral vector-based immunotherapy of tumors expressing human PAP. C57BL/6 male mice (n = 7-9/group) were engrafted s.c. on the flank with 2×10^5 of the MC38-PAP tumor cells. On day 6 post tumor engraftment, mice were randomized by tumor size and injected i.m. with the vaccine or Ctrl Lenti. Starting on day 10, 4 days after vaccination, mice received intraperitoneal injections of 200 µg/mouse/injection of anti-PD1 mAb (clone RMP1-14) or Ctrl Ig every 2 days. Tumor volume versus time (top). Survival of animals (bottom). Statistical significance was determined by log-rank Mantel-Cox test (**$p < 0.01$). Mice were sacrificed when tumors reached 1500 mm^3.

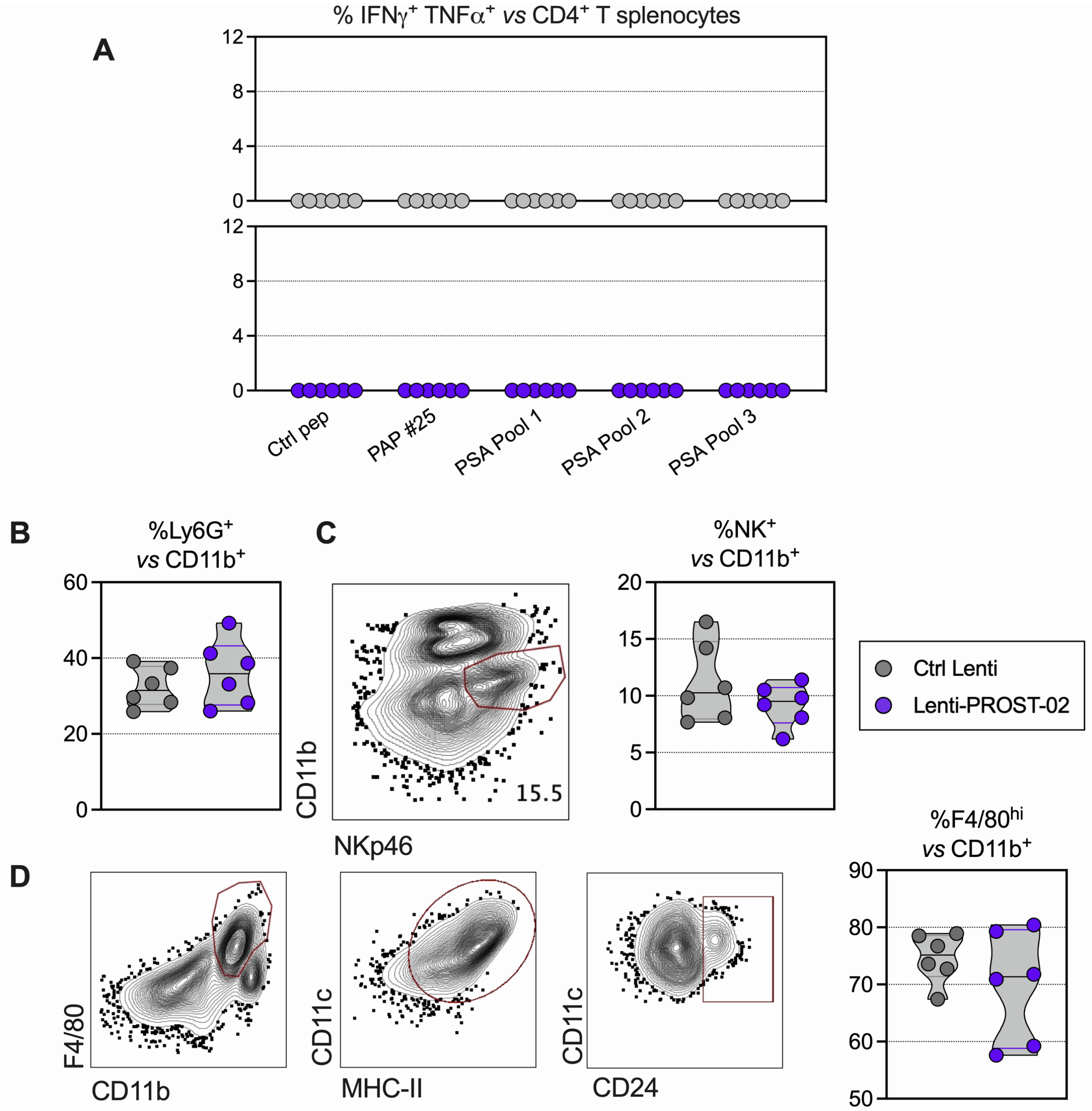

Figure S6. Additional immunological features of Ctrl Lenti- or Lenti-PROST-02-treated mice. **(A)** Absence of CD4$^+$ T splenocyte responses in 3E9-bearing mice treated with Ctrl Lenti or Lenti-PROST-02, as determined by ICS. **(B-D)** Cytometric analysis of innate immune cells in the 3E9 tumors of Ctrl Lenti- or Lenti-PROST-02-treated mice. The tumors were from the mice described in the Figure 4. Frequencies of granulocytes/neutrophils **(B)**, as cytometrically defined in the Figure 3B, **(C)** NK cells, and **(D)** F4/80^{hi} cells in the tumors of Ctrl Lenti- or Lenti-PROST-02-treated mice, on day 9 post treatment. Note that the F4/80^{hi} CD11b^{int} cells were CD11c$^+$ and MHC-II$^+$ and contained a subset of CD24$^+$ cells. Differences between the two groups were not statistically significant, determined by two-tailed Mann-Whitney test.

Table S1. HLA-1 alleles considered for epitope prediction by IEDB on human PSA and PAP sequences.

HLA-A	HLA-B
HLA-A*01:01	HLA-B*07:02
HLA-A*02:01	HLA-B*08:01
HLA-A*02:03	HLA-B*15:01
HLA-A*02:06	HLA-B*35:01
HLA-A*03:01	HLA-B*40:01
HLA-A*11:01	HLA-B*44:02
HLA-A*23:01	HLA-B*44:03
HLA-A*24:02	HLA-B*51:01
HLA-A*26:01	HLA-B*53:01
HLA-A*30:01	HLA-B*57:01
HLA-A*30:02	HLA-B*58:01
HLA-A*31:01	
HLA-A*32:01	
HLA-A*33:01	
HLA-A*68:01	
HLA-A*68:02	

These 27 HLA-1 alleles considered account for 97% of the HLA-A and HLA-B allelic variants in most human ethnicities.[3,4]

Table S2. Features of PAP and PSA epitopes mapped to PROST-02 antigen. Characteristics of PAP- and PSA-derived T-cell epitopes mapped to the human PROST-02 antigen in the murine H-2^b haplotype (Figure 2B). The human and murine sequences of the regions containing these epitopes were compared to visualize aa sequence identity. The fine epitope regions of the human sequences and their orthologous murine regions are highlighted in gray. Predicted binding scores of these sequences to H-$2K^b$ and H-$2D^b$, as predicted by SYFPEITHI (http://www.syfpeithi.de), are shown. The anchor residues are indicated in bold.

Prostate antigen	Pep	aa sequence	Identity	H-$2K^b$ epitope	H-$2K^b$ score*	H-$2D^b$ epitope	H-$2D^b$ score*
Hu PSA<br>Mu PSA	PAP #25	PPEGVSIWNPILLWQ<br>PPEGISIWNPRLLWQ	7/9	VSIW**N**PIL**L**	26	SIWN**P**IL**L**	13
Hu PSA<br>Mu PSA	PSA #1	MWVPVVFLTLSVTWI<br>MRFLILFLALSLGEI	3/9	WVPV**V**FLT**L**	17	VPVV**FL**T**L**	21
Hu PSA<br>Mu PSA	PSA #10	RGRAVCGGVLVHPQ<br>YNKYICGGVLLNPN	5/9	GRAV**C**GGV**L**	12	RAVC**GG**V**L**	12
Hu PSA<br>Mu PSA	PSA #14	LTAAHCIRNKSVILL<br>LTAAHCYGNQYNVWL	4/11	HCIR**N**KSV**I**	24	RNKS**VI**L**L**	13
Hu PSA<br>Mu PSA	PSA #16	NKSVILLGRHSLFHP<br>NQYNVWLGKNKLFQH	3/9	VILL**G**RHS**L**	13	ILLG**R**HS**L**	11
Hu PSA<br>Mu PSA	PSA #21	VFQVSHSFPHPLYDM<br>HRLVSKSFPHPDYNM	7/9	SFPH**P**LYD**M**	16	VSHS**F**PH**P**	11
Hu PSA<br>Mu PSA	PSA #40	CVDLHVISNDVCAQV<br>CGFIKPLPNENCAKA	3/11	SNDV**C**AQ**V**	9	HVIS**N**DVC**A**	13

*The score is defined by the probability for an aa sequence of being processed and presented by MHC molecules.

Table S3. Sequences of primers used in qRT-PCR.

Gene	cDNA accession number	Forward sequence (5’ to 3’)	Reverse sequence (5’ to 3’)	Amplicon (bp)
SDHA	NM_023281.1	TGCCCTGAGCATTGCAGAAT	TGCAATACACTCCCCACACG	198
IFNα	NM_010502.2	GGATGTGACCTTCCTCAGACTC	ACCTTCTCCTGCGGGAATCCAA	131
IFNγ	NM_008337.4	TCAAGTGGCATAGATGTGGAAGAA	TGGCTCTGCAGGATTTTCATG	92
TNFα	NM_013693	CATCTTCTCAAAATTCGAGTGACAA	TGGGAGTAGACAAGGTACAACCC	175
TGFβ	NM_011577.2	TGACGTCACTGGAGTTGTACGG	GGTTCATGTCATGGATGGTGC	170
IL-1β	NM_008361.4	TGCCACCTTTTGACAGTGATG	AAGGTCCACGGGAAAGACAC	220
IL-2	NM_008366.3	CCTGAGCAGGATGGAGAATTACA	TCCAGAACATGCCGCAGAG	141
IL-4	NM_021283.2	GAGCTCGTCTGTAGGGCTTC	TGCTCTTTAGGCTTTCCAGG	205
IL-5	NM_010558.1	GATGAGGCTTCCTGTCCCTACT	TGACAGGTTTTGGAATAGCATTTCC	130
IL-6	NM_031168.2	CTGCAAGTGCATCATCGTTGTTC	TACCACTTCACAAGTCGGAGGC	116
IL-10	NM_010548.2	ATGCTGCCTGCTCTTACTGAC	TCCAGCTGGTCCTTTGTTTGA	165
IL-12p40	NM_001303244.1	GGAAGCACGGCAGCAGAATA	AACTTGAGGGAGAAGTAGGAATGG	180
IL-18	NM_008360.2	GACAGCCTGTGTTCGAGGATATG	TGTTCTTACAGGAGAGGGTAGAC	159
IL-33	NM_001164724.2	CTACTGCATGAGACTCCGTTCTG	AGAATCCCGTGGATAGGCAGAG	136
CCL2	NM_011333.3	AGGTCCCTGTCATGCTTCTG	TCTGGACCCATTCCTTCTTG	249
CCL3	NM_011337.2	CCTCTGTCAC CTGCTCAACA	GATGAATTGGCGTGGAATCT	163
CCL5	NM_013653.3	GTGCCCACGTCAAGGAGTAT	GGGAAGCTATACAGGGTCA	185
CCL19	NM_011888.4	CTGCCTCAGATTATCTGCCAT	AGGTAGCGGAAGGCTTTCAC	177
CCL21	NM_002989.4	AAGGCAGTGATGGAGGGG	CGGGGTAAGAACAGGATTG	136
CXCL5	NM_009141.3	GCGTTGTGTTTGCTTAACCG	GAACACTGGCCGTTCTTTCC	242
CXCL9	NM_008599.4	AAAATT TCATCACGCCCTTG	TCTCCAGCTTGGTGAGGTCT	207
CXCL10	NM_021274.2	TGCCGTCATTTTCTGCCTCA	AGGCTCGCAGGGATGATTTC	150

References

1. Karlsson, M., Zhang, C., Mear, L., Zhong, W., Digre, A., Katona, B., Sjostedt, E., Butler, L., Odeberg, J., Dusart, P., et al. (2021). A single-cell type transcriptomics map of human tissues. Sci Adv *7*. 10.1126/sciadv.abh2169.
2. Uhlen, M., Fagerberg, L., Hallstrom, B.M., Lindskog, C., Oksvold, P., Mardinoglu, A., Sivertsson, A., Kampf, C., Sjostedt, E., Asplund, A., et al. (2015). Proteomics. Tissue-based map of the human proteome. Science *347*, 1260419. 10.1126/science.1260419.
3. Sette, A., and Sidney, J. (1999). Nine major HLA class I supertypes account for the vast preponderance of HLA-A and -B polymorphism. Immunogenetics *50*, 201-212. 10.1007/s002510050594.
4. Weiskopf, D., Angelo, M.A., de Azeredo, E.L., Sidney, J., Greenbaum, J.A., Fernando, A.N., Broadwater, A., Kolla, R.V., De Silva, A.D., de Silva, A.M., et al. (2013). Comprehensive analysis of dengue virus-specific responses supports an HLA-linked protective role for CD8+ T cells. Proc Natl Acad Sci U S A *110*, E2046-2053. 10.1073/pnas.1305227110.
