# Supplementary material for: Eradication of pre-clinical prostate tumors by a lentiviral vector-based immunotherapy
Source: Mol Ther Oncol. 2026 Jul 18;34(3):201299. doi: 10.1016/j.omton.2026.201299 (PMC13471139; doi:10.1016/j.omton.2026.201299)
Supplement: Supplementary file 2 [file mmc2.pdf]

Molecular Therapy
Oncology
Original Article

Eradication of pre-clinical prostate tumors by a lentiviral vector-based immunotherapy

Benjamin Vesin,[1,2,6] Ingrid Fert,[1,2,6] Sylvain Ciret,[1,5,6] Laëtitia Douguet,[1,2] Fanny Moncoq,[1,2] Amandine Noirat,[1,2] Pierre Authié,[1,3] Fabien Le Chevalier,[1,4] Fabien Nevo,[1] Catherine Blanc,[1,5] Kirill Nemirov,[1,2] Laleh Majlessi,[1,5,7] and Pierre Charneau[1,5,7]

[1]Pasteur-TheraVectys Joint Lab, Institut Pasteur, Université de Paris, Virology Department, 28 Rue Du Dr. Roux, 75015 Paris, France

Prostate cancer kills ≈ 350,000 yearly. While localized cases have 99% five-year survival, metastatic prostate cancer is hard to treat, with few options for tumors resistant to androgen deprivation. To leverage the immune system to fight prostate cancer, we developed a non-integrative lentiviral vector, namely "Lenti-PROST-02", which encodes clusters of T cell immunodominant regions of human prostatic acid phosphatase and prostate-specific antigen. Immunotherapy with Lenti-PROST-02 in a preclinical virulent prostate tumor model resulted in complete tumor eradication in vast majority of the treated animals. This antitumor effect was concomitant with induction of poly-functional CD8$^+$ T splenocyte effectors against numerous T cell epitopes of the antigens encoded by Lenti-PROST-02, increased proportions of tumor-infiltrating CD8$^+$ T cells with activated/differentiated/effector phenotype and a "cold-to-hot" inflammatory switch of the tumor microenvironment. Immunity induced by Lenti-PROST-02 was long-lasting and prevented tumor relapse. It was characterized by the persistence of CD44$^+$ CD62L$^-$ CD127$^+$ KLRG1$^-$ CD8$^+$ memory T cells in secondary lymphoid organs, as well as an antigen-diversified memory response. Therefore, Lenti-PROST-02 therapeutic vaccine is a promising approach for prostate immuno-oncotherapy.

INTRODUCTION

Prostate cancer is the second most common cancer in men after lung cancer, representing 15% of new diagnoses in high-income countries and causing over 350,000 annual deaths.[1] At diagnosis, ∼80% of cases remain prostate confined, 15% show locoregional spread, and 5% present distant metastases.[2] Localized disease has a favorable prognosis, with five-year survival rates nearing 99% post-treatment.[3] However, standard therapies—surgery, radiotherapy, or hormone deprivation—often impair quality of life. Metastatic progression, typically to lymph nodes, bone, or viscera, severely limits curative options, reducing treatment to palliative care. Advanced disease initially responds to androgen-deprivation therapy, defining metastatic castration-sensitive prostate cancer (mCSPC), where survival drops to ∼30% at five years.[4] Inevitably, resistance to androgen-deprivation therapy develops, marking the transition to metastatic castration-resistant prostate cancer (mCRPC),[5] a stage with few therapeutic options and poor outcomes.[6]

Immunotherapies targeting cytotoxic T-lymphocyte antigen 4 (CTLA-4), and/or programmed death-1 (PD1) or PD1 ligand (PDL1) have revolutionized immuno-oncotherapy but show limited efficacy in prostate cancer due to its "cold," lymphocyte-sparse and immunosuppressive microenvironment.[7] The FDA/EMA-approved "Sipuleucel-T", an autologous dendritic cell (DC)-based vaccine,[8–10] provided more encouraging results than immune checkpoint inhibitors, but still induced only partial clinical benefits, motivating development of more efficient T cell-based therapies.[11]

Non-integrative lentiviral vectors are effective in inducing long-lasting, high-quality T cell responses,[12–14] with proven safety in clinical trials (2011-006260-52).[15,16] Recent preclinical studies demonstrated their efficacy in human papilloma virus (HPV)-driven cancers, achieving 100% tumor eradication and long-term immunity.[17,18] A cross-sectional comparative review of the most relevant immuno-oncotherapy strategies, evaluated in the same HPV-induced murine cancer model, established that certain adjuvanted proteins and viral vectors showed the most robust antitumor effects. Among them, the lentiviral vectors were the only strategy resulting in full tumor eradication in 100% of animals with the longest-lasting immune memory able to prevent tumor relapse.[19] This success led to a phase 1/2a clinical trial for HPV-related cancers (NCT06319963) and prompted evaluation of lentiviral vector platform efficacy in prostate cancer, a non-viral malignancy.

Received 9 March 2026; accepted 16 July 2026;
https://doi.org/10.1016/j.omton.2026.201299.

[2]Present address: TheraVectys, 5 rue Henri Desbrueres, 91000 Evry-Courcouronnes, France

[3]Present address: Institut Imagine, Inserm UMR 1163, Université Paris Cité, 24 Boulevard du Montparnasse, 75015 Paris, France

[4]Present address: Virus sensing and signaling unit, Institut Pasteur, Université de Paris, Virology Department, 28 rue du Dr. Roux, 75015 Paris, France

[5]Present address: Virology Department, 28 rue du Dr. Roux, 75015 Paris, France

[6]These authors contributed equally

[7]Senior author

Correspondence: Laleh Majlessi, Pasteur-TheraVectys Joint Lab, Institut Pasteur, Université de Paris, Virology Department, 28 Rue Du Dr. Roux, 75015 Paris, France.
E-mail: laleh.majlessi@pasteur.fr

Published by Elsevier Inc. on behalf of The American Society of Gene and Cell Therapy.
This is an open access article under the CC BY license (http://creativecommons.org/licenses/by/4.0/).

Based on their overexpression and enrichment in prostate tumors, the most pertinent targeted antigens in these cancers include (1) prostatic acid phosphatase (PAP), (2) prostate-specific antigen (PSA), (3) prostate-specific membrane antigen (PSMA), and (4) prostate stem cell antigen (PSCA).[11] Unlike PSMA and PSCA, which exhibit off-target expression in non-prostatic tissues (e.g., PSMA in brain and PSCA in the bladder, kidney, stomach, and pancreas), PAP and PSA demonstrate far greater prostate specificity, minimizing the risk of off-target immune responses.[20–22] We thus selected PAP and PSA to include in a lentiviral vector-based immunotherapy. The inclusion of two prostate antigens mitigates the probability of tumor escape linked to a possible loss of expression of a single targeted antigen. PAP glycoprotein is expressed on >95% of prostate cancer cells, and is included in Sipuleucel-T. PSA is a neutral serine protease, a serum marker followed in men to screen the risk of developing prostate cancer or to monitor the disease progression or recurrence after initial diagnosis.[20] We generated a non-integrative lentiviral vector, namely "Lenti-PROST-02", which encodes clusters of predicted or previously identified T cell epitopes of human PAP and PSA, under the transcriptional control of the human β2 microglobulin promoter (β2m).[12] We have previously demonstrated that following intramuscular (i.m.) administration of lentiviral vectors encoding a reporter antigen under β2m promoter, multiple immune cell populations in draining lymph nodes become transduced within 5 days. The highest transduction efficiency is observed *in vivo,* at 2%–5% antigen-positive cells in macrophages and DC subsets (including conventional $CD8^+$ and $CD8^-$ DCs and plasmacytoid DCs), while T and B cells exhibit minimal (<1%) transduction. Upon immunization with a lentiviral vector encoding a model antigen under β2m promoter, all DC subsets efficiently prime antigen-specific $CD8^+$ T cells, but macrophages, B cells, and T cells do not. These results highlight DCs as the primary antigen-presenting cells responsible for initiating $CD8^+$ T cell immunity following i.m. delivery of lentiviral vector using the β2m promoter.[12]

In a preclinical virulent prostate cancer model set up here, Lenti-PROST-02 elicited robust $CD8^+$ T cell responses, inducing complete tumor regression in the great majority of treated mice. Critically, it established long-term immune memory, preventing relapse upon tumor re-challenge. Mechanistically, Lenti-PROST-02 therapy reprogrammed the tumor microenvironment by increasing $CD8^+$ tumor-infiltrating lymphocytes (TILs) with effector phenotypes, shifting "cold-to-hot" inflammation, and sustaining memory precursor T cells in the spleen. These data validate lentiviral vector-based immunotherapy as a promising strategy for prostate cancer, warranting clinical translation.

RESULTS

PROST-02 antigen design

Expression of the human PSA and PAP are highly specific to prostate glandular epithelial cells (Figures S1A and S1B) and increases with prostate cancer progression,[23,24] making them relevant antigens for cancer immunotherapy, without entailing risks of off-tumor (multi)-organ autoimmunity. To select the T cell immunogenic regions of PAP (Acc# P15309) and PSA (Acc# P07288) (Figure 1A), presentable by molecules of human leukocyte antigen of class-I (HLA-I), we used SYFPEITHI (http://www.syfpeithi.de), IEDB (https://www.iedb.org), and netCTLpan (https://services.healthtech.dtu.dk/services/NetCTLpan-1.1/) epitope prediction tools. The advantage of NetCTLpan is to provide a combined prediction score by integrating (1) strength of peptide-MHC-I binding, (2) efficacy of proteasomal C-terminal cleavage, and (3) efficacy of peptide transport by TAP (transporter associated with antigen processing). Initially, 9 amino acid (aa)-long peptides that could be presented by 6 common HLA alleles, i.e., HLA-A*01:01, HLA-A*02:01, HLA-A*03:01, HLA-A*11:01, HLA-A*24:02, and HLA-B*35:01, were searched by combining the results generated by these prediction tools (Figure 1B). To narrow down the list of potential epitopes, only those with the prediction score >21 in SYFPEITHI, <1 in IEDB and netCTLpan were selected. Both predicted (Figure 1B) and already identified (Figure 1C) epitopes were mapped to the reference sequences of PSA and PAP.

To further increase the potential immunogenicity of the polyantigen in the global human population, IEDB prediction was extended to 21 additional HLA-I alleles (Table S1). The total of 27 HLA-I alleles considered are shared by 97% of the human population. The vast majority of the resulted predicted epitopes was mapped to the same immunogenic regions identified previously, reinforcing the choice of potentially immunogenic regions. The selected immunogenic regions, which were prolonged with 3 aa residues on either side of each region, were then joined together. This resulted in 6 aa-long linkers. The resulting PAP-PSA fusion sequence was called "PROST-02". Except for PSA and PAP, no significant homology was found for the PROST-02 clustered antigenic sequences versus complete human protein database (https://www.uniprot.org), as scanned by BLAST (https://blast.ncbi.nlm.nih.gov/Blast.cgi). This is reassuring regarding the risk of non-specific autoimmune responses in case of the use of these sequences in prospective immuno-oncotherapy. A small number of putative junctional epitopes have been generated, none of which showed particular homology with the human protein database.

A non-integrative lentiviral vector, named "Lenti-PROST-02", was designed to encode the PROST-02 fusion antigen, under the transcriptional control of human β2m promoter (Figures 1D and S2).[12] This promoter contains conserved *cis*-regulatory elements: (1) interferon (IFN)-stimulated response elements (ISREs) that are the binding site for the IFN regulatory factors, and (2) SXY modules which interact with a multiprotein complex. ISREs and SXY transactivate pro-inflammatory cytokines, which are upregulated in maturing DCs.[25,26]

T cell immunogenicity of Lenti-PROST-02

T cell immunogenicity of Lenti-PROST-02 was evaluated by immunizing male C57BL/6 (H-2^b) mice (n = 5/group) via i.m. route at day 0 with 1×10^9 transduction units (TU) of Lenti-PROST-02 or an empty vector (Ctrl Lenti). On day 14, splenocytes from individual mice were used in IFNγ ELISPOT after *in vitro* stimulation with

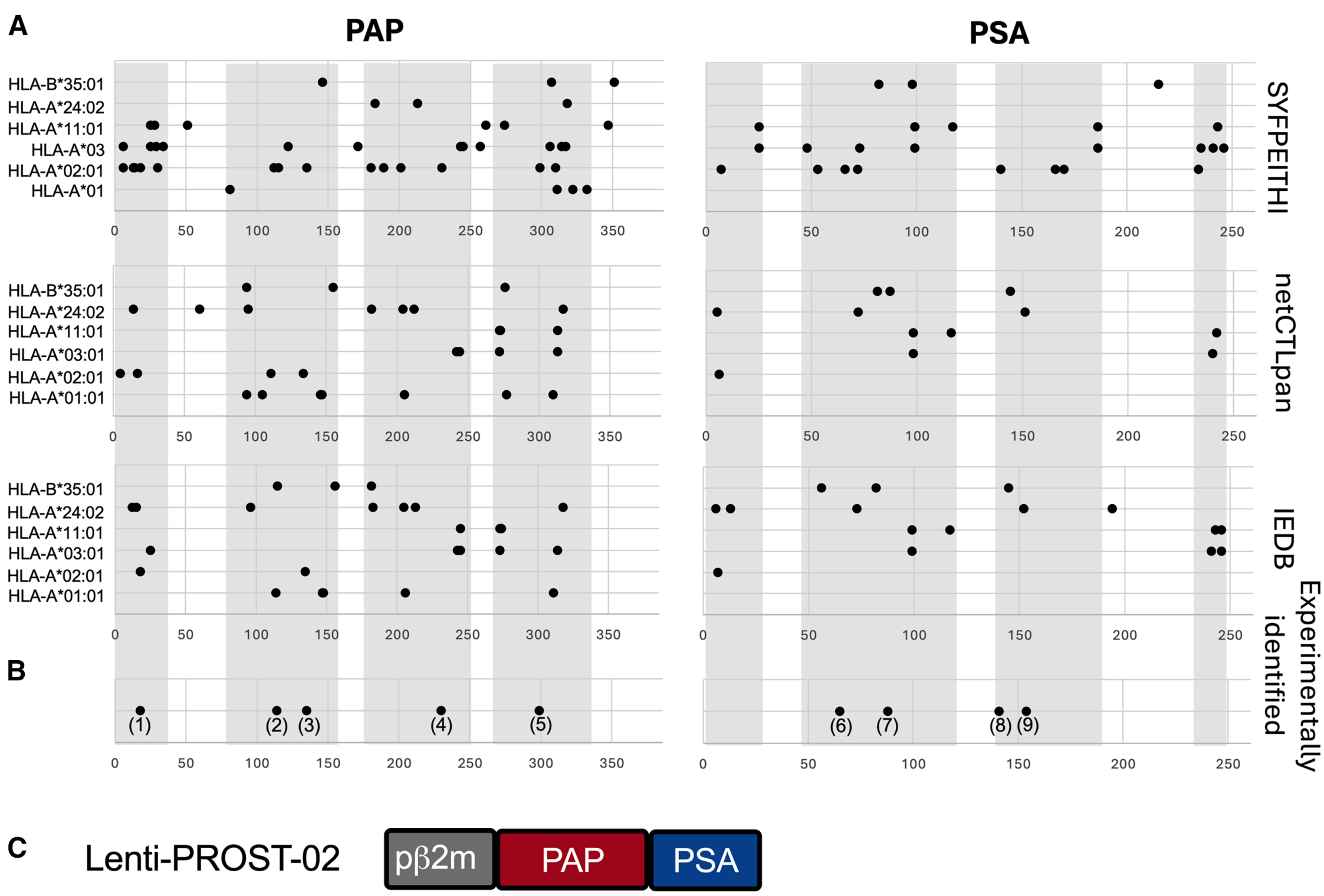

D **PAP**LPGPDD**PSA**

MRAAPLLLARAASLSLGFLFLLFFWLDRSVLAKELKFVTLVFRHGHYELGEYIRKRYRKFLNESYKHEQVYIRS
TDVDRTLMSAMTNLAALFPPEGVSIWNPILLWQPIPVHTVPLSEDQLLYLPFRNCPRFQELRLHPYKDFIATLG
KLSGLHGQDLFGIWSKVYDPLYCESVHNFTLPSWATEDTMTKLRELSELSLLSLYGIHKQKEKSHMKRATQIP
SYKKLIMYSAHDTTVSGLQMALDVYNGLLPPYASCHLTELYFEKGEYFVEMYYRNETQHEPYPLMLPGPDDW
VPVVFLTLSVTWIGAAPLILSRIVGGWECEKHSQRGRAVCGGVLVHPQWVLTAAHCIRNKSVILLGRHSLFHP
EDTGQVFQVSHSFPHPLYDMSLLKNRFLRPGDDSSHDLMLLRLGPDDMDLPTQEPALGTTCYASGWGSIEP
EEFLTPKKLQCVDLHVISNDVCAQVHPQKVTKFMLCAGRWTGGSEPCALPERPSLYTKVVHYRKWIKD

Figure 1. Selection of T cell epitope-containing regions of human PAP and PSA

(A) Distribution of predicted human MHC-I PAP- or PSA-specific epitopes by use of SYFPEITHI with a score >21, netCTLpan with a score <1, and IEDB with a score <1, applied to HLA-B*35:01, HLA-A*24:02, HLA-A*11:01, HLA-A*03, HLA-A*02:01, and HLA-A*01 alleles. (B) Experimentally identified human T cell epitopes as referenced in IEDB database for (1) HLA-A2, (2) HLA-DRB1*01:01, (3) HLA-A*02:01, (4) HLA-DR1, (5) HLA-A2, (6) HLA-DRB1*04:01, (7) H-2D^b, (8) HLA-A2, and (9) HLA-A2. Each dot corresponds to the first aa of an epitope and its position along the sequence of PAP or PSA (*x* axis). The regions highlighted in gray were selected to be included in the "PROST-02" antigen. (C) PROST-02 antigen composed of clusters of T cell epitopes derived from PAP (red) and PSA (blue), under the human β2m promoter, as encoded by Lenti-PROST-02 non-integrative vector. (D) Protein sequence of PROST-02 antigen containing 505 aa. The underlined gray characters indicate the linker between PAP and PSA segments.

pools of 15-mer peptides spanning the complete sequences of the PROST-02 antigen.

Splenocytes from the control mice did not respond to the peptide stimulation, while those from the Lenti-PROST-02-immunized mice were responsive to one PAP-derived and three PSA-derived peptide pools (Figure 2A). Subsequent incubation of splenocytes with each individual peptide of the positive pools allowed precise epitope mapping of the PROST-02 antigen and identification of at least 6 immunogenic regions in H-2^b mice (Figure 2B). Intracellular cytokine staining (ICS) of splenocytes from Lenti-PROST-02-immunized mice showed that the responding splenocytes were CD8$^+$ T cells, containing a majority of IFNγ$^+$ TNFα$^+$ cells (Figure 2C). By use of ICS, we identified the strongest individual peptides from each positive pool (Figure 2D) for further immunological studies. The weak PSA-derived epitopes (PSA #1 and #10) detected by ELISPOT were not detected by ICS, which is less sensitive (Figure 2D). The good immunogenicity of Lenti-PROST-02 in male mice against PAP #25 and PSA #21 peptides, which have strong homology between the murine and human sequences (Table S2; Figure S3), supports a vaccine-induced break in the expected natural tolerance against these "self" epitopes.

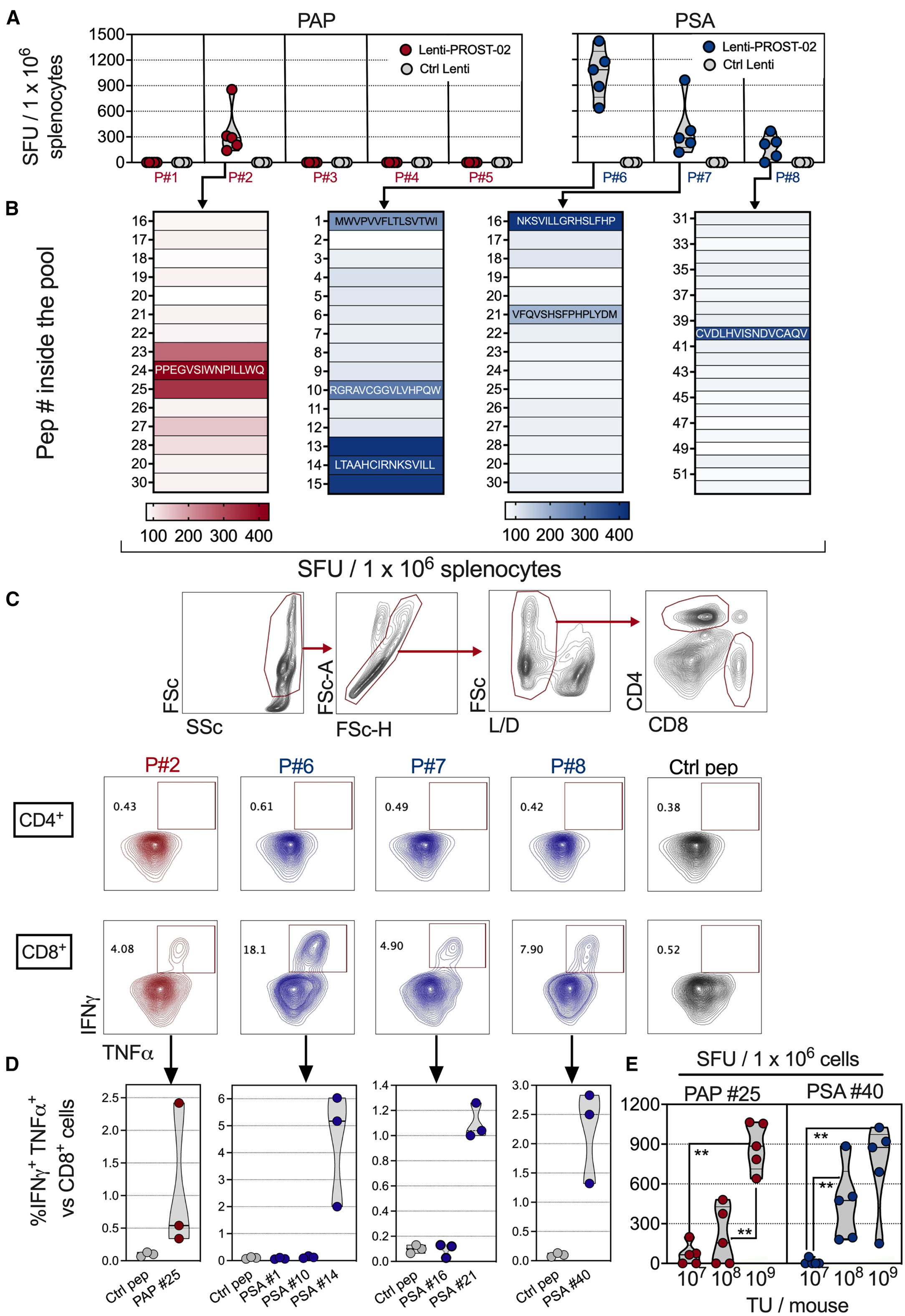

(legend on next page)

Immunization of C57BL/6 mice with escalating doses of Lenti-PROST-02 showed that the induced T cell immunity was dose dependent and that the dose of 1×10^9 TU generated the strongest responses (Figure 2E), in accordance with previous findings using other antigens.[17,27]

Lenti-PROST-02-based immunotherapy against a preclinical prostate cancer model

To evaluate the antitumor effect of Lenti-PROST-02, we started with the murine prostate tumor model TRAMP-C1,[28] an epithelial cell line isolated from the prostate of a transgenic mouse. However, these cells transduced to express human PSA or human PAP failed to develop tumors in male C57BL/6 recipients at any level of transgene expression, in contrast to the corresponding parental line. We also considered the RM-1 tumor model,[29] a prostate cell line derived from prostate cancer induced by Ras and Myc gene activation. However, RM-1 model presents a significant limitation as it is entirely MHC-I-deficient at the basal level, a phenotype far more extreme than that observed in human prostate tumors, which typically exhibit partial rather than complete MHC-I downregulation.[30,31] This led us to use the C57BL/6 syngeneic EL4 tumor line, which is commonly used in preclinical cancer models, even if it does not fully recapitulate all features of human prostate cancer. We transduced EL4 cells to stably produce the full-length human PSA. The "3E9" clone was selected for its highest capacity in PSA secretion and its MHC-I surface expression (Figures 3A and 3B). At least some sub-populations of neutrophils/granulocytes support tumor progression by favoring angiogenesis, stimulating tumor cell migration and promoting an immunosuppressive tumor microenvironment.[32] In this respect, subcutaneous (s.c.) 3E9 tumors, compared to the standard and well characterized model of HPV-induced TC-1 tumors, were particularly rich in $CD11b^+$ $Ly6C^{int}$ $Ly6G^+$ neutrophils/granulocytes (Figure 3C). In addition, 3E9 tumors had higher contents of IL10 (but not TGFβ) mRNA compared to the TC-1 tumors (Figure 3D).

Therefore, 3E9 model can, at minimum, recapitulate certain features of the cold and immunosuppressive tumor microenvironment observed in human prostate cancers.

The antitumor efficacy of Lenti-PROST-02 was assessed in male mice engrafted (s.c.) with 3E9 tumor cells (n = 7–8/group). Six days after tumor inoculation, when the tumors were well established, mice were treated by a single i.m. injection of 1×10^8 or 1×10^9 TU of Lenti-PROST-02 (Figure 3E). Tumor-bearing control mice received 1×10^9 TU of Ctrl Lenti. In all control mice, tumor volume reached 1,500 mm^3, defined as humane endpoint, before day 30. In opposite, an antitumor effect was detected in Lenti-PROST-02-treated mice in a dose-dependent manner (Figure 3F). The high dose of 1×10^9 TU led to a complete tumor eradication in 7/8 mice. Full tumor eradication was also observed in 3/8 mice with the suboptimal 1×10^8 TU dose (Figure 3F). The survival of the mice immunized with 1×10^9 TU of Lenti-PROST-02 was significantly prolonged (Figure 3G). The active secretion of human PSA by 3E9 tumors resulted *in vivo* in the presence of this antigen in the bloodstream, as measured on day 21 post-tumor transplantation (Figure 3H). Human PSA was not measurable in the blood of Lenti-PROST-02-treated mice, except for one mouse in which the tumor had not been eradicated. Therefore, as with physiological prostate cancers, in this preclinical model, PSA blood levels are an appropriate biomarker, reflecting the prostate tumor status.

The critical role of $CD8^+$ T cells in Lenti-PROST-02-induced tumor clearance was established using antibody depletion experiments. In 3E9 tumor-bearing mice, efficient depletion of $CD8^+$ T cells, but not $CD4^+$ T cells or NK cells, abolished vaccine-mediated tumor eradication (Figures 3J, 3K, and S4).

Among our numerous attempts in several syngeneic tumor lines to generate cell lines expressing human PAP and capable of forming solid tumors, only a clone of the MC38 line (colorectal tumor model)[33] transduced to express this protein ("MC38-PAP") could be selected. The anti-tumor efficacy of the lentiviral vector-based vaccine against MC38-PAP was demonstrated, especially when this treatment was combined with an anti-PD1 antagonist mAb (Figure S5). This does not necessarily mean that this therapy should be combined with an anti-PD1 mAb, but strengthens the preclinical proof-of-concept that the anti-huPAP T cells induced by the vaccine have an anti-tumor effector potential, even if the designed PAP segment only has one immunogenic region for C57BL/6 (H-2^b) mice.

These results demonstrated the antitumor potential of Lenti-PROST-02 immunotherapy in virulent prostate tumor models.

Impact of Lenti-PROST-02-treatment on intra-tumoral immune cells

To get more insights on the Lenti-PROST-02 mode of action, we studied the tumor cell microenvironment after the treatment. As

Figure 2. T cell immunogenicity of Lenti-PROST-02

(A) T cell responses of C57BL/6 mice, injected i.m. with 1×10^9 TU of Lenti-PROST-02 or Ctrl Lenti (n = 5/group). Frequencies of specific T splenocytes, determined by IFNγ ELISPOT at day 14 post-injection, in individual mice after *in vitro* stimulation with 5 or 3 distinct pools of 15-mer peptides, spanning respectively the PAP or PSA PROST-02 segments, and overlapping each other by 10 aa. SFU, spot-forming units. (B) Incubation of splenocytes from Lenti-PROST-02-immunized mice with each individual peptide of the positive peptide pools and studied by IFNγ ELISPOT. Heatmaps show SFU/1×10^6 splenocytes. The sequences of the immunogenic 15-mers are indicated inside the corresponding heatmap cells. (C) Cytometric gating strategy (top) and $CD4^+$ or $CD8^+$ T-splenocyte cytokine responses in immunized mice, as studied by ICS, after stimulation with derived from PAP or PSA (bottom). One representative mouse of 6/group is showed for each peptide. "L/D" = Live-Dead. (D) The same gating strategy as in (C) was applied. ICS-based study of $IFN\gamma^+$ $TNF\alpha^+$ $CD8^+$ T cell response of 3 individual Lenti-PROST-02-immunized mice to individual positive peptides from positive pools. (E) Dose-response of mice (n = 5/group), immunized i.m. with Lenti-PROST-02 escalating doses. T-splenocyte responses were studied by IFNγ ELISPOT at day 11 post-immunization. The results are representative of two independent experiments.

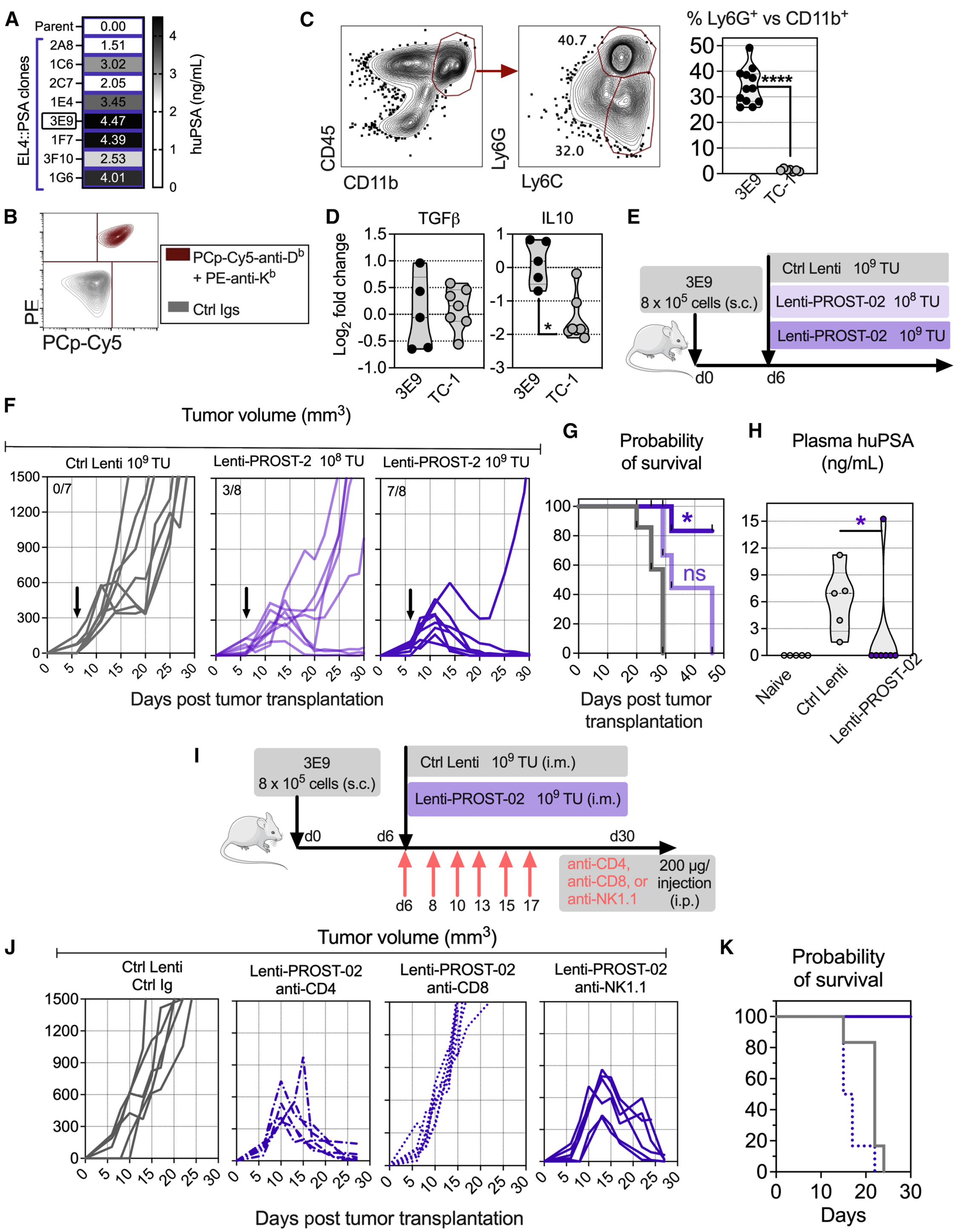

Figure 3. Lenti-PROST-02-based immunotherapy of tumors expressing human PSA

(A) Generation of a preclinical prostate tumor model. EL4 cells were transduced by an integrative lentiviral vector encoding the full-length human PSA, cloned and screened for the secretion of PSA. The clone "3E9" was selected for further experiments. (B) Cytometric profile of surface H-2D^b and H-2K^b expression of 3E9. (C) Cytometric gating strategy and proportions of intratumor Ly6G$^+$ Ly6C$^-$ cells within the CD11b$^+$ subset, determined on day 13 after 3E9 or TC-1 transplantation (s.c.) in C57BL/6 mice. (D) Log$_2$ fold change in TGFβ and IL10 mRNA expression in the 3E9 or TC-1 tumors. (E) Timeline of tumor engraftment and immunotherapy. C57BL/6 male mice (n = 7/group) were engrafted s.c. on the right flank with 8×10^5 3E9 cells. At day 6 post-tumor engraftment, mice were randomized for their tumor size and immunized i.m. with 1×10^8 or

(legend continued on next page)

early as day 9 after a single i.m. administration of 1×10^9 TU of Lenti-PROST-02, when the tumor shrinkage began (Figure 4A), the absolute numbers of $CD4^+$ or $CD8^+$ TILs normalized to tumor volume were significantly higher or showed an increasing trend respectively, compared to those in the growing tumors of control mice (Figures 4B and 4C). In the shrinking tumors of Lenti-PROST-02-treated mice, the $CD8^+$ T subset contained significantly higher proportions of $CD44^+$ KLRG1 (killer-cell lectin like receptor)$^+$ cells and $PD1^+$ TIM3 (T cell immunoglobulin domain and mucin domain)$^+$ cells, compared to the $CD8^+$ T subset in the control mice (Figure 4D). This predominance of activated/terminally differentiated $CD44^+$ $KLRG1^+$ and $PD1^+$ $TIM3^+$ $CD8^+$ T cells in the shrinking tumors likely resulted from their continuous antitumor activity.

In day 9 after Lenti-PROST-02 treatment, vaccine-induced PAP- or vaccine/tumor-induced PSA-specific $CD8^+$ TILs were detected after a 6 hour-coculture of TILs with syngeneic DCs pulsed with relevant PAP- (Figure 4E, top) or PSA- (Figure 4E, middle) derived peptides. Low levels of PSA-specific $CD8^+$ tumor-borne TILs were also detectable in control mice (Figure 4F). Lenti-PROST-02-treated mice had significantly higher levels of PSA-specific $CD8^+$ TILs, which were thus both tumor-borne and vaccine-induced (Figure 4F).

Among antigen-specific functional $CD8^+$ TIL subsets in Lenti-PROST-02-treated mice $IFN\gamma^+$ single-positive (SP) cells were largely predominant in both PAP- or PSA-specific $CD8^+$ TILs (Figure 4E). They displayed mainly a $PD1^+$ $TIM3^+$ activated/differentiated/exhausted phenotype (Figure 4G, top). Notably, the proportions of $PD1^+$ $TIM3^+$ cells inside $IFN\gamma^+$ SP subset were significantly higher in the PSA-specific T cells than in PAP-specific T cells (Figure 4H, left). This trait may result from a continuous triggering of PSA-specific $CD8^+$ TILs and sustained exercise of their effector functions against the PSA- (but not PAP-) expressing 3E9 tumors.

Antigen-specific $IFN\gamma^+$ SP population with $PD1^{dim}$ $TIM3^-$ phenotype contained a $CXCR3^+$ $CCR6^+$ subset (Figure 4G, bottom left), with presumable central/effector memory features. In parallel, antigen-specific $IFN\gamma^+$ SP subset with $PD1^+$ $TIM3^+$ phenotype contained a $CCR6^+$ subset (Figure 4G, bottom right), with possible memory properties. These two $PD1^{dim}$ $TIM3^-$ $CXCR3^+$ $CCR6^+$ (Figure 4H, middle) and $PD1^+$ $TIM3^+$ $CCR6^+$ (Figure 4H, right) subsets were less represented in the PSA-specific subset than in the PAP-specific subset, probably because PSA-specific T cells had more effector than memory features.

On day 9 after Lenti-PROST-02 treatment, $IFN\gamma^+$ $TNF\alpha^+$ $CD8^+$ T splenocytes specific for immunogenic regions of the PROST-02 fusion antigen were readily detectable (Figure 4I). These splenocyte responses were vaccine- but not tumor induced, as tumor-bearing control mice did not show such responses in their spleen (Figure 4I). Of note, despite the capacity of 3E9 tumor cells to secrete PSA *in vitro* (Figure 3A) and *in vivo* (Figure 3H), engraftment of these tumors per se did not induce PSA-specific $CD4^+$ T splenocyte responses either (Figure S6A). Principal component analysis (PCA) applied to the T cell responses to Lenti-PROST-02, the tumor size and the proportions of activated TILs showed that the group of mice with shrinking tumors clustered away from that of mice bearing growing tumors (Figure 4J).

The frequencies of granulocytes/neutrophils, which were widely represented in the 3E9 tumors (Figure 3C), and those of NK ($CD11b^{int}$ $NKP46^+$) cells, were statistically similar in tumors from both experimental groups (Figures S6B and S6C). The intratumor $CD11b^+$ F4/80^{hi} $CD11c^+$ MHC-II^+ population containing a $CD24^+$ cell subtype, which may correspond to a previously described monocyte-derived macrophage/cDC2 subset,[34] was well detected in the 3E9 tumors. Their frequencies were not statistically distinct in the two groups (Figure S6D).

In sum, a single Lenti-PROST-02 i.m. administration induced antigen-specific $CD8^+$ TILs with strong effector/differentiated phenotypes in 3E9 tumor-bearing mice.

Inflammatory status of tumors in Lenti-PROST-02-treated mice

The inflammatory status of the 3E9 tumors in the Ctrl Lenti- or Lenti-PROST-02-treated mice was analyzed by quantitative reverse-transcription PCR (RT-qPCR) on total tumor RNA. On day 7, no significant difference in the expression levels of inflammation-related analytes was observed between the two groups, with the exception for CXCL9, which exhibited elevated levels in the Lenti-PROST-02 group (Figure 5A). On day 9, in Lenti-PROST-02-treated mice, the tumors displayed globally a more inflammatory profile than in Ctrl Lenti-treated mice (Figure 5B). The expression of IL2 and IL12p40 was higher in the tumors of Lenti-PROST-02-treated mice (Figure 5B), and in a correlative manner (Figure 5C). In these mice, the higher intratumor expression of CCL2 and CCL5 (Figure 5B), in a correlative manner (Figure 5C), can contribute to tumor immune infiltration.[35] The increased expression of CCL19 and CCL21 can promote $IFN\gamma$-dependent antitumor immunity by enhancing the homing of $CCR7^+$ DCs and T cells.[36] The increased

1×10^9 TU of Lenti-PROST-02. Control mice received 1×10^9 TU of Ctrl Lenti. (F) Tumor size and (G) survival over the time. Concentration of hPSA in the plasma of naive controls or mice described in (E), determined on day 21. Note that 2 of 7 Ctrl Lenti-treated mice were already sacrificed at the time of blood sampling for this analysis due to tumor size exceeding 1,500 mm^3. Statistical significance for (C, D, and H) was determined by two-tailed Mann-Whitney test (*$p < 0.05$, ****$p < 0.0001$). The results shown in (C and D) are representative of two independent experiments. Those shown in (F and G) are representative of at least ten experiments. (I) Experiment timeline for *in vivo* phenotyping of anti-tumor cell effectors. C57BL/6 male mice ($n = 6$/group) were engrafted s.c. on the flank with 8×10^5 3E9 tumor cells. On day 6 post-tumor engraftment, mice were injected i.m. with Lenti-PROST-02 or Ctrl Lenti. Starting on day 6, mice received intraperitoneal injections of 200 μg/mouse/injection of anti-CD4 (clone GK1.5), anti-CD8 (clone H35.17.2), anti-NK.1 (PK136) mAb or Ctrl Ig every 2 or 3 days. (J) Tumor volume versus time. (K) Survival over the time. Statistical significance for (G and K) was determined by Log rank Mantel-Cox test (*$p < 0.05$). Mice were sacrificed when tumors reached 1,500 mm^3.

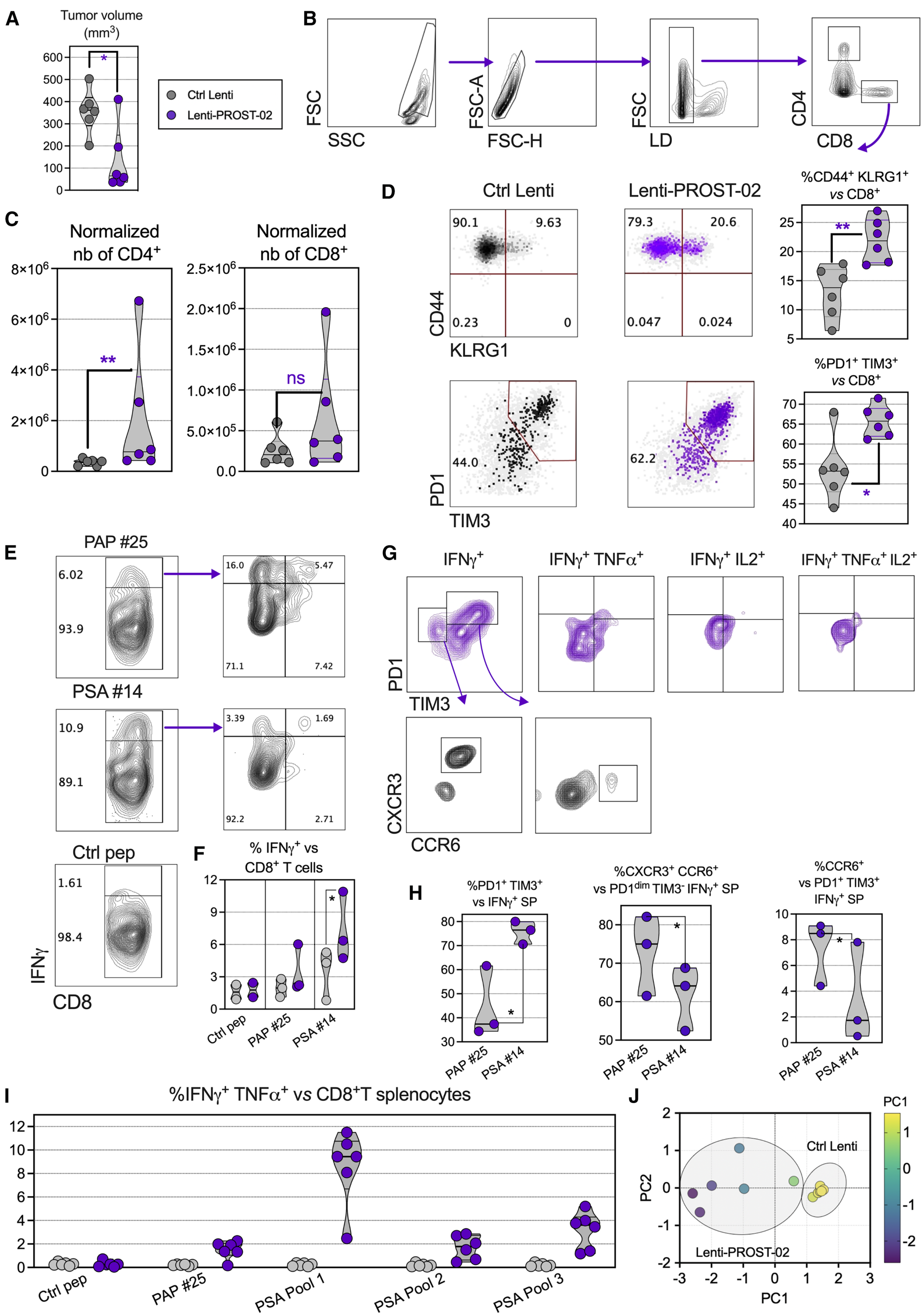

(legend on next page)

expression of CXCL5 may chemo-attract T cells and neutrophils and contribute to angiogenesis.[37] The upregulation of IFNγ-induced CXCL9 and CXCL10 is well known to cause recruitment of activated $CD8^+$ T cells.[38] The PCA projection of all the analytes studied on day 9 post-treatment divided the control and Lenti-PROST-02 individuals into two distinct groups (Figure 5D).

Therefore, a single i.m. Lenti-PROST-02 injection triggered a "cold-to-hot" tumor microenvironment shift by day 9, aligning with the kinetics of lentiviral vector-induced $CD8^+$ T cells, detectable ∼8–9 days post-vaccination.[12] This switch correlated with expanded activated/differentiated $CD8^+$ TILs and tumor regression, likely driven by vaccine-elicited $CD8^+$ T cell activity. This state is widely considered to be indicative of a favorable prognosis regarding to immune control of tumor growth.[39]

Long-term protective immune memory induced by Lenti-PROST-02 therapy

We determined whether the tumor-cured mice developed a protective immune memory. After an initial 3E9 tumor engraftment (s.c.) (n = 13), mice received a single i.m. injection of Lenti-PROST-02 which led to full tumor eradication (Figures 6A and 6B). To mimic tumor relapse, cured mice were re-challenged s.c. with 3E9 cells in the opposite flank on day 68 after the first tumor engraftment, and maintained without any additional treatment (Figures 6A and 6B). A group of naive mice received the same 3E9 tumor cell suspension, as a positive control of tumor growth (n = 7). All naive control mice developed tumors, while all initially cured mice were protected from the tumor re-challenge and were still alive 200 days after the initial tumor engraftment (Figure 6B).

To awaken antigen-specific memory T cells without triggering primary T cells,[17,40,41] on day 200 after the initial tumor transplantation, Lenti-PROST-02-treated cured mice (n = 6) or naive mice (n = 2) received an i.v. instillation of an adjuvant-free mixture of the immunogenic PSA #14 and PSA #40 peptides (Figure 6A). Two days later, splenocytes were stimulated *in vitro* with individual homologous peptides or an irrelevant peptide and studied by IFNγ ELISPOT (Figure 6C) and ICS and surface marker labeling (Figures 6D and 6E). PSA#14- or PSA#40-specific IFNγ-producing T splenocytes were detected in the cured mice after stimulation with homologous—but not an irrelevant control—peptides (Figures 6D–6F). The main subset of the responding $CD8^+$ T cells in the cured mice had a $CD44^+$ $CD62L^-$ $CD127^+$ $KLRG1^-$ phenotype (Figures 6E–6G), a hallmark of memory precursor T cells, detectable at this advanced time point of 200 days after the beginning of the experiment.[12]

To assess whether T cells distinct from vaccine-elicited PSA-specific T cells contribute to the protective immunity observed upon tumor rechallenge, we subjected Lenti-PROST-02-treated mice, previously cured of 3E9 tumors, to a rechallenge with the parental EL4 cell line, which lacks PSA expression (Figure 6H). Unlike naive controls, which exhibited robust tumor outgrowth, cured mice resisted EL4 rechallenge (Figure 6I). This observation indicates that the initial PSA-targeted T cell response induced by Lenti-PROST-02 also favors triggering of a durable, antigen-diversified memory response, likely via epitope spreading.[42] This process, wherein the initial immune response against a defined antigen (e.g., PSA) facilitates the priming of T cells against additional tumor-associated antigens, possibly released during therapy-induced tumor cell death, represents a critical hallmark of effective anti-tumor immunity and is strongly correlated with improved therapeutic outcomes.

In summary, a single i.m. injection of Lenti-PROST-02 elicits potent antitumor immunity, driven by prostate-specific antigen-targeted T cell responses. This intervention not only achieves complete tumor eradication but also fosters durable and broad immunological memory, which indicates its promise as a sustained and targeted immunotherapeutic strategy.

DISCUSSION

Lentiviral vectors efficiently transduce immature and non-dividing DCs.[13,14,43] The genetic message delivered by non-integrative lentiviral vectors remains in the nuclei of the transduced DCs, without interaction with the host-cell chromosome. These vectors are scarcely inflammatory.[43,44] Their transgene expression driven by β2m promoter enhances antigen presentation upon DC maturation and mobilization,[13,14] making them ideal for immuno-oncotherapy. Here, Lenti-PROST-02 elicited high-quality, prostate-specific T cell responses, overcoming immunosuppressive tumor microenvironment in a virulent murine tumor model. Treatment induced polyfunctional $CD8^+$ T cells targeting PAP/PSA epitopes and expanded

Figure 4. Status of TILs and T splenocyte responses induced by Lenti-PROST-02

C57BL/6 mice were engrafted s.c. with 3E9 cells on day 0 and treated with Lenti Ctrl or Lenti-PROST-02 on day 6 (n = 6/group). TILs were studied by cytometry on day 15, i.e., 9 days post-vaccination. (A) Tumor volume on day 9 post-vaccination. (B) The cytometric gating strategy. Note that 3E9 tumor cells express CD45 themselves and thus it was not possible to draw a gate on the $CD45^+$ hematopoietic cells and calculate the % of $CD4^+$ or $CD8^+$ TILs versus the $CD45^+$ population. (C) Absolute numbers of $CD4^+$ or $CD8^+$ TILs normalized to tumor volume. (D) Representative dot plots of $CD8^+$ TILs and their differentiation status in both groups and recapitulative percentages of various TIL subsets studied comparatively in both groups. (E) TILs were co-cultured with syngeneic bone-marrow-derived DC loaded with 10 μg of PAP #25 (top), PSA #14 (middle), or an irrelevant (bottom) peptide. Cells were stained at the surface and intracellular effector cytokines. To reach sufficient number of TILs per analysis, samples were pooled 2 by 2. Representative dot plots to detect various cytokine-expressing $CD8^+$ in Lenti-PROST-02 mice after *in vitro* restimulation with PAP#25, PSA#14, or an irrelevant peptide. (F) Percentages of total antigen-specific $IFN\gamma^+$ cells versus $CD8^+$ TILs. (G) PD1 versus TIM3 expression in each functional subsets (top) and CXCR3 versus CCR6 expression in various subsets of $IFN\gamma^+$ SP $CD8^+$ TILs after *in vitro* stimulation. (H) Recapitulative $\%PD1^+$ $TIM3^+$ versus $IFN\gamma^+$ SP, $\%CXCR3^+$ $CCR6^+$ versus $PD1^{dim}$ $TIM3^-$ $IFN\gamma^+$ SP, and $\%CCR6^+$ versus $PD1^+$ $TIM3^+$ $IFN\gamma^+$ SP $CD8^+$ TILs subset in PAP- or PSA-specific $CD8^+$ cells. (I) PAP- and PSA-specific $IFN\gamma^+$ $TNF\alpha^+$ T splenocyte responses. (J) PCA projection of all parameters studied in (A–I). Proportion of variance for PC1 = 87.62%. Results are representative of two independent experiments. Statistical significance was evaluated using Mann-Whitney tests (*p < 0.05).

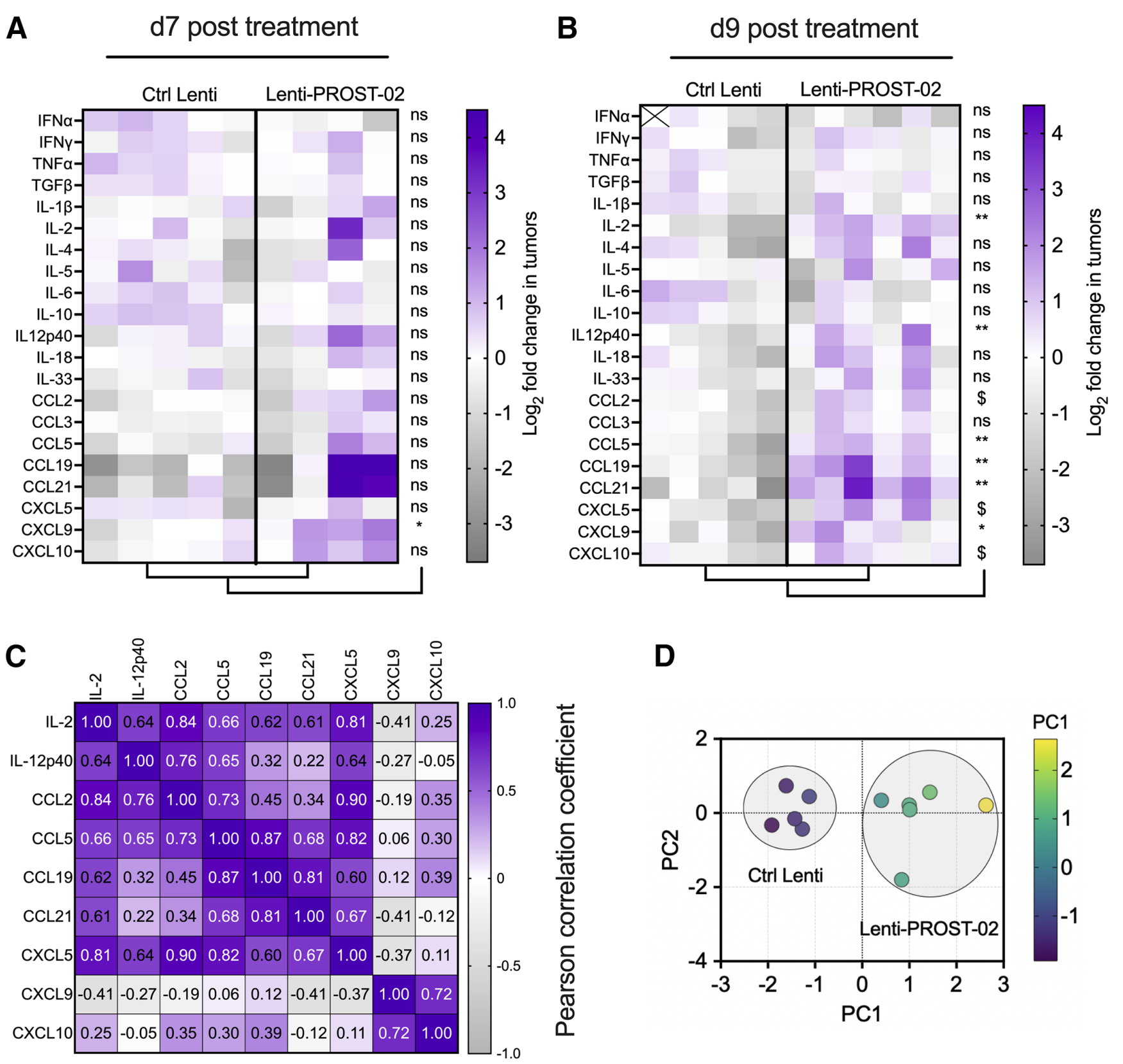

Figure 5. Inflammatory status of the 3E9 tumors in control or Lenti-PROST-02-treated mice

(A and B) Log_2 fold change in mRNA expression in the tumors of mice (n = 5 or 4–6/group), treated with Ctrl Lenti or Lenti-PROST-02 on day 6, and analyzed by RT-PqCR on day 7 (A) or 9 (B) post-vaccination. For the control and Lenti-PROST-02 groups, Mean ± SD of tumor volumes were respectively 218 ± 145 and 244 ± 258 (statistically not different) at day 7 and 357 ± 98 and 135 ± 148 (statistically different, $^*p < 0.05$) at day 9 post-vaccination. For each sample, the mRNA abundance (C_T value) of the target genes was normalized to that of the succinate dehydrogenase complex, subunit A (SDHA) gene and compared to a calibrator value corresponding to the mean of the C_T values determined in the tumors from the Ctrl Lenti mice. The fold change in gene expression was then calculated using $2^{-\Delta\Delta C_T}$. Statistical significance was evaluated using Mann-Whitney tests (ns: not significant, $^{\$}p < 0.1$, $^*p < 0.05$, $^{**}p < 0.01$). One outlier sample was discarded from the analyses in the group of Ctrl Lenti for IFNα analyte. (C) Pearson correlation coefficient of differentially expressed analytes in the tumors of Lenti-PROST-02-treated mice on day 9 post-vaccination. (D) PCA projection of all analytes studied in (B). Proportion of variance for PC1 = 62.01%. Results shown are representative of two independent experiments.

$CD44^+$ $PD1^+$ $TIM3^+$ $KLRG1^+$ effector $CD8^+$ TILs, reflecting sustained antitumor activity. In Lenti-PROST-02-treated mice, shrinking tumors exhibited a “cold-to-hot” inflammatory shift, indicating immune microenvironment remodeling. The primary antitumor mechanism involves vaccine-induced $CD8^+$ T cells, while the immunostimulatory microenvironment transition, known to enhance immunotherapy efficacy,[39] likely arises secondarily from effector T cell activity. We have also observed a “cold-to-hot” inflammatory switch in the TC1 model, which is the standard preclinical model of HPV-induced cancers.[17,18] A single i.m. injection of the «Lenti-HPV-07» vector, which encodes detoxified HPV16/18 E6/E7 oncoproteins, triggers even more intense tumor microenvironment remodeling than the Lenti-PROST-02 vector does in 3E9 tumors. Similarly, the “Lenti-KRAS” vector, encoding the mutated $KRAS^{G12D}$ antigen, induces significant intratumoral $CD8^+$ T cell recruitment and an inflammatory shift in the MC38-$KRAS^{G12D}$ model,[45] though it is less pronounced than in the TC1 and 3E9 models. The intensity and breath of this “cold-to-hot” inflammatory shift appear to depend to the T cell immunogenicity of the antigen encoded by the lentiviral vector in the recipients. This includes the MHC alleles they harbor, as well as the tumor’s intrinsic immunological and microenvironmental properties.

We also observed that the initial, well-directed Lenti-PROST-02-induced T cell response lays the groundwork for a sustained, broad-spectrum memory immune response. This sets the stage for the subsequent recognition and response to a wider array of tumor-associated antigens, which are seemingly released following the efficient lysis of tumor cells. This reduces the likelihood of tumor escape and relapse. The establishment of this adaptive, antigen-diversified T cell immunity is a critical advancement in our understanding of how targeted immunotherapies based on robust immunization platforms can achieve long-lasting anti-tumor outcomes. The broadened immune response and secondary tumor antigens contributing to epitope spreading will be identified in future studies via combined transcriptomic/proteomic/MHC-I immunopeptidomic profiling of EL4 and 3E9 tumors, alongside TCR sequencing of $CD8^+$ memory T cells. This will allow tracking of expanded T cell clones and defining the molecular mechanisms of epitope spreading in memory responses.

Our previous experimental data and comparative literature analyses consistently demonstrate the superior antitumor efficacy of lentiviral vectors over alternative vaccine platforms. When targeting the same antigen in identical preclinical tumor models, lentiviral vectors outperformed adjuvanted proteins, adenoviral, arenaviral, and poxviral vectors, as well as DNA- or LNP-mRNA-based vaccines.[12,13,19,46] Additionally, lentiviral vectors offer distinct safety advantages due to their low inflammatory profile.[43,44] They also achieve maximal effectiveness at relatively low doses (10^8–10^9 TU), whereas

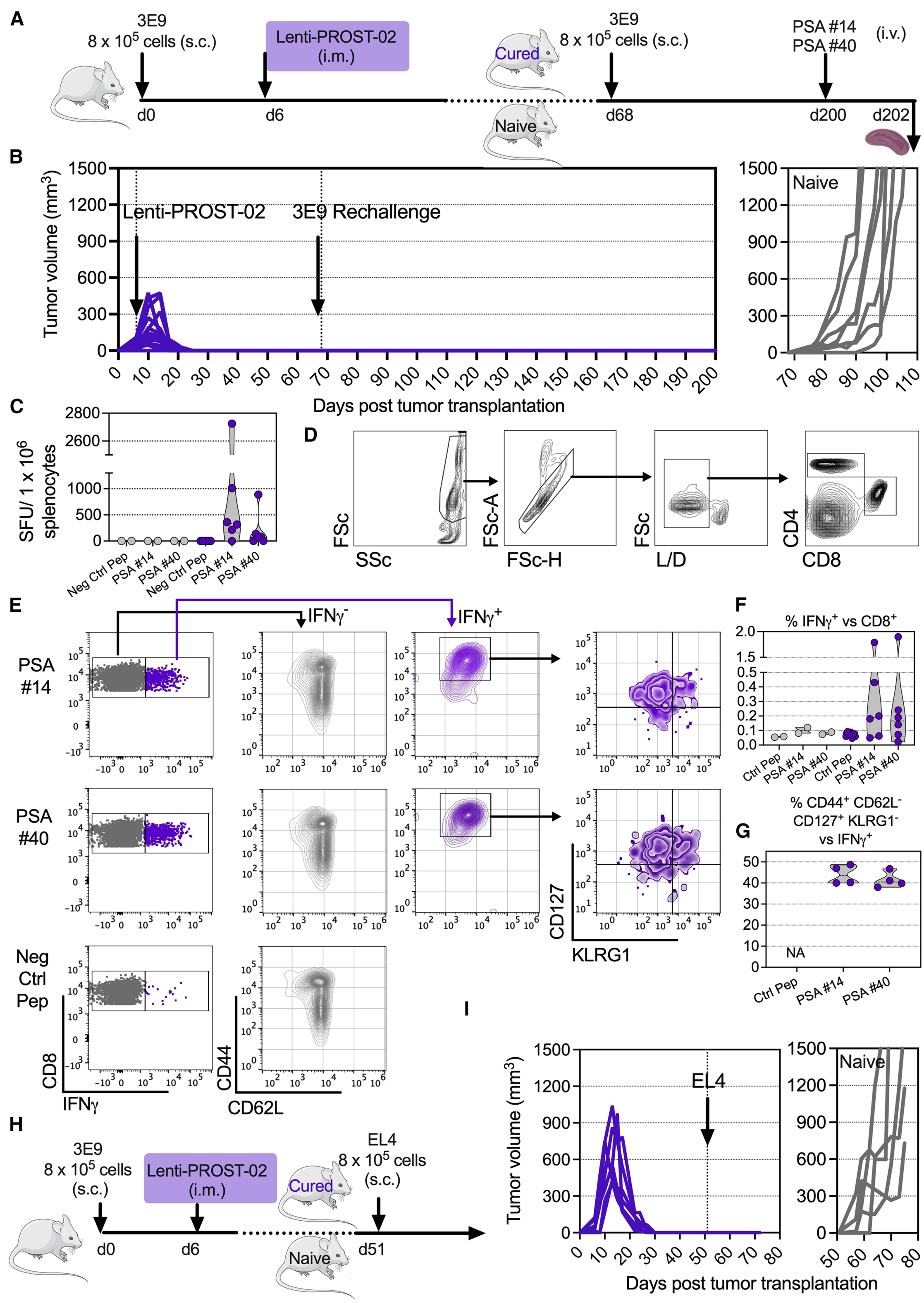

(legend on next page)

adenoviral vectors require much higher particle numbers (10^{11}1012 infectious genome units; IGU) to reach comparable efficacy.[46] The need for higher doses not only significantly increases the risk of adverse effects but also results in high costs and limitations in the scalability of GMP production.

Lenti-PROST-02 was engineered using HLA-I-restricted epitopes derived from PSA and PAP for human immunotherapy. Despite preclinical validation conducted exclusively in mice, the vector was designed to bypass the need for reformulation, enabling direct clinical translation. This was achieved by leveraging the cross-species immunogenicity of the PROST-02 polyantigen, which, though optimized for HLA-I, retained immunogenic regions functional in H-2^b mice (Table S2). Thus, the same construct validated preclinically remains clinically applicable without modification, streamlining development for human immuno-oncotherapy trials.

Human prostate tumors exhibit low immunogenicity and an immunosuppressive microenvironment,[7] lacking high mutational burden, microsatellite instability, or mismatch repair defects to generate targetable neoantigens.[47,48] Consequently, CTLA-4/PD-1/PD-L1 blockade shows limited efficacy in mCRPC.[11] Therefore, promoting immune responses against antigens, actively produced by prostate tumor, with an expression profile severely restricted to this organ, can be promising. Advanced immunotherapeutic targets in clinical trials include (1) PAP-targeted DNA vaccines + anti-PD1 mAbs, (2) viral vectors encoding PSA/carcinoembryonic antigen/mucin-1 ± co-stimulatory molecules, (3) adenoviral Ad5 vectors encoding PSA/mucin-1/Brachyury antigens, (4) recombinant New York esophageal squamous cell carcinoma-1 (NY-ESO-1) + CpG ODNs, and (5) telomerase-derived peptides + granulocyte-macrophage colony stimulating factor (GM-CSF).[11,49] PSMA-based immunotherapy carries autoimmune risk due to nervous system expression.

Sipuleucel-T, the only FDA-/EMA-approved antigen-based immunotherapy for asymptomatic or minimally symptomatic mCRPC, involves *ex vivo* incubation of autologous $CD54^+$ antigen-presenting cells with a PAP-GM-CSF fusion protein, followed by i.v. reinfusion.[9] GM-CSF directs uptake via DC receptors, and treatment requires three leukaphereses every two weeks. Clinically, it extended median survival by 4.1 months and reduced mortality risk by 22.5% versus placebo,[8,10] validating antigen-specific immunotherapy in mCRPC. However, recombinant protein loading of DCs typically favors MHC-II over MHC-I presentation, raising questions about PAP cross-presentation efficiency and $CD8^+$ T cell induction.[50] Nevertheless, the relative success of Sipuleucel-T promoted development of simpler, targeted immunotherapies, avoiding *ex vivo* manipulation, with lower cost and risk.[11,49] Lentiviral vectors demonstrate superior MHC-I antigen presentation and durable immunity compared to adjuvanted proteins, peptides, or alternative viral vectors, including adenoviral, arenaviral, poxviral, including modified vaccinia Ankara (MVA), and DNA- or mRNA-based platforms.[19] Preclinical comparisons in HPV-induced TC1 cancer model revealed that only lentiviral vectors achieved complete tumor eradication with prolonged immune memory, suggesting broad applicability in immuno-oncotherapy. Their natural DC tropism enables *in vivo* transduction, eliminating the need for *ex vivo* manipulation (e.g., leukapheresis) while promoting endogenous MHC-I-mediated antigen presentation, enhancing therapeutic potential.

Immunotherapeutic strategies targeting $CD8^+$ T cell responses in prostate cancer must address the critical challenge of HLA-I downregulation, that undermines T cell-mediated cytotoxicity. HLA-I downregulation occurs in $\approx 70\%$ of primary prostate tumors, with complete loss detected in $\approx 34\%$ of primary tumors and $\approx 80\%$ of metastatic lesions.[30,31] The loss of HLA-I is frequently linked to reversible epigenetic modifications, particularly DNA methylation and histone H3 tri-methylation, which reduce chromatin accessibility and transcriptional activity.[51] However, these alterations can be counteracted through epigenetic modulators including DNA methyltransferase inhibitors and histone deacetylase inhibitors which effectively restore HLA-I surface expression. Histone deacetylase inhibitors upregulate HLA-I in prostate tumors by increasing β2m expression and have been proposed to be advantageously combined to T cell-based immunotherapies of prostate cancers.[31] Most of the components of the antigen presentation machineries and surface HLA-I can also upregulate under the action of IFNγ.[52] Therefore, immunotherapeutic approaches that enhance IFNγ signaling, such as tumor-localized IL12 immunotherapy,[53] or IFNγ-inducing adjuvants like CpG oligodeoxynucleotides,[11] can reverse HLA-I downregulation and promote an immune-permissive tumor microenvironment. These strategies can be advantageously combined with

Figure 6. Lenti-PROST-02 therapy induces a long-term antitumor immune memory which prevents tumor relapse

(A) Timeline of the tumor engraftment and immunotherapy. C57BL/6 mice ($n = 13$) were engrafted s.c. on the right flank with 8×10^5 3E9 cells. At day 6 post-engraftment, mice were randomized and treated i.m. with 3×10^8 TU of Lenti-PROST-02. (B) Tumor growth. On day 68, the cured mice were rechallenged with 8×10^5 of 3E9 cells in the opposite flank and left untreated to mimic a prostate tumor relapse. Naive mice ($n = 7$), injected s.c. with the same 3E9 cell suspension showed the growth potential of the engrafted tumor cells used in the relapse experiment (B, right). The cured mice in which the tumor relapse was unable to occur received on day 200 an i.v. infusion of a mixture of 25 μg of PSA #14 and 25 μg of PSA #40 peptides. Two days later, splenocytes were recovered and stimulated *in vitro* with homologous peptides or an irrelevant peptide. (C) Frequencies of PSA#14- or PSA#40-specific T splenocytes, determined by IFNγ ELISPOT. (D and E) Cytometric gating to detect IFNγ-producing PSA-specific $CD8^+$ T cells. (F) Percentages of PSA-specific $IFN\gamma^+$ cells compared to total $CD8^+$ T splenocytes. (G) Percentages of $CD44^+$ $CD62L^-$ $CD127^+$ $KLRG1^-$ memory precursor cells versus $IFN\gamma^+$ $CD8^+$ T cells in the 4 mice with antigen-specific response detectable by ICS. NA = not applicable. Results shown in (B) are representative of three independent experiments. (H) Experiment timeline. Cured mice ($n = 7$) were rechallenged on day 51 with 8×10^5 of PSA-negative parental EL4 cells in the opposite flank and left untreated. Naive mice ($n = 6$), injected s.c. with the same EL4 cell suspension showed the growth capacity of the engrafted cells. (I) EL4 tumor growth in cured (left) or naive control (right) mice.

therapeutic vaccines, including Lenti-PROST-02. In summary, overcoming HLA-I downregulation is essential for the success of $CD8^+$ T cell immunotherapies in prostate cancer. Epigenetic modulation, IFNγ-inducing adjuvants, and IL12-based immunotherapy represent promising approaches to restore MHC-I presentation, thereby potentiating the efficacy of T-cell-targeted treatments and improving patient responses in an otherwise immunosuppressive tumor context. Unlike some human prostate tumors, the 3E9 tumor does not exhibit MHC-I downregulation *in vivo*. Consequently, we could not evaluate the effects of combining Lenti-PROST-02 with MHC-I restoration therapies in this model. Future studies will explore combining Lenti-PROST-02 with therapeutic strategies to counteract MHC-I downregulation when an adapted preclinical tumor model is available.

It is important to note that preclinical murine models underestimate the efficacy of lentiviral vectors in primates due to species-specific transduction differences.[13,54] This is supported by non-human primate studies and clinical trials,[55] (NCT06319963) where doses of 10^8–10^9 TU/individual, similar to murine doses, demonstrated strong immunogenicity. This dose parity reflects the weak transduction efficiency of lentiviral vectors in murine cells relative to primate cells. A prospective Lenti-PROST-02 clinical trial will incorporate a dose-escalation phase to rigorously evaluate the safety, immunogenicity, and efficacy across multiple dose levels in humans.

A phase 1/2a trial (NCT06319963) using a lentiviral vector encoding HPV16/18 oncoproteins was initiated for HPV-associated oropharyngeal or cervical cancer, supported by preclinical murine efficacy.[17,18] Prior clinical trials confirmed safety, tolerability, immunogenicity, and non-genotoxicity of lentiviral vectors (2011-006260-52).[15,16] With growing clinical adoption, this approach is poised for broader human immunotherapy applications. We are currently extending the lentiviral vector-based immunotherapy to non-viral cancers, starting with prostate cancer.

MATERIALS AND METHODS

Lenti-PROST-02

Codon-optimized sequence of the designed PROST-02 antigen was synthesized by Genecust (Boynes, France) and inserted into the pFlap lentiviral plasmid, between the BamHI and XhoI sites, located between the human β2-microglobulin promoter and the mutated *atg* starting codon of the woodchuck post-transcriptional regulatory element and production of non-integrative Lenti-PROST-02 vector was performed as described.[12,17] The integrase resulting from the packaging plasmid carries a missense D64V mutation in its catalytic triad which prevents the integration of viral DNA into the host chromosome.[13]

Mice and immuno-oncotherapy

Six- to 12-week-old male C57BL/6JRj mice (Janvier, Le Genest Saint Isle, France) were housed in ventilated cages under specific pathogen-free conditions at the Institut Pasteur animal facilities. All methods were carried out in accordance with ARRIVE regulations (https://arriveguidelines.org) and in accordance with the European and French guidelines (Directive 86/609/CEE and Decree 87–848 of 19 October 1987) after approval of the protocol by the Institut Pasteur Safety, Animal Care and Use Committee delivered by the local ethics committee (CETEA #DAP180049, CETEA #DAP190130 and Ministry of High Education and Research APAFIS#16381–2018080217194542 v1, APAFIS# 20981–20190606164112731.

The EL4 cells (ATCC, TIB-39) were transduced by an integrative lentiviral vector encoding the full-length hPSA under the ubiquitin promoter.[56,57] The transduced EL4 cells were cloned and screened for their potential to secrete hPSA, determined by hPSA DuoSet ELISA kit (Biotechne). The EL4::PSA “3E9” clone was selected for its capacity to secrete high amounts of hPSA. 3E9and EL4 cells were cultured in DMEM supplemented with 10% FBS, penicillin (100 U/mL), and streptomycin (100 μg/mL).

Mice were engrafted s.c. with 8×10^5 3E9 cells and were immunized i.m. with Lenti-PROST-02 in 50 μL. The tumors were measured 2 to 3 times a week using a digital caliper, in a blind manner. The tumor volume was calculated using the formula $V = L \times W^2/2$, where V is the volume, L the longest diameter, and W the shortest diameter. Mice were killed when tumors reached 1,500 mm^3. Depletion using specific antibodies was conducted as described previously.[17,58]

T cell assays

Fifteen-mer peptides spanning the PAP or PSA segments included in PROST-02 were synthetized by Genscript and were used at final concentration of 1 μg/mL for ELISPOT or 2–5 μg/mL for ICS. T cell assays were performed in RPMI1640 supplemented with 10% FBS, penicillin, streptomycin, 1×10^4 M non-essential aa, 1% HEPES, 1×10^3 M sodium pyruvate and 5×10^5 M of β-mercapto-ethanol. ELISPOT and ICS on splenocytes were performed as described.[43,58]

Cytometric analysis of tumor immune infiltrates

Tumors were digested by use of Collagenase and DNAseI and were homogenized in a GentleMacs as described.[58] Tumor cell suspensions were filtered through 70 μm-pore filters, washed in PBS and stained as follows.

To study T cell activation status, cells were stained with a mixture of (1) eF450-anti-CD4 (RM4-5), APC-anti-CD8 (53–6.7), FITC-anti-CD44 (IM7), and PerCP-eF710-anti-KLRG1 (2F1) or (2) PerCp-Cy5.5-anti-CD4 (RM4-5), BV711-anti-CD8 (53–6.7), FITC-anti-PD1 (J43), and PE-Cy7-anti-TIM3 (RMT3-23). To study MHC-I expression on tumors, cells were stained with PE-anti-H-$2K^b$ (REA1198) and PerCP-Cy5.5-anti-H-$2D^b$ (KH95). All mixtures contained FcγII/III receptor blocking mAb (2.4G2) and near IR Live/Dead.

Tumor homogenates were enriched in lymphocytes on Ficoll and co-cultured in 24-well plates with syngeneic bone-marrow-derived DC pulsed with 10 μg/mL of various peptides for 6 h in the presence of anti-CD28 and anti-CD49d mAbs. During the last 3 h of incubation

monensin and brefeldin A, each at 1 μg/mL, were added. Cells were then surface stained with a mixture of eF450-anti-CD4 (RM4-5), BV711-anti-CD8 (53–6.7), BV510-anti-PD1 (29F.1A12), PE-Dazzle-594-anti-KLRG1 (2F1), and PE-Cy7-anti-TIM3 (RMT3-23).

All mixtures contained FcγII/III receptor blocking mAb and near IR Live/Dead. Cells were permeabilized using the Cytofix/Cytoperm kit for ICS by use of APC-anti-IFNγ (XMG1.2), FITC-anti-TNFα (MP6-XT22), and PE-anti-IL2 (JES6-5H4) mAbs.

Cells were fixed with Cytofix before acquisition in an Attune NxT cytometer. The data were analyzed using FlowJo.

Cytometric analysis of antigen-specific TILs

To determine activation/differentiation status of antigen-specific TILs, tumor homogenates, prepared as mentioned previously, were enriched in lymphocytes on Ficoll gradient and were co-cultured in 24-well plates with syngeneic bone-marrow-derived DC pulsed with 10 μg/mL of PAP #25, PSA #14 or a negative irrelevant peptide for 6 h in the presence of anti-CD28 and anti-CD49d mAbs. During the last 3 h of incubation monensin and brefeldin A, each at 1 μg/mL, were added to block cytokine secretion. Cells were then washed and surface stained with a mixture of eF450-anti-CD4 (RM4-5), BV711-anti-CD8 (53–6.7), BV510-anti-PD1 (29F.1A12), PE-Dazzle-594-anti-KLRG1 (2F1/KLRG1), PE-Cy7-anti-TIM3 (RMT3-23), PerCP-Cy5.5-anti-CXCR3 (S18001A), and BV605-anti-CCR6 (29-2L17). All mixtures contained FcγII/III receptor blocking anti-CD16/CD32 and Near IR Live/Dead. Cells were then permeabilized using the Cytofix/Cytoperm kit for ICS as detailed previously.

Cytometric detection of memory CD8$^+$ T splenocytes

Splenocytes from cured mice were cultured for 6 h in the presence of 2 μg/mL of various peptides and anti-CD28 and anti-CD49d mAbs. Monensin and brefeldin A were added as detailed above. Cells were surface stained with eF450-anti-CD4 (RM4-5), BV711-anti-CD8 (53–6.7), FITC-anti-CD44 (IM7), AF700-anti-CD62L (MEL-14), PE-Cy5-anti-CD127 (A7R34) and PE-Dazzle-594-anti-KLRG1 (2F1), FcγII/III receptor blocking mAb, and near IR Live/Dead. Cells were then permeabilized for ICS with APC-anti-IFNγ mAb.

RT-qPCR

The tumor inflammatory mediators from treated mice were studied using the primer pairs listed in Table S3, and the comparative $\Delta\Delta C_T$ method, as published.[17,58]

Statistical analyses

Statistical analyses were performed using Prism GraphPad v.9.

DATA AND CODE AVAILABILITY

The published article includes all datasets generated and analyzed during this study. All plasmids and lentiviral vectors generated in this study will be available under an MTA for research use, given a pending patent directed to Lenti-PROST-02 vaccination vector. Further information and requests for resources and reagents should be directed to and will be fulfilled by the corresponding author (laleh.majlessi@pasteur.fr).

ACKNOWLEDGMENTS

This work was supported by Institut Pasteur and TheraVectys. The authors are grateful to Dr. Vincenzo Di Bartolo (Institut Pasteur) for providing the EL4 tumor cell line.

AUTHOR CONTRIBUTIONS

B.V., I.F., and S.C. performed most of the *in vivo* and *in vitro* experiments and formal analyses. F.M., A.N., and C.B. carried out the various stages of plasmid synthesis and lentiviral vector production and concentration. L.D. performed *in vivo* antitumor experiments and quantified human PSA in the circulation. P.A. and F.L.C. contributed to the antitumor *in vivo* experiments. F.N. initiated the tumor and immuno-oncotherapy model. K.N. contributed to the antigen design and epitope prediction. L.M. conceptualized the studies, supervised the project and wrote the manuscript, P.C. initiated and conceptualized the studies and funded the project.

DECLARATION OF INTERESTS

P.C. is the founder and CSO of TheraVectys. B.V., I.F., F.M., A.N., L.D., P.A., F.L.C., F.N., and K.N. are or have been employees of TheraVectys. L.M. had a consultancy activity for TheraVectys. B.V., I.F., F.M., L.D., P.A., F.L.C., L.M. and P.C. are inventors of a pending patent directed to the potential of Lenti-PROST-02 vaccination against prostate cancers.

SUPPLEMENTAL INFORMATION

Supplemental information can be found online at https://doi.org/10.1016/j.omton.2026.201299.

REFERENCES

1. Bray, F., Ferlay, J., Soerjomataram, I., Siegel, R.L., Torre, L.A., and Jemal, A. (2018). Global cancer statistics 2018: GLOBOCAN estimates of incidence and mortality worldwide for 36 cancers in 185 countries. CA Cancer J. Clin. *68*, 394–424. https://doi.org/10.3322/caac.21492.
2. Siegel, R.L., Miller, K.D., and Jemal, A. (2018). Cancer statistics, 2018. CA Cancer J. Clin. *68*, 7–30. https://doi.org/10.3322/caac.21442.
3. Mazzone, E., Preisser, F., Nazzani, S., Tian, Z., Bandini, M., Gandaglia, G., Fossati, N., Montorsi, F., Graefen, M., Shariat, S.F., et al. (2019). The Effect of Lymph Node Dissection in Metastatic Prostate Cancer Patients Treated with Radical Prostatectomy: A Contemporary Analysis of Survival and Early Postoperative Outcomes. Eur. Urol. Oncol. *2*, 541–548. https://doi.org/10.1016/j.euo.2018.10.010.
4. Crawford, E.D., Bryce, A.H., Hussain, M.H., Agarwal, N., Beltran, H., Cooperberg, M.R., Petrylak, D.P., Shore, N., Spratt, D.E., Tagawa, S.T., et al. (2024). Expert Perspectives on Controversies in Castration-Sensitive Prostate Cancer Management: Narrative Review and Report of the First US Prostate Cancer Conference Part 1. JU Open Plus *2*, e00029. https://doi.org/10.1097/ju9.0000000000000137.
5. Rebello, R.J., Oing, C., Knudsen, K.E., Loeb, S., Johnson, D.C., Reiter, R.E., Gillessen, S., Van der Kwast, T., and Bristow, R.G. (2021). Prostate cancer. Nat. Rev. Dis. Primers *7*, 9. https://doi.org/10.1038/s41572-020-00243-0.
6. von Amsberg, G., Alsdorf, W., Karagiannis, P., Coym, A., Kaune, M., Werner, S., Graefen, M., Bokemeyer, C., Merkens, L., and Dyshlovoy, S.A. (2022). Immunotherapy in Advanced Prostate Cancer-Light at the End of the Tunnel? Int. J. Mol. Sci. *23*, 2569. https://doi.org/10.3390/ijms23052569.
7. Shiao, S.L., Chu, G.C.Y., and Chung, L.W.K. (2016). Regulation of prostate cancer progression by the tumor microenvironment. Cancer Lett. *380*, 340–348. https://doi.org/10.1016/j.canlet.2015.12.022.
8. Anassi, E., and Ndefo, U.A. (2011). Sipuleucel-T (provenge) injection: the first immunotherapy agent (vaccine) for hormone-refractory prostate cancer. P T. *36*, 197–202.
9. Kantoff, P.W., Higano, C.S., Shore, N.D., Berger, E.R., Small, E.J., Penson, D.F., Redfern, C.H., Ferrari, A.C., Dreicer, R., Sims, R.B., et al. (2010). Sipuleucel-T immunotherapy for castration-resistant prostate cancer. N. Engl. J. Med. *363*, 411–422. https://doi.org/10.1056/NEJMoa1001294.
10. Thara, E., Dorff, T.B., Pinski, J.K., and Quinn, D.I. (2011). Vaccine therapy with sipuleucel-T (Provenge) for prostate cancer. Maturitas *69*, 296–303. https://doi.org/10.1016/j.maturitas.2011.04.012.

11. Sridaran, D., Bradshaw, E., DeSelm, C., Pachynski, R., Mahajan, K., and Mahajan, N.P. (2023). Prostate cancer immunotherapy: Improving clinical outcomes with a multi-pronged approach. Cell Rep. Med. *4*, 101199. https://doi.org/10.1016/j.xcrm.2023.101199.

12. Ku, M.W., Authié, P., Nevo, F., Souque, P., Bourgine, M., Romano, M., Charneau, P., and Majlessi, L. (2021). Lentiviral vector induces high-quality memory T cells via dendritic cells transduction. Commun. Biol. *4*, 713. https://doi.org/10.1038/s42003-021-02251-6.

13. Ku, M.W., Charneau, P., and Majlessi, L. (2021). Use of lentiviral vectors in vaccination. Expert Rev. Vaccines *20*, 1571–1586. https://doi.org/10.1080/14760584.2021.1988854.

14. Nemirov, K., Bourgine, M., Anna, F., Wei, Y., Charneau, P., and Majlessi, L. (2023). Lentiviral Vectors as a Vaccine Platform against Infectious Diseases. Pharmaceutics *15*, 846. https://doi.org/10.3390/pharmaceutics15030846.

15. (2011). A multi-center, randomized, double blind, placebo-controlled Phase I/II trial to compare the safety, tolerability and immunogenicity of the therapeutic THV01 vaccination at 5x10E6 TU, 5x10E7 TU, Clinical trials for 2011-006260-52. https://www.clinicaltrialsregister.eu/ctr-search/search?query=2011-006260-52.

16. Somaiah, N., Block, M.S., Kim, J.W., Shapiro, G.I., Do, K.T., Hwu, P., Eder, J.P., Jones, R.L., Lu, H., Ter Meulen, J.H., et al. (2019). First-in-Class, First-in-Human Study Evaluating LV305, a Dendritic-Cell Tropic Lentiviral Vector, in Sarcoma and Other Solid Tumors Expressing NY-ESO-1. Clin. Cancer Res. *25*, 5808–5817. https://doi.org/10.1158/1078-0432.CCR-19-1025.

17. Douguet, L., Fert, I., Lopez, J., Vesin, B., Le Chevalier, F., Moncoq, F., Authié, P., Nguyen, T.M., Noirat, A., Névo, F., et al. (2023). Full eradication of pre-clinical human papilloma virus-induced tumors by a lentiviral vaccine. EMBO Mol. Med. *15*, e17723. https://doi.org/10.15252/emmm.202317723.

18. Fert, I., Douguet, L., Vesin, B., Moncoq, F., Noirat, A., Authié, P., Ciret, S., Le Chevalier, F., Blanc, C., Vitrenko, Y., et al. (2024). T-cell immunity induced and reshaped by an anti-HPV immuno-oncotherapeutic lentiviral vector. NPJ Vaccines *9*, 102. https://doi.org/10.1038/s41541-024-00894-0.

19. Demidova, A., Douguet, L., Fert, I., Wei, Y., Charneau, P., and Majlessi, L. (2024). Comparison of preclinical efficacy of immunotherapies against HPV-induced cancers. Expert Rev. Vaccines *23*, 674–687. https://doi.org/10.1080/14760584.2024.2374287.

20. Cunha, A.C., Weigle, B., Kiessling, A., Bachmann, M., and Rieber, E.P. (2006). Tissue-specificity of prostate specific antigens: comparative analysis of transcript levels in prostate and non-prostatic tissues. Cancer Lett. *236*, 229–238. https://doi.org/10.1016/j.canlet.2005.05.021.

21. Ono, H., Yanagihara, K., Sakamoto, H., Yoshida, T., and Saeki, N. (2012). Prostate stem cell antigen gene is expressed in islets of pancreas. Anat. Cell Biol. *45*, 149–154. https://doi.org/10.5115/acb.2012.45.3.149.

22. Ross, S., Spencer, S.D., Holcomb, I., Tan, C., Hongo, J., Devaux, B., Rangell, L., Keller, G.A., Schow, P., Steeves, R.M., et al. (2002). Prostate stem cell antigen as therapy target: tissue expression and in vivo efficacy of an immunoconjugate. Cancer Res. *62*, 2546–2553.

23. Kong, H.Y., and Byun, J. (2013). Emerging roles of human prostatic Acid phosphatase. Biomol. Ther. *21*, 10–20. https://doi.org/10.4062/biomolther.2012.095.

24. Yeung, F., Li, X., Ellett, J., Trapman, J., Kao, C., and Chung, L.W. (2000). Regions of prostate-specific antigen (PSA) promoter confer androgen-independent expression of PSA in prostate cancer cells. J. Biol. Chem. *275*, 40846–40855. https://doi.org/10.1074/jbc.M002755200.

25. Gobin, S.J.P., Biesta, P., and Van den Elsen, P.J. (2003). Regulation of human beta 2-microglobulin transactivation in hematopoietic cells. Blood *101*, 3058–3064. https://doi.org/10.1182/blood-2002-09-2924.

26. van den Elsen, P.J. (2011). Expression regulation of major histocompatibility complex class I and class II encoding genes. Front. Immunol. *2*, 48. https://doi.org/10.3389/fimmu.2011.00048.

27. Vesin, B., Lopez, J., Noirat, A., Authié, P., Fert, I., Le Chevalier, F., Moncoq, F., Nemirov, K., Blanc, C., Planchais, C., et al. (2022). An intranasal lentiviral booster reinforces the waning mRNA vaccine-induced SARS-CoV-2 immunity that it targets to lung mucosa. Mol. Ther. *30*, 2984–2997. https://doi.org/10.1016/j.ymthe.2022.04.016.

28. Grossmann, M.E., Wood, M., and Celis, E. (2001). Expression, specificity and immunotherapy potential of prostate-associated genes in murine cell lines. World J. Urol. *19*, 365–370. https://doi.org/10.1007/pl00007104.

29. Griffith, T.S., Kawakita, M., Tian, J., Ritchey, J., Tartaglia, J., Sehgal, I., Thompson, T.C., Zhao, W., and Ratliff, T.L. (2001). Inhibition of murine prostate tumor growth and activation of immunoregulatory cells with recombinant canarypox viruses. J. Natl. Cancer Inst. *93*, 998–1007. https://doi.org/10.1093/jnci/93.13.998.

30. Blades, R.A., Keating, P.J., McWilliam, L.J., George, N.J., and Stern, P.L. (1995). Loss of HLA class I expression in prostate cancer: implications for immunotherapy. Urology *46*, 681–687. , discussion 686-687. https://doi.org/10.1016/S0090-4295(99)80301-X.

31. Kitamura, H., Torigoe, T., Asanuma, H., Honma, I., Sato, N., and Tsukamoto, T. (2007). Down-regulation of HLA class I antigens in prostate cancer tissues and up-regulation by histone deacetylase inhibition. J. Urol. *178*, 692–696. https://doi.org/10.1016/j.juro.2007.03.109.

32. Shaul, M.E., and Fridlender, Z.G. (2019). Tumour-associated neutrophils in patients with cancer. Nat. Rev. Clin. Oncol. *16*, 601–620. https://doi.org/10.1038/s41571-019-0222-4.

33. Corbett, T.H., Griswold, D.P., Jr., Roberts, B.J., Peckham, J.C., and Schabel, F.M., Jr. (1975). Tumor induction relationships in development of transplantable cancers of the colon in mice for chemotherapy assays, with a note on carcinogen structure. Cancer Res. *35*, 2434–2439.

34. Sheng, J., Chen, Q., Soncin, I., Ng, S.L., Karjalainen, K., and Ruedl, C. (2017). A Discrete Subset of Monocyte-Derived Cells among Typical Conventional Type 2 Dendritic Cells Can Efficiently Cross-Present. Cell Rep. *21*, 1203–1214. https://doi.org/10.1016/j.celrep.2017.10.024.

35. Allen, F., Bobanga, I.D., Rauhe, P., Barkauskas, D., Teich, N., Tong, C., Myers, J., and Huang, A.Y. (2018). CCL3 augments tumor rejection and enhances CD8(+) T cell infiltration through NK and CD103(+) dendritic cell recruitment via IFNgamma. Oncoimmunology *7*, e1393598. https://doi.org/10.1080/2162402X.2017.1393598.

36. Schaerli, P., and Moser, B. (2005). Chemokines: control of primary and memory T-cell traffic. Immunol. Res. *31*, 57–74. https://doi.org/10.1385/IR:31:1:57.

37. Zhang, W., Wang, H., Sun, M., Deng, X., Wu, X., Ma, Y., Li, M., Shuoa, S.M., You, Q., and Miao, L. (2020). CXCL5/CXCR2 axis in tumor microenvironment as potential diagnostic biomarker and therapeutic target. Cancer Commun. *40*, 69–80. https://doi.org/10.1002/cac2.12010.

38. Reschke, R., and Gajewski, T.F. (2022). CXCL9 and CXCL10 bring the heat to tumors. Sci. Immunol. *7*, eabq6509. https://doi.org/10.1126/sciimmunol.abq6509.

39. Hapuarachi, B., Danson, S., Wadsley, J., and Muthana, M. (2023). Exercise to transform tumours from cold to hot and improve immunotherapy responsiveness. Front. Immunol. *14*, 1335256. https://doi.org/10.3389/fimmu.2023.1335256.

40. Schenkel, J.M., Fraser, K.A., Beura, L.K., Pauken, K.E., Vezys, V., and Masopust, D. (2014). T cell memory. Resident memory CD8 T cells trigger protective innate and adaptive immune responses. Science *346*, 98–101. https://doi.org/10.1126/science.1254536.

41. Schenkel, J.M., Fraser, K.A., Vezys, V., and Masopust, D. (2013). Sensing and alarm function of resident memory CD8(+) T cells. Nat. Immunol. *14*, 509–513. https://doi.org/10.1038/ni.2568.

42. Vanderlugt, C.L., and Miller, S.D. (2002). Epitope spreading in immune-mediated diseases: implications for immunotherapy. Nat. Rev. Immunol. *2*, 85–95. https://doi.org/10.1038/nri724.

43. Lopez, J., Anna, F., Authié, P., Pawlik, A., Ku, M.W., Blanc, C., Souque, P., Moncoq, F., Noirat, A., Hardy, D., et al. (2022). A lentiviral vector encoding fusion of light invariant chain and mycobacterial antigens induces protective CD4(+) T cell immunity. Cell Rep. *40*, 111142. https://doi.org/10.1016/j.celrep.2022.111142.

44. Cousin, C., Oberkampf, M., Felix, T., Rosenbaum, P., Weil, R., Fabrega, S., Morante, V., Negri, D., Cara, A., Dadaglio, G., and Leclerc, C. (2019). Persistence of Integrase-Deficient Lentiviral Vectors Correlates with the Induction of STING-Independent CD8(+) T Cell Responses. Cell Rep. *26*, 1242–1257.e7. https://doi.org/10.1016/j.celrep.2019.01.025.

45. Goloudina, A., Le Chevalier, F., Authié, P., Ciret, S., Nemirov, K., Fert, I., Moncoq, F., Vesin, B., Noirat, A., Blanc, C., et al. (2025). A lentiviral vector targeting a KRAS

neoepitope for cancer immunotherapy. Sci. Rep. *15*, 23171. https://doi.org/10.1038/s41598-025-05134-6.

46. Ku, M.W., Authié, P., Bourgine, M., Anna, F., Noirat, A., Moncoq, F., Vesin, B., Nevo, F., Lopez, J., Souque, P., et al. (2021). Brain cross-protection against SARS-CoV-2 variants by a lentiviral vaccine in new transgenic mice. EMBO Mol. Med. *13*, e14459. https://doi.org/10.15252/emmm.202114459.

47. Lawrence, M.S., Stojanov, P., Polak, P., Kryukov, G.V., Cibulskis, K., Sivachenko, A., Carter, S.L., Stewart, C., Mermel, C.H., Roberts, S.A., et al. (2013). Mutational heterogeneity in cancer and the search for new cancer-associated genes. Nature *499*, 214–218. https://doi.org/10.1038/nature12213.

48. Abida, W., Cheng, M.L., Armenia, J., Middha, S., Autio, K.A., Vargas, H.A., Rathkopf, D., Morris, M.J., Danila, D.C., Slovin, S.F., et al. (2019). Analysis of the Prevalence of Microsatellite Instability in Prostate Cancer and Response to Immune Checkpoint Blockade. JAMA Oncol. *5*, 471–478. https://doi.org/10.1001/jamaoncol.2018.5801.

49. Wang, J., Zhou, K., Zhu, H., Wei, F., Ma, S., Kan, Y., Li, B., and Mao, L. (2023). Current status and progress of the development of prostate cancer vaccines. J. Cancer *14*, 835–842. https://doi.org/10.7150/jca.80803.

50. Sutherland, S.I.M., Ju, X., Horvath, L.G., and Clark, G.J. (2021). Moving on From Sipuleucel-T: New Dendritic Cell Vaccine Strategies for Prostate Cancer. Front. Immunol. *12*, 641307. https://doi.org/10.3389/fimmu.2021.641307.

51. Rodems, T.S., Heninger, E., Stahlfeld, C.N., Gilsdorf, C.S., Carlson, K.N., Kircher, M.R., Singh, A., Krueger, T.E.G., Beebe, D.J., Jarrard, D.F., et al. (2022). Reversible epigenetic alterations regulate class I HLA loss in prostate cancer. Commun. Biol. *5*, 897. https://doi.org/10.1038/s42003-022-03843-6.

52. Seliger, B., Stoehr, R., Handke, D., Mueller, A., Ferrone, S., Wullich, B., Tannapfel, A., Hofstaedter, F., and Hartmann, A. (2010). Association of HLA class I antigen abnormalities with disease progression and early recurrence in prostate cancer. Cancer Immunol. Immunother. *59*, 529–540. https://doi.org/10.1007/s00262-009-0769-5.

53. Bhatia, V., Kamat, N.V., Pariva, T.E., Wu, L.T., Tsao, A., Sasaki, K., Sun, H., Javier, G., Nutt, S., Coleman, I., et al. (2023). Targeting advanced prostate cancer with STEAP1 chimeric antigen receptor T cell and tumor-localized IL-12 immunotherapy. Nat. Commun. *14*, 2041. https://doi.org/10.1038/s41467-023-37874-2.

54. Colomer-Lluch, M., Ruiz, A., Moris, A., and Prado, J.G. (2018). Restriction Factors: From Intrinsic Viral Restriction to Shaping Cellular Immunity Against HIV-1. Front. Immunol. *9*, 2876. https://doi.org/10.3389/fimmu.2018.02876.

55. Rodriguez, M., Sarry, E., Bejanariu, A., Casaban, L., Abdalla, S., Sabbah-Petrover, E., and Bauche, C. (2012). Design and development of a new lentiviral based anti-HIV therapeutic vaccine. Retrovirology *13*, 350.

56. Christensen, A.H., and Quail, P.H. (1996). Ubiquitin promoter-based vectors for high-level expression of selectable and/or screenable marker genes in monocotyledonous plants. Transgenic Res. *5*, 213–218. https://doi.org/10.1007/BF01969712.

57. Wiborg, O., Pedersen, M.S., Wind, A., Berglund, L.E., Marcker, K.A., and Vuust, J. (1985). The human ubiquitin multigene family: some genes contain multiple directly repeated ubiquitin coding sequences. EMBO J. *4*, 755–759. https://doi.org/10.1002/j.1460-2075.1985.tb03693.x.

58. Ku, M.W., Bourgine, M., Authié, P., Lopez, J., Nemirov, K., Moncoq, F., Noirat, A., Vesin, B., Nevo, F., Blanc, C., et al. (2021). Intranasal vaccination with a lentiviral vector protects against SARS-CoV-2 in preclinical animal models. Cell Host Microbe *29*, 236–249.e6. https://doi.org/10.1016/j.chom.2020.12.010.

OMTON, Volume *34*

Supplemental information

Eradication of pre-clinical prostate tumors

by a lentiviral vector-based immunotherapy

Benjamin Vesin, Ingrid Fert, Sylvain Ciret, Laëtitia Douguet, Fanny Moncoq, Amandine Noirat, Pierre Authié, Fabien Le Chevalier, Fabien Nevo, Catherine Blanc, Kirill Nemirov, Laleh Majlessi, and Pierre Charneau

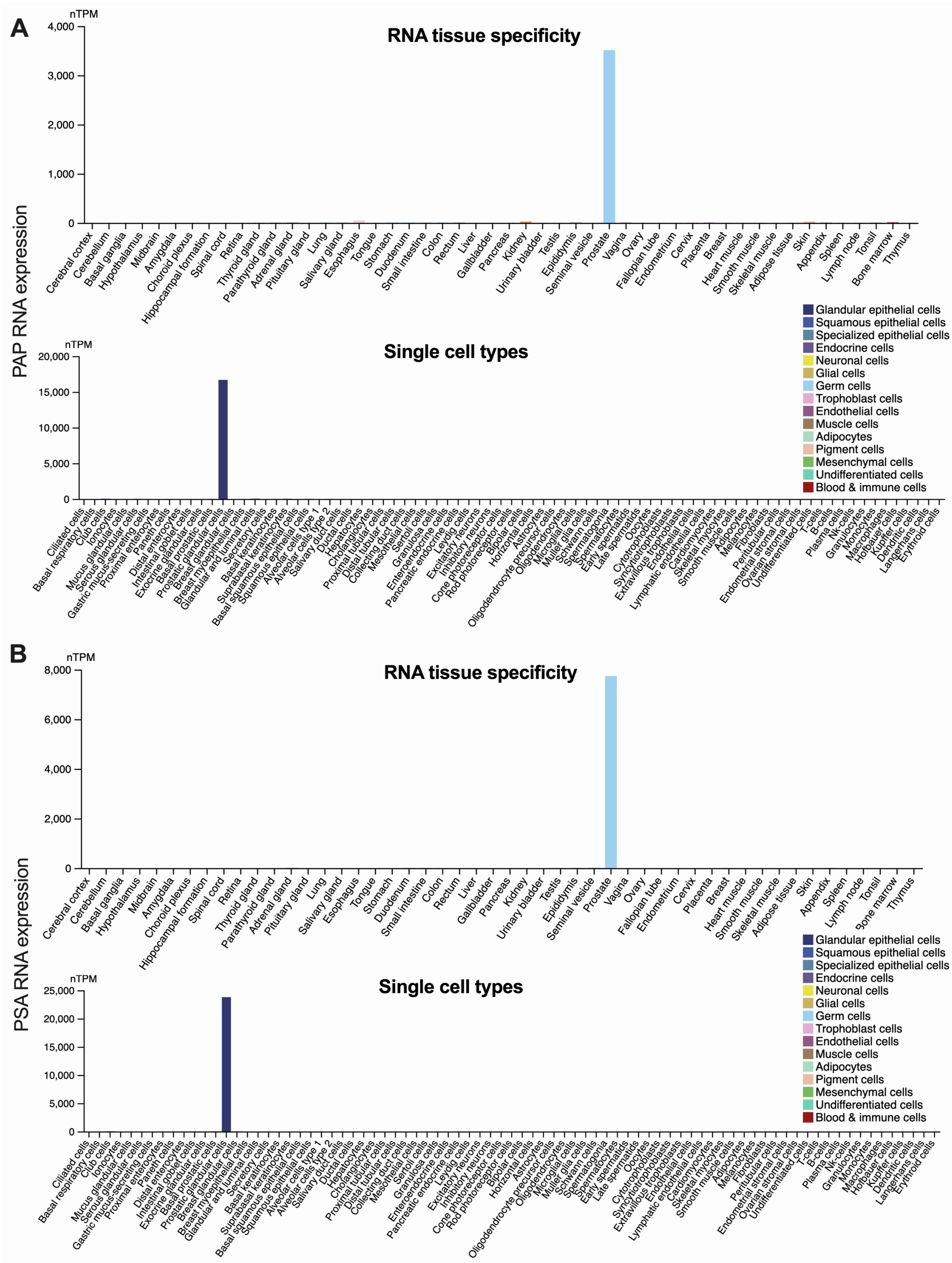

Figure S1. PAP and PSA mRNA expression in human organs. (A-B, top) Consensus specificity of PAP (https://www.proteinatlas.org/ENSG00000014257-ACP3/tissue#rna_expression) and PSA (https://www.proteinatlas.org/ENSG00000142515-KLK3/tissue) mRNA expression in human organs. **(A-B, bottom)** Specificity of PAP (https://www.proteinatlas.org/ENSG00000014257-ACP3/single+cell+type) and PSA (https://www.proteinatlas.org/ENSG00000142515-KLK3/single+cell+type) mRNA expression in cell types.[1,2]

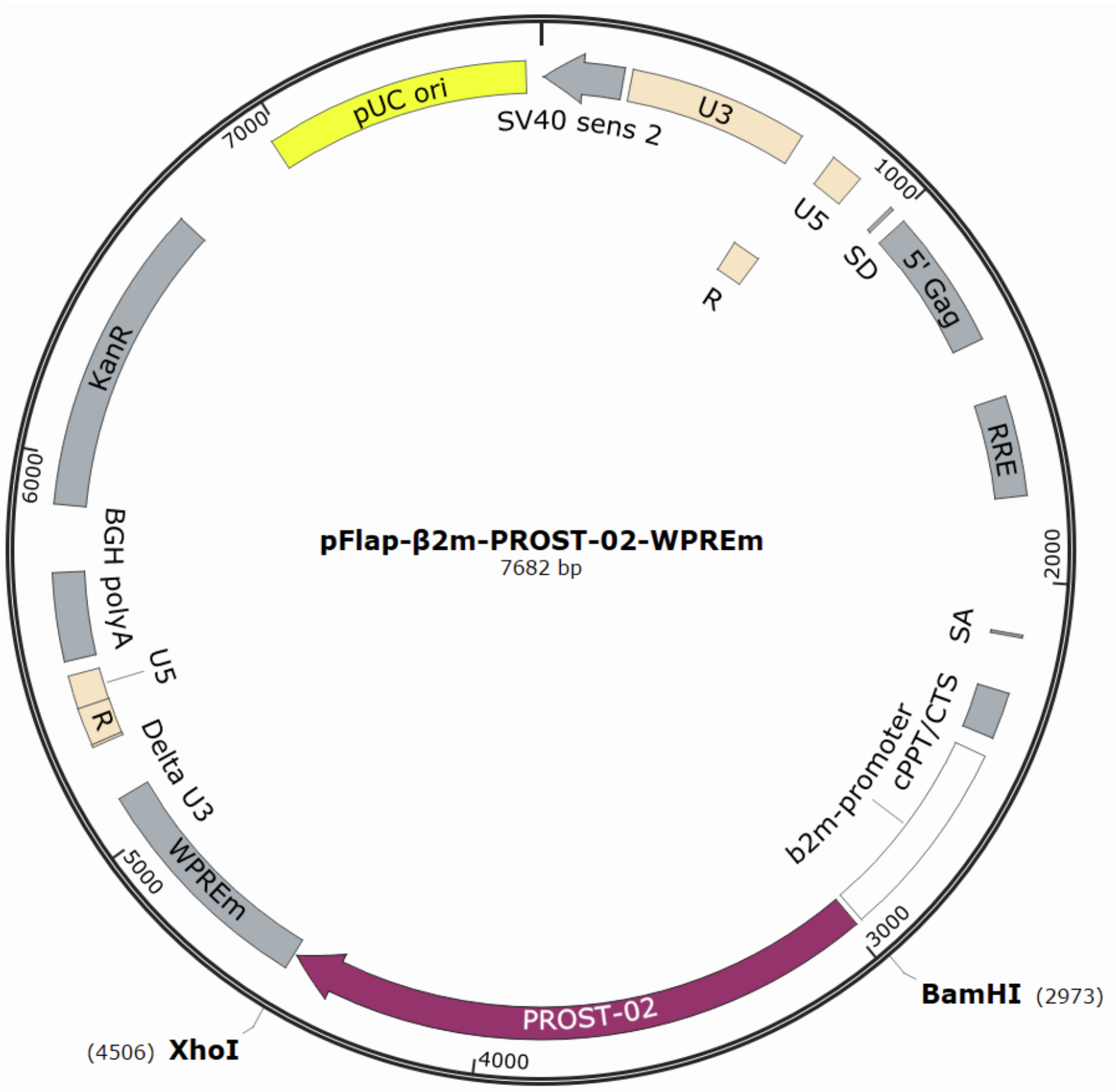

PROST-02 nucleotide sequence (1521 bp)

ATGAGGGCCGCCCCTTTGTTGTTGGCTAGGGCCGCCTCCCTGTCCCTGGGCTTCCTGTTCCTGCTGTTCTTCTGGCTGGA
CCGGTCCGTGTTGGCCAAGGAGCTGAAGTTCGTGACCCTGGTGTTTCGGCATGGCCACTACGAGTTGGGAGAATATATCC
GCAAGAGGTACCGCAAGTTCCTGAACGAGTCTTACAAGCACGAGCAGGTTTACATCAGGTCCACCGACGTGGACAGGACT
CTCATGTCCGCTATGACCAACCTGGCCGCACTGTTCCCCCCCGAAGGCGTGTCCATCTGGAACCCCATCCTCCTGTGGCA
GCCCATCCCCGTGCACACCGTGCCCCTGAGCGAGGACCAGCTGCTGTACCTGCCCTTCCGCAATTGCCCCCGTTTCCAGG
AGCTGAGACTCCACCCCTACAAGGACTTCATTGCCACCCTCGGCAAGTTGTCCGGCCTGCACGGCCAGGATTTGTTCGGC
ATTTGGTCCAAAGTCTATGACCCCCTCTACTGCGAATCCGTGCACAACTTCACCCTCCCCTCCTGGGCCACCGAAGACAC
CATGACCAAGCTGAGGGAGCTGTCCGAGCTGAGTTTGCTGTCCCTGTACGGCATCCACAAACAGAAGGAGAAATCCCATA
TGAAAAGGGCCACCCAGATCCCCTCCTACAAGAAACTGATCATGTACTCCGCCCACGACACCACAGTCTCCGGCCTCCAG
ATGGCCCTCGACGTCTACAACGGACTGCTGCCCCCCTACGCCTCCTGTCACCTCACCGAACTCTACTTCGAAAAGGGCGA
ATACTTCGTGGAAATGTACTACAGGAACGAAACCCAGCACGAACCCTATCCCCTCATGCTCCCCGGCCCCGACGACTGGG
TGCCTGTTGTCTTCCTGACTTTGTCCGTGACCTGGATCGGCGCTGCCCCTCTGATCTTGTCCCGGATTGTGGCGGCTGG
GAGTGCGAAAAACACTCCCAGAGGGGCCGGGCTGTGTGTGGCGGTGTGCTGGTGCATCCTCAGTGGGTGCTGACTGCTGC
TCATTGCATTAGGAACAAGAGTGTGATCTTGCTGGGGAGGCACTCCTTGTTCCACCCCGAGGATACTGGTCAGGTGTTTC
AGGTGTCCCATTCCTTCCCCCACCCCCTCTACGACATGTCCCTGCTGAAGAACAGATTCTTGCGCCCCGGCGACGACTCC
TCCCACGACTTGATGCTGCTGCGGCTCGGCCCCGACGATATGGACCTCCCCACCCAGGAGCCCGCCCTGGGAACAACCTG
CTACGCCTCCGGCTGGGGCTCCATCGAGCCCGAAGAGTTCCTCACCCCCAAGAAACTGCAGTGCGTTGACCTGCACGTGA
TTTCCAACGACGTGTGCGCCCAGGTGCACCCCCAGAAGGTGACCAAGTTCATGCTGTGCGCCGGCAGGTGGACAGGCGGC
TCTGAACCCTGCGCCCTCCCCGAAAGACCCTCCCTGTACACCAAAGTGGTGCACTATAGGAAGTGGATCAAGGACTGATA
A

Figure S2. Map of the plasmid encoding PROST-02 antigenic fusion protein. The codon-optimized cDNA sequence, encoding PROST-02 (PAP-PSA), indicated in Fig. 1 was inserted under the β2m promoter in a lentiviral pFLAP backbone plasmid. The PROST-02 nucleic acid sequence is indicated, bp = base pair.

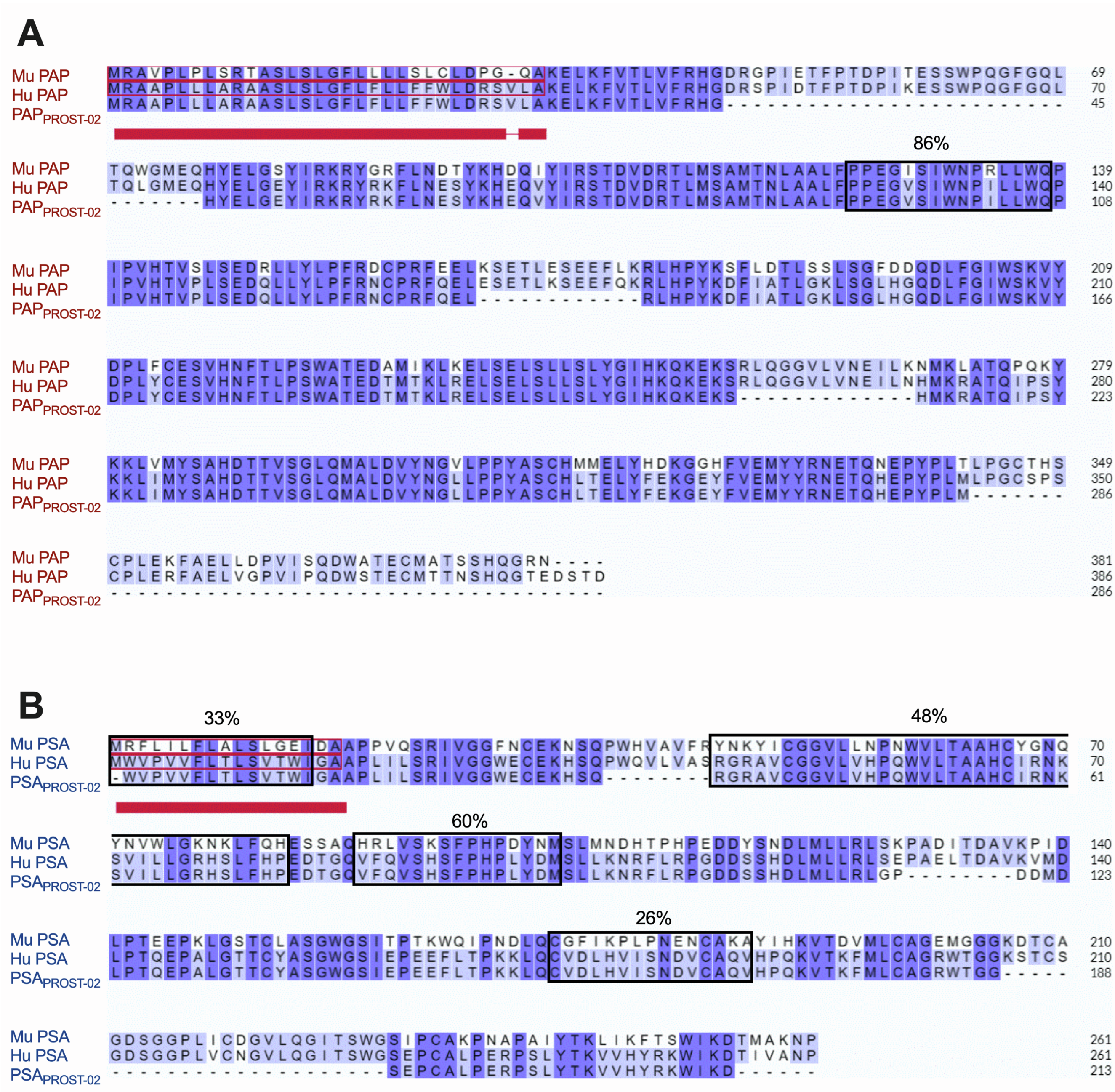

Figure S3. Sequence comparison of murine, human and Lenti-PROST-02-encoded PAP and PSA antigens. (A) Aa sequence alignment of PAP, murine (first line, "Mu") (Accession no Q8CE08), human (second line, "Hu") (Accession no P15309), and encoded by Lenti-PROST-02 (third line). **(B)** Aa sequence alignment of PSA, murine (first line, "Mu") (Accession no Q61759), human (second line, "Hu") (Accession no P07288), and encoded by Lenti-PROST-02 (third line). Immunogenic regions identified by epitope mapping in C57BL/6 (H-2^b) mice (Fig. 2B) are outlined in black. Aa highlighted in dark or pale purple are identical for the three or two sequences, respectively. For each immunogenic region, the percentages correspond to the sequence identity between the murine ortholog and the sequence encoded by Lenti-PROST-02. The N-terminal red lines indicate the secretory signal sequence. Analysis was performed using Uniprot Blast (https://www.uniprot.org/blast).

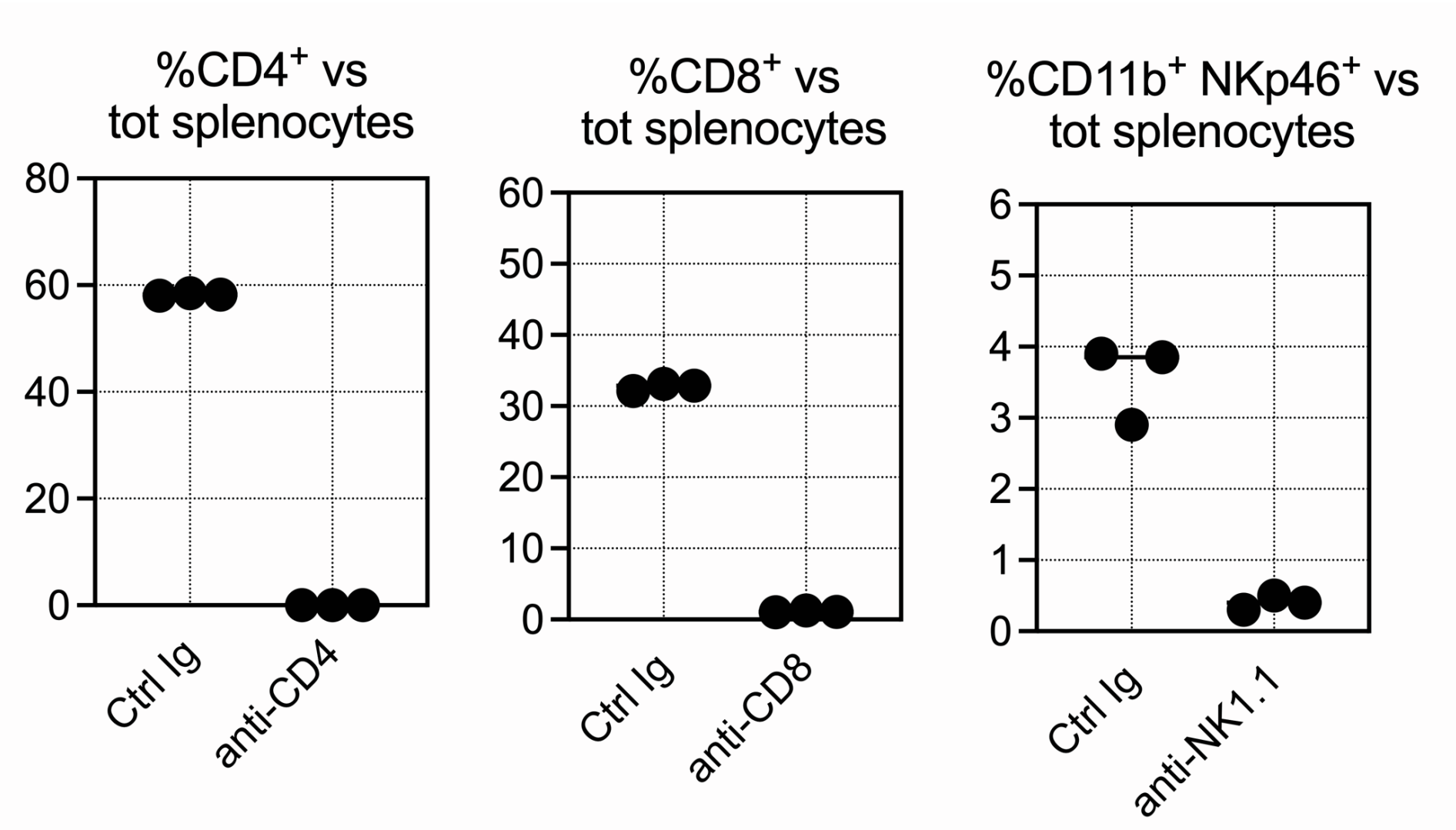

Figure S4. Efficacy of anti-CD4, anti-CD8 or anti-NK1.1 depletion in vivo. Tumor-free C57BL/6 mice (*n* = 3/group) received repeated i.p. injections of 200 µg anti-CD4 (clone GK1.5), anti-CD8 (clone H35.17.2), anti-NK1.1 (PK136) or a control Ig isotype. On day 3 after the last injection, the spleens from individual mice were assessed by anti-CD3, anti-CD4, and anti-CD8 or anti-CD11b and anti-NKp46 mAb staining and cytometry analysis to evaluate the efficacy of cell subset depletion. The results from 3 individual mice/group are shown.

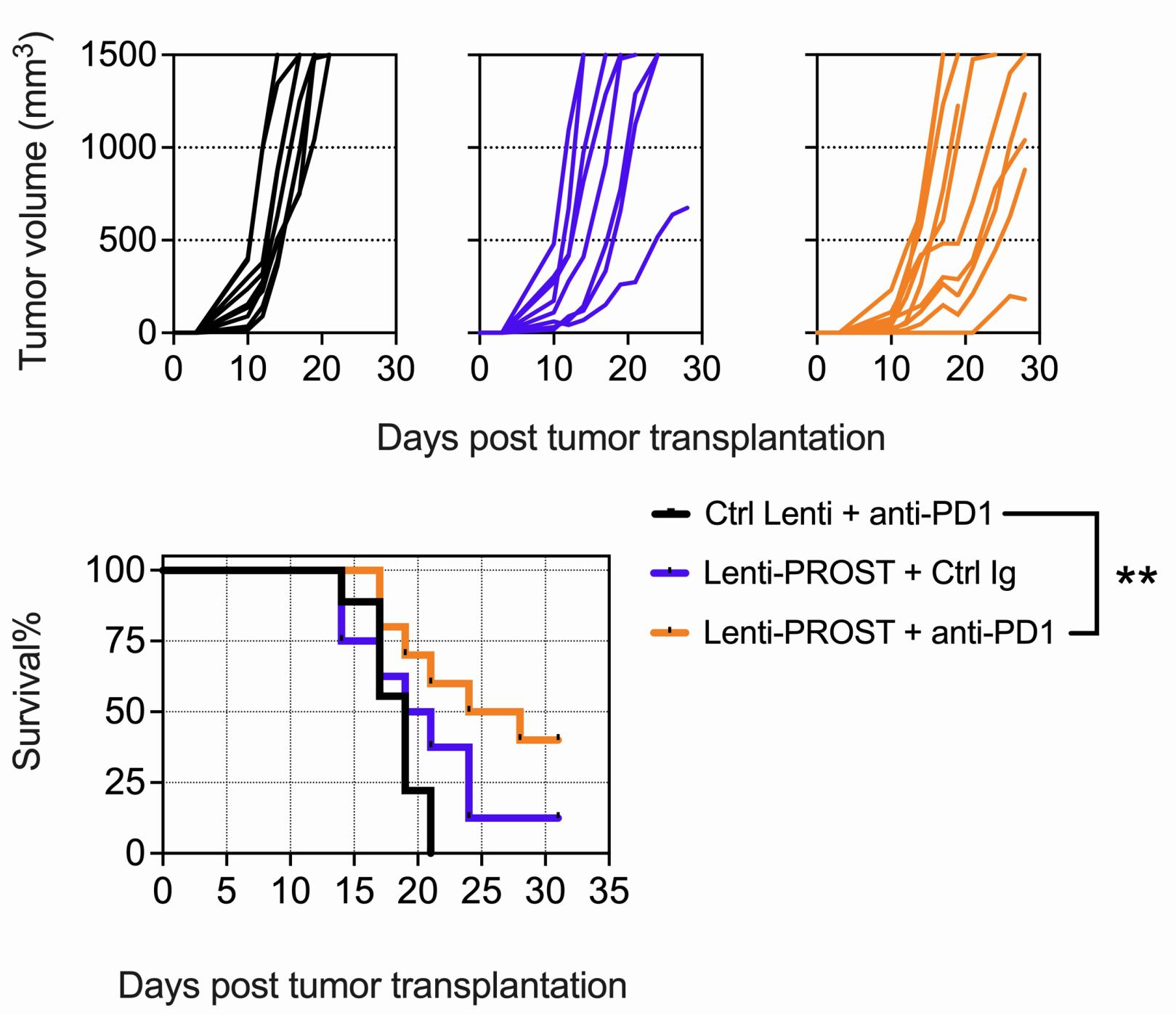

Figure S5. Lentiviral vector-based immunotherapy of tumors expressing human PAP. C57BL/6 male mice (n = 7-9/group) were engrafted s.c. on the flank with 2×10^5 of the MC38-PAP tumor cells. On day 6 post tumor engraftment, mice were randomized by tumor size and injected i.m. with the vaccine or Ctrl Lenti. Starting on day 10, 4 days after vaccination, mice received intraperitoneal injections of 200 µg/mouse/injection of anti-PD1 mAb (clone RMP1-14) or Ctrl Ig every 2 days. Tumor volume versus time (top). Survival of animals (bottom). Statistical significance was determined by log-rank Mantel-Cox test (**$p < 0.01$). Mice were sacrificed when tumors reached 1500 mm^3.

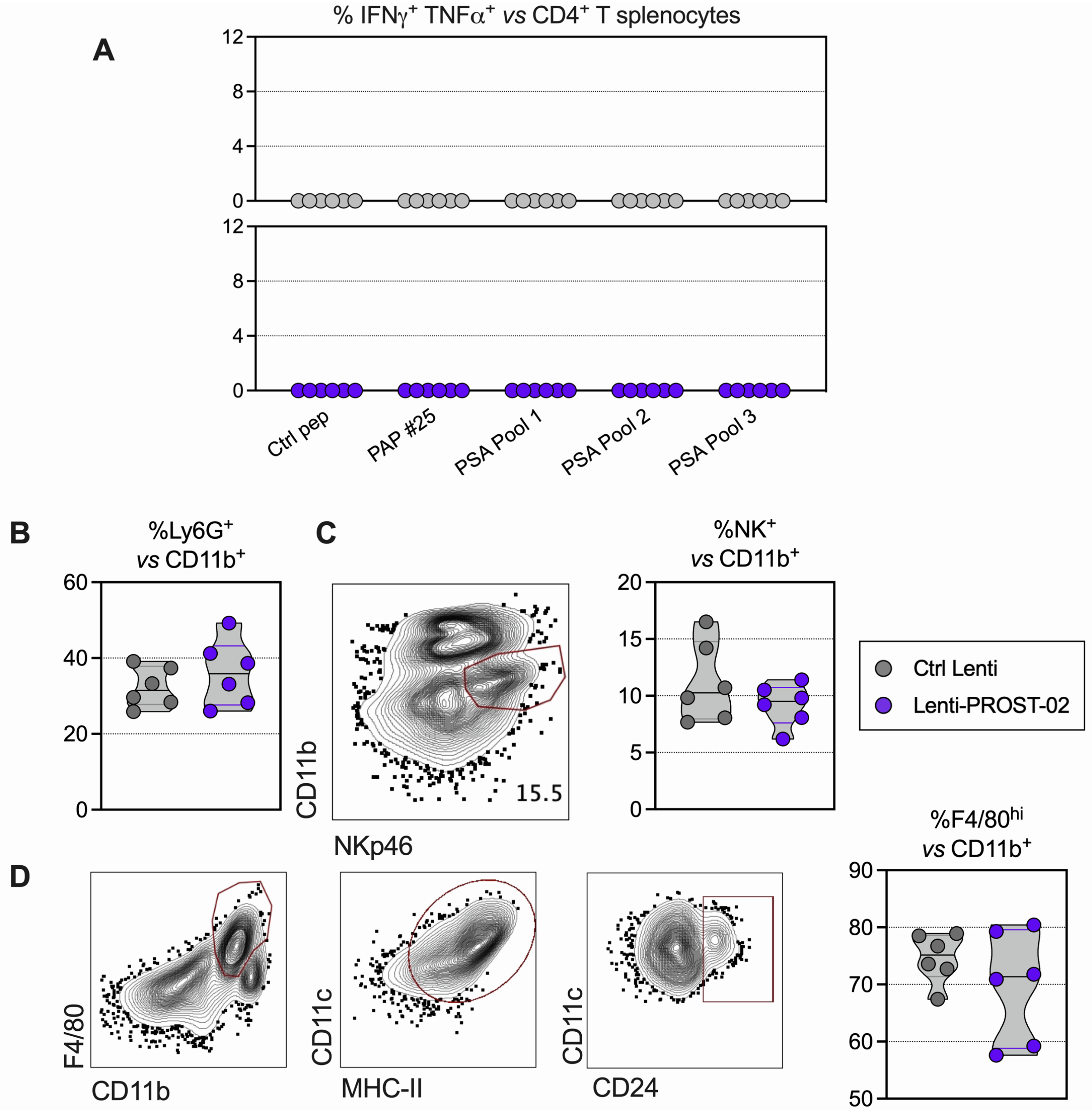

Figure S6. Additional immunological features of Ctrl Lenti- or Lenti-PROST-02-treated mice. **(A)** Absence of CD4$^+$ T splenocyte responses in 3E9-bearing mice treated with Ctrl Lenti or Lenti-PROST-02, as determined by ICS. **(B-D)** Cytometric analysis of innate immune cells in the 3E9 tumors of Ctrl Lenti- or Lenti-PROST-02-treated mice. The tumors were from the mice described in the Figure 4. Frequencies of granulocytes/neutrophils **(B)**, as cytometrically defined in the Figure 3B, **(C)** NK cells, and **(D)** F4/80^{hi} cells in the tumors of Ctrl Lenti- or Lenti-PROST-02-treated mice, on day 9 post treatment. Note that the F4/80^{hi} CD11b^{int} cells were CD11c$^+$ and MHC-II$^+$ and contained a subset of CD24$^+$ cells. Differences between the two groups were not statistically significant, determined by two-tailed Mann-Whitney test.

Table S1. HLA-1 alleles considered for epitope prediction by IEDB on human PSA and PAP sequences.

HLA-A	HLA-B
HLA-A*01:01	HLA-B*07:02
HLA-A*02:01	HLA-B*08:01
HLA-A*02:03	HLA-B*15:01
HLA-A*02:06	HLA-B*35:01
HLA-A*03:01	HLA-B*40:01
HLA-A*11:01	HLA-B*44:02
HLA-A*23:01	HLA-B*44:03
HLA-A*24:02	HLA-B*51:01
HLA-A*26:01	HLA-B*53:01
HLA-A*30:01	HLA-B*57:01
HLA-A*30:02	HLA-B*58:01
HLA-A*31:01	
HLA-A*32:01	
HLA-A*33:01	
HLA-A*68:01	
HLA-A*68:02	

These 27 HLA-1 alleles considered account for 97% of the HLA-A and HLA-B allelic variants in most human ethnicities.[3,4]

Table S2. Features of PAP and PSA epitopes mapped to PROST-02 antigen. Characteristics of PAP- and PSA-derived T-cell epitopes mapped to the human PROST-02 antigen in the murine H-2^b haplotype (Figure 2B). The human and murine sequences of the regions containing these epitopes were compared to visualize aa sequence identity. The fine epitope regions of the human sequences and their orthologous murine regions are highlighted in gray. Predicted binding scores of these sequences to H-2K^b and H-2D^b, as predicted by SYFPEITHI (http://www.syfpeithi.de), are shown. The anchor residues are indicated in bold.

Prostate antigen	Pep	aa sequence	Identity	H-2K^b epitope	H-2K^b score*	H-2D^b epitope	H-2D^b score*
Hu PSA<br>Mu PSA	PAP #25	PPEGVSIWNPILLWQ<br>PPEGISIWNPRLLWQ	7/9	VSIW**N**PIL**L**	26	SIWN**P**IL**L**	13
Hu PSA<br>Mu PSA	PSA #1	MWVPVVFLTLSVTWI<br>MRFLILFLALSLGEI	3/9	WVPV**V**FLT**L**	17	VPVV**F**LT**L**	21
Hu PSA<br>Mu PSA	PSA #10	RGRAVCGGVLVHPQ<br>YNKYICGGVLLNPN	5/9	GRAV**C**GGV**L**	12	RAVC**G**GV**L**	12
Hu PSA<br>Mu PSA	PSA #14	LTAAHCIRNKSVILL<br>LTAAHCYGNQYNVWL	4/11	HCIR**N**KSV**I**	24	RNKS**V**IL**L**	13
Hu PSA<br>Mu PSA	PSA #16	NKSVILLGRHSLFHP<br>NQYNVWLGKNKLFQH	3/9	VILL**G**RHS**L**	13	ILLG**R**HS**L**	11
Hu PSA<br>Mu PSA	PSA #21	VFQVSHSFPHPLYDM<br>HRLVSKSFPHPDYNM	7/9	SFPH**P**LYD**M**	16	VSHS**F**PH**P**	11
Hu PSA<br>Mu PSA	PSA #40	CVDLHVISNDVCAQV<br>CGFIKPLPNENCAKA	3/11	SNDV**C**AQ**V**	9	HVIS**N**DVC**A**	13

*The score is defined by the probability for an aa sequence of being processed and presented by MHC molecules.

Table S3. Sequences of primers used in qRT-PCR.

Gene	cDNA accession number	Forward sequence (5' to 3')	Reverse sequence (5' to 3')	Amplicon (bp)
SDHA	NM_023281.1	TGCCCTGAGCATTGCAGAAT	TGCAATACACTCCCCACACG	198
IFNα	NM_010502.2	GGATGTGACCTTCCTCAGACTC	ACCTTCTCCTGCGGGAATCCAA	131
IFNγ	NM_008337.4	TCAAGTGGCATAGATGTGGAAGAA	TGGCTCTGCAGGATTTTCATG	92
TNFα	NM_013693	CATCTTCTCAAAATTCGAGTGACAA	TGGGAGTAGACAAGGTACAACCC	175
TGFβ	NM_011577.2	TGACGTCACTGGAGTTGTACGG	GGTTCATGTCATGGATGGTGC	170
IL-1β	NM_008361.4	TGCCACCTTTTGACAGTGATG	AAGGTCCACGGGAAAGACAC	220
IL-2	NM_008366.3	CCTGAGCAGGATGGAGAATTACA	TCCAGAACATGCCGCAGAG	141
IL-4	NM_021283.2	GAGCTCGTCTGTAGGGCTTC	TGCTCTTTAGGCTTTCCAGG	205
IL-5	NM_010558.1	GATGAGGCTTCCTGTCCCTACT	TGACAGGTTTTGGAATAGCATTTCC	130
IL-6	NM_031168.2	CTGCAAGTGCATCATCGTTGTTC	TACCACTTCACAAGTCGGAGGC	116
IL-10	NM_010548.2	ATGCTGCCTGCTCTTACTGAC	TCCAGCTGGTCCTTTGTTTGA	165
IL-12p40	NM_001303244.1	GGAAGCACGGCAGCAGAATA	AACTTGAGGGAGAAGTAGGAATGG	180
IL-18	NM_008360.2	GACAGCCTGTGTTCGAGGATATG	TGTTCTTACAGGAGAGGGTAGAC	159
IL-33	NM_001164724.2	CTACTGCATGAGACTCCGTTCTG	AGAATCCCGTGGATAGGCAGAG	136
CCL2	NM_011333.3	AGGTCCCTGTCATGCTTCTG	TCTGGACCCATTCCTTCTTG	249
CCL3	NM_011337.2	CCTCTGTCAC CTGCTCAACA	GATGAATTGGCGTGGAATCT	163
CCL5	NM_013653.3	GTGCCCACGTCAAGGAGTAT	GGGAAGCTATACAGGGTCA	185
CCL19	NM_011888.4	CTGCCTCAGATTATCTGCCAT	AGGTAGCGGAAGGCTTTCAC	177
CCL21	NM_002989.4	AAGGCAGTGATGGAGGGG	CGGGGTAAGAACAGGATTG	136
CXCL5	NM_009141.3	GCGTTGTGTTTGCTTAACCG	GAACACTGGCCGTTCTTTCC	242
CXCL9	NM_008599.4	AAAATT TCATCACGCCCTTG	TCTCCAGCTTGGTGAGGTCT	207
CXCL10	NM_021274.2	TGCCGTCATTTTCTGCCTCA	AGGCTCGCAGGGATGATTTC	150

References

1. Karlsson, M., Zhang, C., Mear, L., Zhong, W., Digre, A., Katona, B., Sjostedt, E., Butler, L., Odeberg, J., Dusart, P., et al. (2021). A single-cell type transcriptomics map of human tissues. Sci Adv *7*. 10.1126/sciadv.abh2169.
2. Uhlen, M., Fagerberg, L., Hallstrom, B.M., Lindskog, C., Oksvold, P., Mardinoglu, A., Sivertsson, A., Kampf, C., Sjostedt, E., Asplund, A., et al. (2015). Proteomics. Tissue-based map of the human proteome. Science *347*, 1260419. 10.1126/science.1260419.
3. Sette, A., and Sidney, J. (1999). Nine major HLA class I supertypes account for the vast preponderance of HLA-A and -B polymorphism. Immunogenetics *50*, 201-212. 10.1007/s002510050594.
4. Weiskopf, D., Angelo, M.A., de Azeredo, E.L., Sidney, J., Greenbaum, J.A., Fernando, A.N., Broadwater, A., Kolla, R.V., De Silva, A.D., de Silva, A.M., et al. (2013). Comprehensive analysis of dengue virus-specific responses supports an HLA-linked protective role for CD8+ T cells. Proc Natl Acad Sci U S A *110*, E2046-2053. 10.1073/pnas.1305227110.
